# Supplementary material for: Development and validation of the Multimorbidity Treatment Burden Questionnaire (MTBQ)
Source: BMJ Open. 2018 Apr 12;8(4):e019413. doi: 10.1136/bmjopen-2017-019413 (PMC5900423; doi:10.1136/bmjopen-2017-019413)
Supplement: Supplementary file 1 [file bmjopen-2017-019413supp001.pdf]

Appendix A: Characteristics of the participants who took part in the cognitive interviews (n=8)

Characteristic	Value
Mean age years (SD, min, max)	55.5 (14.1, 30, 78)
Male	2 (25%)
White British ethnicity	8 (100%)
Mean number of self-reported long-term conditions (SD, min, max)	2.1 (1.5, 1, 5)

Appendix B: Cronbach's alpha including the optional questions (questions 3, 9 and 10) in the various combinations

	Optional questions						
	**3**	**9**	**10**	**3, 9, 10**	**3, 9**	**3, 10**	**9, 10**
**Cronbach's alpha**	0.82	0.83	0.83	0.84	0.83	0.83	0.84

Optional questions: Please tell us how much difficulty you have with the following:

Question 3. Paying for prescriptions, over the counter medication or equipment
Question 9. Getting health care in the evenings and at weekends
Question 10. Getting help from community services (e.g. physiotherapy, district nurses etc)

Appendix C: A comparison of the floor effects and missing data of the MTBQ and the HCTD (pilot study data)

MTBQ Question	Floor effect[a] %	Missing data %	HCTD question with a similar latent construct	Floor effect [b] %	Missing data (%)
**1.** Taking lots of medications	78	1	3. Difficulty taking medications	95	1
**2.** Remembering how and when to take medication	80	1	2. Difficulty planning medication schedule	94	3
3. *Paying for prescriptions, over the counter medication or equipment*	94	4	5. Difficulty paying prescription charges	78	19
**4.** Collecting prescription medication	83	2	1. Difficulty obtaining medications	87	1
**5.** Monitoring your medical conditions (eg. checking your blood pressure or blood sugar, monitoring your symptoms etc)	83	2	No question to compare with		
**6.** Arranging appointments with health professionals	59	3	6. Difficulty scheduling medical appointment	69	4
**7.** Seeing lots of different health professionals	62	2	No question to compare with		
**8.** Attending appointments with health professionals (eg. getting time off work, arranging transport etc)	74	1	7. Difficulty arranging transportation	76	6
9. *Getting health care in the evenings and at weekends*	70	3	No question to compare with		
10. *Getting help from community services (eg. physiotherapy, district nurses etc)*	83	2	No question to compare with		
**11.** Obtaining clear and up-to-date information about your condition	70	2	8. Difficulty getting information	74	4
**12.** Making recommended lifestyle changes (eg. diet and exercise)	57	3	No question to compare with		
**13.** Having to rely on help from family and friends	69	1	No question to compare with		

[a] proportion (%) of 'does not apply' or 'not difficult' responses

[b] proportion (%) 'not difficult' responses

Please note: Questions 3, 9 and 10 were excluded from the main analysis due to a high proportion of 'does not apply' responses. They are shown in italics. As they may be relevant to other populations, they can be considered as optional

Appendix D: Histogram of global MTBQ scores and global HCTD scores (pilot study and main study)

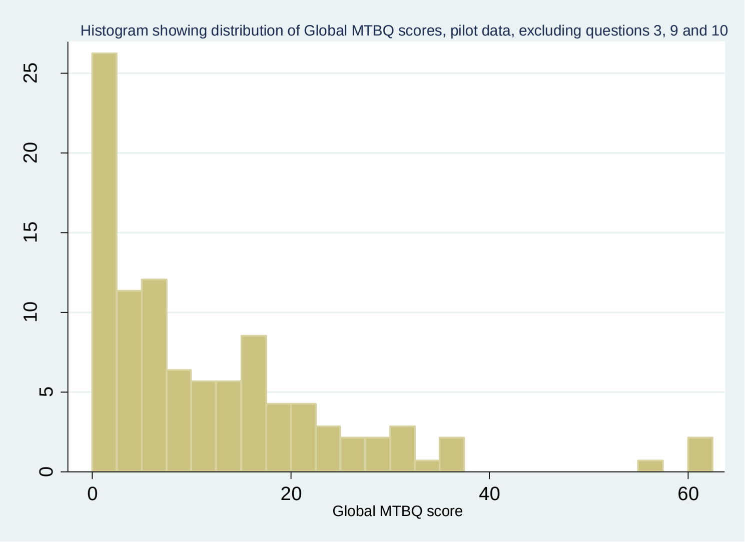

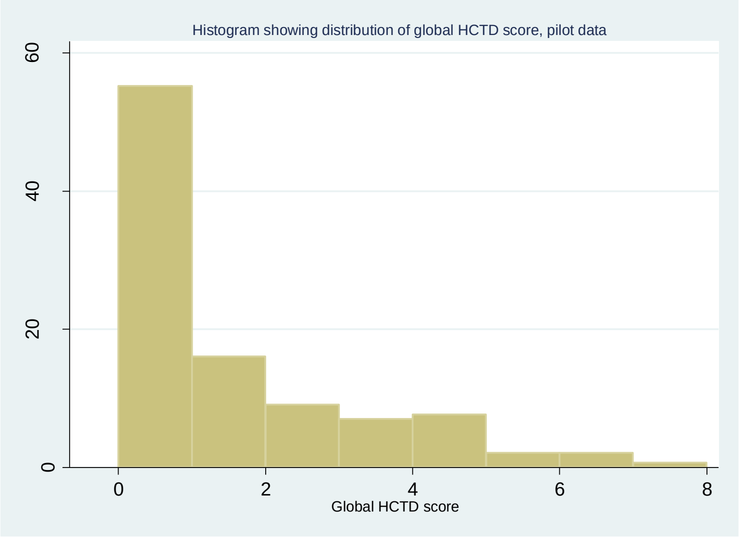

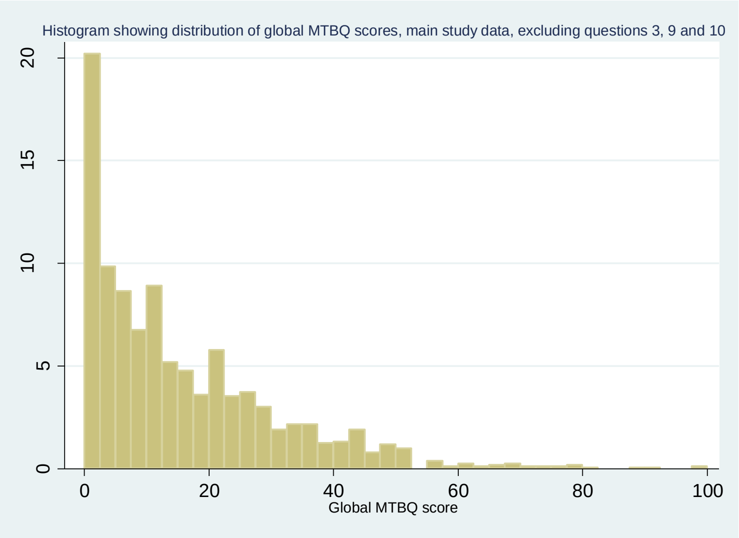

Appendix E: Inter-item correlation coefficient and Cronbach's Alpha (main study data, excluding questions 3, 9 and 10)

Cronbach's alpha = 0.83

Question:	1	2	4	5	6	7	8	11	12	13
**1**	1.00									
**2**	0.69	1.00								
**4**	0.30	0.26	1.00							
**5**	0.35	0.33	0.31	1.00						
**6**	0.26	0.23	0.28	0.31	1.00					
**7**	0.34	0.29	0.29	0.38	0.62	1.00				
**8**	0.32	0.32	0.40	0.33	0.37	0.44	1.00			
**11**	0.24	0.19	0.27	0.27	0.45	0.46	0.33	1.00		
**12**	0.28	0.27	0.23	0.32	0.29	0.34	0.31	0.35	1.00	
**13**	0.32	0.25	0.30	0.26	0.28	0.34	0.40	0.29	0.33	1.00

Questions: Please tell us how much difficulty you have with the following:

1. Taking lots of medications
2. Remembering how and when to take medication
4. Collecting prescription medication
5. Monitoring your medical conditions (eg. checking your blood pressure or blood sugar, monitoring your symptoms etc)
6. Arranging appointments with health professionals
7. Seeing lots of different health professionals
8. Attending appointments with health professionals (eg. getting time off work, arranging transport etc)
11. Obtaining clear and up-to-date information about your condition
12. Making recommended lifestyle changes (eg. diet and exercise)
13. Having to rely on help from family and friends

Please note: Questions 3, 9 and 10 were excluded from the main analysis due to a high proportion of 'does not apply' responses.
