# Supplementary material for: Development and validation of the Multimorbidity Treatment Burden Questionnaire (MTBQ)
Source: BMJ Open. 2018 Apr 12;8(4):e019413. doi: 10.1136/bmjopen-2017-019413 (PMC5900423; doi:10.1136/bmjopen-2017-019413)
Supplement: Author's manuscript [file bmjopen-2017-019413.draft_revisions.pdf]

BMJ Open is committed to open peer review. As part of this commitment we make the peer review history of every article we publish publicly available.

When an article is published we post the peer reviewers' comments and the authors' responses online. We also post the versions of the paper that were used during peer review. These are the versions that the peer review comments apply to.

The versions of the paper that follow are the versions that were submitted during the peer review process. They are not the versions of record or the final published versions. They should not be cited or distributed as the published version of this manuscript.

BMJ Open is an open access journal and the full, final, typeset and author-corrected version of record of the manuscript is available on our site with no access controls, subscription charges or pay-per-view fees (http://bmjopen.bmj.com).

If you have any questions on BMJ Open's open peer review process please email info.bmjopen@bmj.com

BMJ Open

Development and validation of the Multimorbidity Treatment Burden Questionnaire (MTBQ)

Journal:	*BMJ Open*
Manuscript ID	bmjopen-2017-019413
Article Type:	Research
Date Submitted by the Author:	01-Sep-2017

Complete List of Authors:	Duncan, Polly; University of Bristol, Centre for Academic Primary Care<br>Murphy, Mairead; University of Bristol, Centre for Academic Primary Care<br>Man, Mei-See; University of Bristol School of Social and Community Medicine,<br>Chaplin, Katherine ; University of Bristol School of Social and Community Medicine<br>Gaunt, Daisy; University of Bristol Faculty of Medicine and Dentistry, Bristol Randomised Trials Collaboration & School of Social and Community Medicine<br>Salisbury, Chris; University of Bristol, Academic Unit of Primary Health Care

**Primary Subject Heading**:	General practice / Family practice
Secondary Subject Heading:	Evidence based practice, Geriatric medicine, Health services research, Patient-centred medicine
Keywords:	Treatment burden, Multimorbidity, Patient reported outcome measure, Questionnaire, PRIMARY CARE

SCHOLARONE™ Manuscripts

**Title**	**Development and validation of the Multimorbidity Treatment Burden Questionnaire (MTBQ)**

Authors

Dr Polly Duncan
GP and NIHR In-Practice Fellow
University of Bristol

Dr Mairead Murphy
Senior Research Associate
University of Bristol

Dr Mei-See Man
Trial Manager
University of Bristol

Dr Katherine Chaplin
Senior Research Associate
University of Bristol

Miss Daisy Gaunt
Senior Research Associate in Medical Statistics
University of Bristol

Prof Chris Salisbury
Professor of Primary Health Care
University of Bristol

Corresponding author

Dr Polly Duncan
Room 1.07, Centre for Academic Primary Care,
University of Bristol
Canynge Hall
39 Whatley Road
Bristol, BS8 2PS
01173313903
polly.duncan@bristol.ac.uk

Support National Institute for Health Research funding

Prior presentations Duncan P, 'Development and validation of the Multimorbidity Treatment Burden Questionnaire', oral presentation at the Annual Society for Academic Primary Care Conference, Warwick, UK, 12th July 2017.

Word count Abstract 296 words, main article 3873 words

Numbers of

Tables	5
Figures	0
Appendices	5

For peer review only - http://bmjopen.bmj.com/site/about/guidelines.xhtml

Abstract

Objective: To develop and validate a new scale to assess treatment burden (the effort of looking after one's health) for patients with multimorbidity.

Methods: Design: mixed-methods

Setting: UK primary care

Participants: Content of the Multimorbidity Treatment Burden Questionnaire (MTBQ) was based on a literature review and views from a patient and public involvement group. Face validity was assessed through cognitive interviews. The scale was piloted and the final version was tested in 1546 adults with multimorbidity (mean age 71 years) who took part in the 3D Study, a cluster randomised controlled trial.

For each question, we examined the proportion of missing data and the distribution of responses. Factor analysis, Cronbach's alpha, Spearman's rank correlations and longitudinal regression assessed dimensional structure, internal consistency reliability, construct validity and responsiveness respectively. We assessed interpretability by grouping the global MTBQ scores into zero and tertiles (>0) and comparing participant characteristics across these categories.

Results: Cognitive interviews found good acceptability and content validity. Factor analysis supported a one-factor solution. Cronbach's alpha was 0.83, indicating internal consistency reliability. The MTBQ score had a positive association with a comparator treatment burden scale (Rs 0.58, p<0.0001) and with self-reported disease burden (Rs 0.43, p<0.0001) and a negative association with quality of life (Rs -0.36, p<0.0001) and self-rated health (Rs -0.36, p<0.0001). Female participants, younger participants and participants with mental health conditions were more likely to have high treatment burden scores. Changes in MTBQ score over nine-month follow-up were associated, as expected, with changes in measures of quality of life (EQ-5D-5L) and patient-centred care (PACIC).

Conclusion: The MTBQ is a ten-item measure of treatment burden for patients with multimorbidity that has demonstrated good content validity, construct validity, reliability and responsiveness. It is a useful research tool for assessing the impact of interventions on treatment burden.

Key words: Treatment burden, multimorbidity, patient reported outcome measure, questionnaire, primary care

For peer review only - http://bmjopen.bmj.com/site/about/guidelines.xhtml

Abbreviations:

MTBQ	Multimorbidity treatment burden questionnaire
PROM	Patient reported outcome measure
HCTD	Health Care Task Difficulty questionnaire
TBQ	Treatment Burden Questionnaire
PETS	Patient Experience with Treatment and Self-management questionnaire
MULTIPLes	Multimorbidity Illness Perceptions Scale
EQ-5D-5L	EuroQol five dimensions, five level questionnaire

For peer review only

Article Summary

Strengths and limitations of the study

- A concise simply worded measure based on an evidence-based framework to include all the important aspects of treatment burden
- The measure was comprehensively tested using international standards for validating questionnaires
- Validated in 1546 mostly elderly patients with three or more long-term conditions
- Study participants were recruited into a trial, which may limit generalisability
- High floor effects were found similar to other existing treatment burden questionnaires

For peer review only

For peer review only - http://bmjopen.bmj.com/site/about/guidelines.xhtml

Introduction

Treatment burden is a patient's perception of the effort required to self-manage their medical conditions and the impact that this has on their general wellbeing.[1] This includes complex medication regimens, co-ordinating health care appointments, making lifestyle changes, and self-monitoring.

This is particularly relevant to patients with multimorbidity (having multiple long-term conditions). Associated with the ageing population, multimorbidity has become the norm, affecting over two-thirds of adults attending general practice.[2] Current health policy envisages greater support for patients to self-manage their chronic medical conditions. However, the time and energy this requires of patients can be overwhelming.[3]

In order to understand the impact of treatment burden, and particularly to assess the effects of interventions which might increase or decrease burden, a valid patient reported outcome measure (PROM) is essential. There are four existing PROMs that measure aspects of treatment burden for patients with multimorbidity,[4-8] all of which have important limitations. The 13-question Treatment Burden Questionnaire (TBQ) from Tran et al was originally developed in French, and subsequently a revised 15-question English version was tested.[4 5] Some of the content is healthcare system specific and the wording is relatively complex, perhaps reflecting the fact that the English version was tested in a relatively young and highly educated population of volunteers recruited from an internet forum (mean age 51 years, 78% with college education), not all of whom had multimorbidity.[7] The Patient Experience with Treatment and Self-management (PETS) PROM was recently developed in the United States and includes 48 questions grouped under nine separate domains of treatment burden.[8] Whilst this measure is comprehensive, its length is a limitation. The Multimorbidity Illness Perceptions Scale (MULTIPLes) was developed and validated in elderly patients (mean age 70 years) with multimorbidity (two or more long-term conditions) and includes a six-question Treatment Burden Subscale and a three-question Activity Limitation subscale.[7] This measure is brief but omits important aspects of treatment burden such as having to attend multiple appointments with different health care professionals. Similarly, the 11-question Healthcare Task Difficulty (HCTD) questionnaire was designed to measure only one aspect of treatment burden.[6]

The purpose of this study was to develop and validate a new concise measure of treatment burden for patients with multimorbidity.

For peer review only - http://bmjopen.bmj.com/site/about/guidelines.xhtml

Methods

Study Setting

This questionnaire was developed and validated as part of the 3D Study, a multicentre cluster-randomised control trial that aims to improve the management of patients with multimorbidity within primary care.[9] Participants aged 18 years or older with three or more of the long-term conditions included in the 2014 UK Quality and Outcomes Framework were recruited from 33 general practices in three areas of the UK.

Development of the questionnaire

We identified relevant domains for the PROM by reviewing existing PROMs against a framework of treatment burden which had been developed following qualitative interviews and focus groups.[1] We then sought the views from a Patient and Public Involvement (PPI) group of fourteen patients with multimorbidity formed for the purpose of the 3D Study. We developed a draft questionnaire with 13 questions and undertook two rounds of cognitive interviews with eight PPI group members to improve the face and content validity of the scale (Appendix A).[10] Participants were asked to "think aloud"[10] as they completed the questionnaire commenting on the reasoning behind their ratings; perceived question meaning, the layout, title, introduction and general wording. They also gave their own examples of treatment burden and reflected on whether these would be captured by the questionnaire. Modifications to the questionnaire were made between the two rounds. Following written consent, the interviews were audio-taped and field notes were taken. A debriefing meeting was held with PPI members and final changes to the PROM were made. The final version of the questionnaire was approved by the PPI members.

Recruitment, data collection and measures

Data were collected in two related studies, the cross-sectional 3D pilot study, and the longitudinal main 3D study, a cluster randomised controlled trial. The 13 candidate questions were included in a questionnaire which was named the Multimorbidity Treatment Burden Questionnaire (MTBQ). Socio-demographic information (see Table 1) was collected at baseline in both the pilot and main studies. Details of participant's medical conditions were collected from their family practice computer records. Measures of health-related quality of life (EQ-5D-5L),[11] self-rated health (single question item), self-reported disease burden (Bayliss)[12] and patient-centred care (PACIC)[13] were collected at baseline and nine months in both the pilot and main 3D studies. Following a review of existing measures and discussion with the PPI group, the Health Care Task Difficulty (HCTD)[6] questionnaire was included in the pilot study questionnaire as the best comparator for the MTBQ.

The questionnaire was sent to participants by post. For non-responders, a reminder letter was sent 10-14 days later, and a second reminder phone call was made 10-14 days after this.

For peer review only - http://bmjopen.bmj.com/site/about/guidelines.xhtml

Analysis

Data were analysed using STATA (Version 14). We generated descriptive statistics of participant characteristics for the pilot and main studies. The pilot study data were used to test the pre-specified hypothesis of a positive association between global MTBQ score and HCTD score. The main study data were used for the remainder of the analysis.

We tested the psychometric properties of the questionnaire against the minimum standards set out by the International Society for Quality of Life Research (ISOQOL).[14] The analysis plan and results are described in relation to ISOQOL's six recommended standards.

1. Conceptual and measurement model

1a. Conceptual framework

The domains of treatment burden included in the MTBQ were based on: first, an existing framework for treatment burden which had been derived from qualitative research;[1] second, mapping of existing PROMs against this framework; and third, the views of a group of people with multimorbidity.

1b. Question properties

To assess the properties of the questions, we examined the proportion of missing data and 'does not apply' responses and the distribution of responses. Responses of 'not difficult' or 'does not apply' were scored as zero. Floor and ceiling effects of the MTBQ were compared with the HCTD.[6] Questions with a proportion of 'does not apply' responses greater than 40% were removed and excluded from the analysis.

1c. Dimensionality

To examine the dimensionality of the scale, we performed factor analysis. This is a statistical technique used to reduce a larger number of items into a smaller number of common factors that reflect shared variance.[15] Items which share a lot of variance should have high "loadings" (correlation between the item and the factor), and low uniqueness (variance which is unique to the item, not common to the factor). Loading of at least 0.4 and uniqueness of less than 0.6 are acceptable.[16] The number of factors extracted was decided by a combination of Kaiser's rule (eigenvalues greater than one),[17] the scree plot,[15] and by interpretability of domains.

For peer review only - http://bmjopen.bmj.com/site/about/guidelines.xhtml

2. Reliability

To test internal consistency reliability, we examined the inter-item correlation matrix and calculated Cronbach's alpha, a measure of consistency between the items in a scale. Inter-item correlations between 0.2 and 0.4 were deemed ideal.[18] A Cronbach's alpha of 0.7-0.9 was acceptable.[19]

3. Validity

3a. Content validity

The content validity of the questionnaire was tested iteratively using cognitive interviews (see 'Development of the questionnaire').

3b. Construct validity

Each question was scored as follows: zero (not difficult/ does not apply), one (a little difficult), two (quite difficult), three (very difficult), four (extremely difficult). Participants were excluded if more than 50% of their responses were missing. To calculate a global score, each participant's average score was calculated from the questions answered and multiplied by 25 to give a score from 0-100.

Construct validity was examined by testing five pre-specified hypotheses: first, a positive association between global MTBQ score and global HCTD score;[6] second, a negative association between global MTBQ score and health-related quality of life (EQ-5D-5L);[11] third, a positive association between global MTBQ score and self-reported disease burden score;[12] fourth, a positive association between global MTBQ score and number of self-reported co-morbidities;[12] and fifth, a negative association between global MTBQ and self-rated health (single question item). We applied Spearman's rank correlation to test these hypotheses.

3c. Responsiveness

According to the ISOQOL guidelines, responsiveness to change should be assessed.[19] Due to the non-normal distribution of the global MTBQ score, standard methods to assess responsiveness to change such as calculating an effect size[20] were not possible. We therefore tested the responsiveness of the global MTBQ score by assessing whether changes over time in measures of quality of life (EQ-5D-5L)[11] and patient centred care (PACIC)[13] were inversely associated with changes in MTBQ as anticipated. We used a linear regression model of the standardised change in quality of life (EQ-5D-5L) score between baseline and nine-months on the standardised change in MTBQ between baseline and nine-months. These standardised change scores were calculated at the participant level by dividing the individual difference in nine-month and baseline MTBQ (or EQ-5D-5L) score by the standard deviation of the overall MTBQ (or EQ-5D-5L) change score for all individuals. We then further adjusted this linear regression model in a subsequent analysis by age, gender, number of long-term conditions and individual participant

For peer review only - http://bmjopen.bmj.com/site/about/guidelines.xhtml

deprivation level. All participants that died prior to the nine-month follow-up were given an EQ-5D-5L follow-up score of zero.

We then used the same model for MTBQ specified as above but included the standardised change in PACIC scores between baseline and 9-month follow-up, defined as previously, and subsequently further adjusted this model by the additional covariates as specified.

4. Interpretability of scores

The distribution of global MTBQ scores was examined and compared with the distribution of HCTD[6] scores.

We assessed interpretability of the questionnaire by grouping the global MTBQ scores greater than zero into tertiles. Four categories were generated: no burden (score 0), low burden (score<10), medium burden (10 to 22) and high burden (≥ 22)). Participant characteristics and key outcome variables, including EQ-5D-5L,[11] Bayliss disease burden score[12] and self-rated health, were compared across these four categories (Table 5). To test for associations between treatment burden score category and participant characteristics we performed ordinal logistic regression of MTBQ group (four treatment burden categories) on each participant characteristic. We then further adjusted these ordinal logistic regression models by age, gender, number of co-morbidities, age left full time education and individual deprivation score.

5. Translation

Not applicable.

6. Demands on patient respondents and investigators

The effort required of patient respondents to complete the questionnaire was assessed during the cognitive interviews, and by reviewing the proportion of missing responses. We set out to reduce the demands on investigators by providing clear instructions on how to calculate a global MTBQ score, including handling of missing data, and how to report and interpret these scores.

Ethical approval and data sharing

The 3D study was approved by South-West (Frenchay) NHS Research Ethics Committee (14/SW/0011). Trial registration number: ISRCTN06180958. Data will be available from the University of Bristol Research Data Storage Facility after the main results of the 3D trial have been published in 2018.

For peer review only - http://bmjopen.bmj.com/site/about/guidelines.xhtml

For peer review only

For peer review only - http://bmjopen.bmj.com/site/about/guidelines.xhtml

Results

Participant Characteristics

143 adults participated in the pilot study. In the main 3D study, 1546 completed the main study baseline questionnaire of which 1524 (99%) completed at least half of the baseline MTBQ questions and 1299 (84%) completed at least half of the follow-up MBTQ questionnaire after 9 months. The participants were mostly elderly (mean age 71 years for the main study), fully retired from work and had left school aged 16 years or younger (Table 1).

INSERT TABLE 1

1. Conceptual and measurement model

1a. Conceptual framework

In line with the framework developed by Eton et al,[1] and following the literature review and patient consultation, the 13 questions in the MTBQ encompass three major themes. These were the work required to look after one's health (e.g. self-monitoring, making lifestyle changes); tools and strategies patients use to reduce their treatment burden (e.g. organising medication); and factors that increase burden (e.g. poor continuity of care).

1b. Question properties

The proportion of missing data for each question was between 1% and 3% (see Table 2). Questions 3, 9 and 10 had a high proportion of 'does not apply' responses (Table 2). These questions were excluded from the main analysis. Since the questions might apply to other populations (e.g. question three about the cost of treatment is likely to be relevant to populations where patients pay for their health care), we repeated Cronbach's alpha including these questions in the various combinations (Appendix B). These extra questions may be considered as optional depending on the study population. Responses were positively skewed and a floor effect was found for some questions. However, the MTBQ had fewer floor effects than the comparator HCTD (Appendix C).

The Global MTBQ scores were also skewed with 26% of pilot study participants and 22% of main study participants scoring zero (Appendix D). Again, the HCTD had greater floor effects, with 54% of participants having a global score of zero.

INSERT TABLE 2

1c. Dimensionality

Both Kaiser's "eigenvalue greater than one" rule and Cattell's scree plot criterion suggested a one factor solution and this explained 93% of the common variance.

For peer review only - http://bmjopen.bmj.com/site/about/guidelines.xhtml

Loadings on this factor were uniformly greater than 0.4. The factor solution had high uniqueness for some items. This can sometimes indicate that the item is not strongly related to others,[15] but because of the important content of these variables (e.g. lifestyle changes, collecting medication), we chose to include them.

2. Reliability

Questions 1 and 2 have a high inter-item correlation of 0.69 and questions 6 and 7 have an inter-item correlation of 0.62 (Appendix E). Almost all of the other inter-item correlations were in the ideal range of 0.2 to 0.4. Cronbach's Alpha was 0.83 indicating a high level of internal reliability. Including the optional questions (questions 3, 9 and 10) in various combinations, Cronbach's Alpha ranged from 0.82 to 0.84, again demonstrating good internal consistency (see Appendix B).

3. Validity

3a. Face and Content validity

Participants from the PPI group commented that the wording was clear and easy to understand. All but one of the participants felt that the important areas of treatment burden were covered by the questionnaire.

3b. Construct validity

As predicted, the global MTBQ score had a positive association with the comparator HCTD scale[6] (r_s 0.58, p<0.0001), the Bayliss disease burden scale[12] (r_s 0.43, p<0.0001) and the number of self-reported co-morbidities (r_s 0.32, p<0.0001); and a negative association with the quality of life scale[11] (r_s -0.36, p<0.0001) and self-rated health (r_s -0.36, p<0.0001) (Table 3). This provides good evidence for construct validity of the scale.

INSERT TABLE 3

3c. Responsiveness

Regression analysis found that for every 1 standard deviation (i.e. 0.17) increase in EQ-5D-5L score[11] between baseline and nine-month follow-up, MTBQ score at follow-up was reduced by 1.7 (regression coefficient -0.14 multiplied by a standard deviation change in MTBQ score of 11.9, (95% CI for regression coefficient -0.19 to -0.08), p value < 0.0001) (see Table 4). This association was also seen after further adjusting the model for the specified covariates (regression coefficient -0.14 (95% CI -0.20 to -0.08), p value <0.0001).

The equivalent model for PACIC score[13] showed that for every 1 standard deviation (i.e. 0.86) increase in PACIC score between baseline and nine-month follow-up,

For peer review only - http://bmjopen.bmj.com/site/about/guidelines.xhtml

MTBQ at follow-up was reduced by 1.9 (regression coefficient -0.16 multiplied by a standard deviation change in MTBQ score of 11.9, (95% CI for regression coefficient -0.22 to -0.10), p value < 0.0001). A similar decrease was also seen after further adjusting the model for the specified covariates (regression coefficient -0.17, (95% CI -0.23 to -0.11), p value< 0.0001).

INSERT TABLE 4

4. Interpretability of scores

Comparing participants across the four treatment burden groups (no burden, low burden, medium burden and high burden) female participants; younger participants; those with a greater number of long-term conditions; participants with depression, dementia and severe mental health problems listed on their GP records; and participants with worse EQ-5D-5L scores[11], high disease burden scores[12] and poor self-rated health were more likely to have a high treatment burden score, after adjusting for age, gender, number of co-morbidities, age left full time education and individual deprivation level (see Table 5).

INSERT TABLE 5

5. Translation

Not applicable.

6. Demands on patient respondents and investigators

We have reduced the effort required from patient responders to complete the questionnaire by developing a short ten-item questionnaire with simple wording, fitting on one side of A4 paper in size 14 font. Participants who took part in the cognitive interviews found this relatively simple to complete and the proportion of missing data was between 1% and 3%. To reduce demands on investigators, we have provided clear instructions on calculating, reporting and interpreting global MTBQ scores.

For peer review only - http://bmjopen.bmj.com/site/about/guidelines.xhtml

Discussion

In this study, we have developed and validated a ten-item questionnaire, named the Multimorbidity Treatment Burden Questionnaire (MTBQ). The psychometric properties of the questionnaire meet the minimum standards for a PROM set out by ISOQOL,[19] demonstrating good content validity, internal reliability consistency, construct validity and responsiveness. Three additional questions, including one question about the cost of treatment, had a high proportion of 'does not apply' responses in this study population and were omitted from the main analysis. However, these questions may be relevant to other populations (e.g. countries where patients pay for prescriptions and health care) and the scale remained internally consistent and reliable when they were included, so they may be considered as optional.

We found that younger patients were more likely to report high treatment burden scores and, interestingly, the Tran TBQ found the same phenomenon.[4] There are several possible explanations for this. First, treatment burden may impact more on younger patients because they must juggle their appointments or complex medication regimens alongside having to work or look after dependants. Second, younger patients may have different expectations of how looking after one's health might impact on their lives and, hence, suffer from a greater perceived treatment burden. As expected, we found that patients with mental health conditions including depression and dementia were more likely to have high treatment burden scores. Previous studies have reported similar findings.[6 7] High treatment burden was also associated with having a greater number of long-term conditions. No individual physical condition was found to be associated with high treatment burden. This result differs from both the TBQ study, which found an association between treatment burden and diabetes, and the HCTD study, which found an association between treatment burden and stroke, congestive heart failure and falls.[4 6] As expected, participants with low quality of life (EQ-5D-5L)[11] score, high disease burden score[12] and poor self-rated health were more likely to have high treatment burden. We also found that female participants were more likely to report high treatment burden compared to males. This has not been reported elsewhere.

A key strength of this study is that the MTBQ has been validated in a large sample of participants for whom it is intended – elderly multimorbid patients with a mean age of 71 years and three or more long-term conditions. In comparison, the English version of the Tran Treatment Burden Questionnaire was validated in a younger computer-literate population with a mean age of 51 years.[4 5] The MTBQ had good face validity, was found to be user friendly and fits on a single page of A4 paper in size 14 font. All aspects of treatment burden identified in a comprehensive evidence based framework are included in the questionnaire. In comparison, the most comprehensive existing questionnaire, the PETS questionnaire,[8] includes 48 questions and is time consuming to complete, and several of the other existing questionnaires focus on only some aspects of treatment burden.[6 7] Preliminary assessment of responsiveness found that, as expected, a positive change in both quality of life (EQ-5D-5L)[11] score and patient centred care (PACIC)[13] score between baseline and nine-month follow-up was associated with a reduction in treatment burden (MTBQ) score. Of the other relevant PROMs, only the HCTD has been assessed for responsiveness[6] but the HCTD addresses fewer topics and has a

For peer review only - http://bmjopen.bmj.com/site/about/guidelines.xhtml

narrower range of response options, possibly contributing to its greater problems with skewness and floor effects.

The participants of this study were recruited into a trial, which creates potential for selection bias and may limit generalisability. However, the trial participants had similar characteristics to those invited but declining participation in respect of age, gender, number and type of long-term conditions (data will be shown in papers reporting the 3D trial results). Almost all the participants of this study were white British and further work is planned to validate the questionnaire in other populations. We found high floor effects with 22% of participants scoring a global MTBQ score of zero. All of the other treatment burden measures also show similarly high floor effects.[4-8] One explanation for this is a 'response shift', whereby patients adapt their everyday life so that looking after their health conditions becomes more acceptable to them over time and causes less perceived burden.[21] The implications of positively skewed treatment burden scores and high floor effects are: first, this can make it difficult to detect change (i.e. it is not possible to improve from a treatment burden score of zero); and second, mean treatment burden scores should be interpreted with caution. Preliminary analysis of responsiveness, however, has shown that changes in MTBQ score correlate as expected with changes in quality of life (EQ-5D-5L)[11] score and patient centred care (PACIC)[13], over time. We recommend that, due to the skewness of global MTBQ scores, researchers should report the median and interquartile range rather than the mean and standard deviation and report the proportion of patients with high, medium, low or no treatment burden (MTBQ scores ≥22, 10-22, <10 and 0 respectively).

The MTBQ scale is a concise measure of treatment burden for patients with multimorbidity that has demonstrated good content validity, construct validity, internal consistency reliability and responsiveness. It is a useful research tool for assessing the impact of interventions on treatment burden for patients with multimorbidity. We anticipate the scale being used alongside other measures, such as disease burden, and that findings from the two measures will be related. The MTBQ could also be used in clinical practice to highlight problem areas, such as difficulties the patient may have with their medication or with making recommended lifestyle changes.

For peer review only

Acknowledgements

Appreciation is extended to members of the Patient and Public Involvement group, known as the Patient Involvement in Primary Care Research (PIP-CaRe) group, who took part in the cognitive interviews. The PIP-CaRe group was formed for the purpose of the 3D Study and consists of people with two or more long-term conditions. We also thank all other members of the 3D research team and Professor Boyd for permission to use the Healthcare Task Difficulty questionnaire.

Conflict of interest statement

This work was funded by the National Institute for Health Research Health Services and Delivery Research Programme (project number 12/130/15). The views and opinions expressed therein are those of the authors and do not necessarily reflect those of the HS&DR Programme, NIHR, NHS or the Department of Health.

For peer review only

Author statement

PD, MSM, and CS were responsible for study concept and design. PD, MM, DG and KC were involved in data extraction and analysis. PD drafted the manuscript. All authors critically reviewed the manuscript and approved the final version. ***All authors*** also had full access to all of the data (including statistical reports and tables) in the study and can take responsibility for the integrity of the data and the accuracy of the data analysis. ***PD*** is the guarantor.

Dr Polly Duncan	PD led this project under supervision from Professor Chris Salisbury. She designed the study, undertook a literature review, developed the questionnaire, conducted and analysed the cognitive interviews, convened meetings with the patient and public involvement group, analysed the results and drafted the paper.
Dr Mairead Murphy	MM provided methodological expertise in assessing the psychometric properties of this new patient reported outcome measure, including the approach to analysis and interpretation of the results. She critically appraised the paper and has approved the final version.
Dr Mei-See Man	MSM provided methodological and practical expertise, and obtained ethical and governance approvals for this study. She critically appraised the paper and has approved the final version.
Dr Katherine Chaplin	KC acquired and cleaned the original data and produced the database used for analysis. She critically appraised the paper

For peer review only - http://bmjopen.bmj.com/site/about/guidelines.xhtml

	and has approved the final version.
Miss Daisy Gaunt	DG provided methodological expertise in analysing the responsiveness of the MTBQ and the interpretation of these results. She critically appraised the paper and has approved the final version.
Prof Chris Salisbury	CS was Chief Investigator on the 3D study which formed the basis for this paper, and supervised PD in developing this questionnaire. He contributed to study design, analysis and interpretation. He critically appraised the paper and has approved the final version.

For peer review only

For peer review only - http://bmjopen.bmj.com/site/about/guidelines.xhtml

References

1. Eton DT, Ramalho de Oliveira D, Egginton JS, et al. Building a measurement framework of burden of treatment in complex patients with chronic conditions: a qualitative study. *Patient Relat Outcome Meas* 2012;3:39-49. doi: 10.2147/prom.s34681 [published Online First: 2012/11/28]

2. Salisbury C, Johnson L, Purdy S, et al. Epidemiology and impact of multimorbidity in primary care: a retrospective cohort study. *Br J Gen Pract* 2011;61(582):e12-21.

3. May CR, Eton DT, Boehmer K, et al. Rethinking the patient: using Burden of Treatment Theory to understand the changing dynamics of illness. *BMC Health Serv Res* 2014;14:281. doi: 10.1186/1472-6963-14-281 [published Online First: 2014/06/28]

4. Tran VT, Montori VM, Eton DT, et al. Development and description of measurement properties of an instrument to assess treatment burden among patients with multiple chronic conditions. *BMC Medicine* 2012;10:68. doi: http://dx.doi.org/10.1186/1741-7015-10-68

5. Tran VT, Harrington M, Montori VM, et al. Adaptation and validation of the Treatment Burden Questionnaire (TBQ) in English using an internet platform. *BMC Med* 2014;12:109. doi: 10.1186/1741-7015-12-109 [published Online First: 2014/07/06]

6. Boyd CM, Wolff JL, Giovannetti E, et al. Healthcare task difficulty among older adults with multimorbidity. *Med Care* 2014;52 Suppl 3:S118-25. doi: 10.1097/MLR.0b013e3182a977da [published Online First: 2014/02/25]

7. Gibbons CJ, Kenning C, Coventry PA, et al. Development of a multimorbidity illness perceptions scale (MULTIPleS). *PLoS ONE [Electronic Resource]* 2013;8(12):e81852. doi: http://dx.doi.org/10.1371/journal.pone.0081852

8. Eton DT, Yost KJ, Lai JS, et al. Development and validation of the Patient Experience with Treatment and Self-management (PETS): a patient-reported measure of treatment burden. *Qual Life Res* 2016 doi: 10.1007/s11136-016-1397-0 [published Online First: 2016/08/26]

9. Man MS, Chaplin K, Mann C, et al. Improving the management of multimorbidity in general practice: protocol of a cluster randomised controlled trial (The 3D Study). *Bmj Open* 2016;6(4):e011261. doi: 10.1136/bmjopen-2016-011261

10. Willis GB, Caspar RA, Lessler JT. Cognitive Interviewing: a "How To" guide. Meeting of the American Statistical Association, 1999.

11. EuroQol--a new facility for the measurement of health-related quality of life. *Health Policy* 1990;16(3):199-208. [published Online First: 1990/11/05]

For peer review only - http://bmjopen.bmj.com/site/about/guidelines.xhtml

12. Bayliss EA, Ellis JL, Steiner JF. Seniors' self-reported multimorbidity captured biopsychosocial factors not incorporated into two other data-based morbidity measures. *J Clin Epidemiol* 2009;62(5):550-7.e1. doi: 10.1016/j.jclinepi.2008.05.002 [published Online First: 2008/09/02]

13. Glasgow RE, Wagner EH, Schaefer J, et al. Development and validation of the Patient Assessment of Chronic Illness Care (PACIC). *Med Care* 2005;43(5):436-44.

14. Reeve BB, Wyrwich KW, Wu AW, et al. ISOQOL recommends minimum standards for patient-reported outcome measures used in patient-centered outcomes and comparative effectiveness research. *Qual Life Res* 2013;22(8):1889-905. doi: 10.1007/s11136-012-0344-y [published Online First: 2013/01/05]

15. Tabachnick BG, Fidell LS, dawsonera. Using multivariate statistics. 6th ed. Harlow: Pearson, 2014:1056.

16. Factor Analysis http://www.stata.com/manuals13/mvfactor.pdf2015 [accessed 22.08.2017.

17. Kaiser HF. The application of electronic computers to factor analysis. . *Educational and Psychological Measurement* 1960;20:141-51.

18. Piedmont RL. Inter-item Correlations. In: Michalos AC, ed. Encyclopedia of Quality of Life and Well-Being Research. Dordrecht: Springer Netherlands 2014:3303-04.

19. Reeve BB, Wyrwich KW, Wu AW, et al. ISOQOL recommends minimum standards for patient-reported outcome measures used in patient-centered outcomes and comparative effectiveness research. *Quality of Life Research* 2013;22(8):1889-905. doi: 10.1007/s11136-012-0344-y

20. Streiner DL, Norman GR. Health Measurement Scales a practical guide to their development and use. Fourth edition ed: Oxford University Press 2008.

21. Sprangers MAG, Schwartz CE. Integrating response shift into health-related quality of life research: a theoretical model. *Social Science & Medicine* 1999;48(11):1507-15. doi: http://dx.doi.org/10.1016/S0277-9536(99)00045-3

For peer review only

Table 1: Participant Characteristics (main study N = 1546, pilot study N = 143)

		Pilot study n/N* (%)	Main study n/N* (%)
Mean age (SD)		74 (10)	71 (12)
Age (years)	≤ 50	3 (2)	79 (5)
	51-60	9 (6)	196 (13)
	61-70	27 (19)	420 (27)
	71-80	67 (47)	510 (33)
	81-90	33 (23)	315 (20)
	≥ 90	4 (3)	26 (2)
Gender	Male	65 (45)	763 (49)
Number of comorbidities	Three	109 (76)	1234 (80)
	Four	23 (16)	277 (18)
	Five	10 (7)	31 (2)
	Six	1 (<1)	4 (<1)
Comorbidities*	Cardiovascular disease	138 (97)	1445 (7)
	Stroke/TIA	35 (25)	527 (34)
	Diabetes	63 (44)	811 (52)
	Chronic kidney disease	83 (58)	464 (30)
	COPD or asthma	58 (41)	770 (50)
	Epilepsy	6 (4)	76 (5)
	Atrial fibrillation	46 (32)	529 (34)
	Severe mental health problems [a]	2 (1)	66 (4)
	Depression	26 (18)	560 (36)
	Dementia	6 (4)	60 (4)
	Learning disability	3 (2)	14 (1)
	Rheumatoid arthritis	9 (6)	103 (7)
	Heart failure	14 (10)	157 (10)
Ethnicity	White British	135/136 (99)	1502/1519 (99)
Age left full-time education (years)	≤ 14	22 (15)	154/1541 (10)
	15 or 16	74 (52)	907/1541 (59)
	17 or 18	25 (17)	222/1541 (14)
	≥ 19	22 (15)	258/1541 (17)
Employment status	Fully retired from work	113/139 (81)	1044/1501 (70)
Mean deprivation score [b] (SD, N)	England	10.7 (7.7, 143)	15 (13, 1078)
	Scotland		26 (17, 467)
**Outcome measures**			
Mean HCTD score[c] (SD, N)		1.14 (1.7, 143)	
Mean self-reported disease burden score[d] (SD, N)			19 (12.4, 1458)
Mean number of self-reported conditions[e] (SD, N)			8 (3.2, 1543)
Mean quality of life score[f] (SD, N)			0.6 (0.3, 1542)
Mean self-rated health score[g] (SD, N)			2 (0.8, 1523)
Mean patient centred health score[h] (SD, N)			2.5 (1.0, 1232)

* For characteristics where there is no missing data n is shown, for characteristics with missing data n/N is shown. [a]Including schizophrenia and psychotic illness. [b]Individual Index of Multiple Deprivation (IMD) score, 2010, for England, and Scottish Index of Multiple Deprivation (SIMD) score, 2010, for Scotland, for both a higher score correlates with greater deprivation [c] Calculation of global HCTD score: sum of scores where each question was scored 0 (no difficulty), 1 (some difficulty), or 2 (a lot of difficulty). Minimum score 0, maximum score 16. Missing data was scored 0 (not difficult) as suggested by the HCTD authors[6] [d]Sum of the weighted scores (each scored 1-5) from the Bayliss scale.[12] Responses were excluded if participants ticked that they had a condition but did not score how much the condition limited their daily activity of if they gave a score without ticking that they had the condition. [e]Number of self-reported conditions from the Bayliss scale. [f]EQ-5D-5L score.[11] [g]Single question. 'In general, would you say your health is poor (1), fair (2), good (3), very good (4) or excellent (5)?' [h]PACIC score[13]

For peer review only

For peer review only - http://bmjopen.bmj.com/site/about/guidelines.xhtml

Table 2: Responses to the Multimorbidity Treatment Burden Questionnaire (main study baseline data, N = 1546)

Please tell us how much difficulty you have with the following:	N	Not difficult n (n/N %)	A little difficult n (n/N %)	Quite difficult n (n/N %)	Very difficult n (n/N %)	Extremely difficult n (n/N %)	Does not apply n (n/N %)
1. Taking lots of medications	1518	1083 (71)	257 (17)	104 (7)	25 (2)	20 (1)	29 (2)
2. Remembering how and when to take medication	1519	1123 (74)	271 (18)	60 (4)	21 (1)	23 (2)	21 (1)
3. Paying for prescriptions, over the counter medication or equipment	1506	312 (21)	17 (1)	18 (1)	4 (<1)	8 (1)	1147 (76)
4. Collecting prescription medication	1514	951 (63)	221 (15)	63 (4)	22 (1)	28 (2)	229 (15)
5. Monitoring your medical conditions (eg. checking your blood pressure or blood sugar, monitoring your symptoms etc)	1513	748 (49)	191 (13)	111 (7)	35 (2)	37 (2)	391 (26)
6. Arranging appointments with health professionals	1507	765 (51)	321 (21)	210 (14)	81 (5)	66 (4)	64 (4)
7. Seeing lots of different health professionals	1506	642 (43)	309 (21)	192 (13)	85 (6)	68 (5)	210 (14)
8. Attending appointments with health professionals (eg. getting time off work, arranging transport etc)	1512	771 (51)	187 (12)	107 (7)	51 (3)	44 (3)	352 (23)
9. Getting health care in the evenings and at weekends	1496	311 (21)	156 (10)	184 (12)	106 (7)	121 (8)	618 (41)
10. Getting help from community services (eg. physiotherapy, district nurses etc)	1500	393 (26)	138 (9)	111 (7)	51 (3)	54 (4)	753 (50)
11. Obtaining clear and up-to-date information about your condition	1499	794 (53)	263 (18)	179 (12)	62 (4)	47 (3)	154 (10)
12. Making recommended lifestyle changes (eg. diet and exercise)	1505	534 (35)	327 (21)	203 (13)	112 (7)	75 (5)	254 (17)
13. Having to rely on help from family and friends	1509	675 (45)	213 (14)	140 (9)	59 (4)	70 (5)	352 (23)

Please note: Questions 3, 9 and 10 were excluded from the main analysis due to a high proportion of 'does not apply' responses. They are shown in italics. As they may be relevant to other populations, they can be considered as optional.

For peer review only - http://bmjopen.bmj.com/site/about/guidelines.xhtml

Table 3: Association between global MTBQ score and global HCTD score, self-reported disease burden score, quality of life score, number of self-reported conditions and self-rated health at baseline

Variable	N	Spearman's rank correlations (Rs)	P value
Global HCTD score[a]	141	0.58	< 0.0001
Self-reported disease burden score[b]	1443	0.42	< 0.0001
Number of self-reported conditions[c]	1523	0.31	< 0.0001
Quality of life score[d]	1520	-0.36	< 0.0001
Self-rated health[e]	1503	-0.36	< 0.0001

[a] Calculation of global HCTD score: sum of scores where each question was scored 0 (no difficulty), 1 (some difficulty), or 2 (a lot of difficulty). Minimum score 0, maximum score 16. Missing data was scored 0 (not difficult) as suggested by the HCTD authors[6] [b]Sum of the weighted scores (each scored 1-5) from the Bayliss scale.[12] Responses were excluded if participants ticked that they had a condition but did not score how much the condition limited their daily activity of if they gave a score without ticking that they had the condition. [c]Number of self-reported conditions from the Bayliss scale. [d]EQ-5D-5L score.[11] [e]Single question. 'In general, would you say your health is poor (1), fair (2), good (3), very good (4) or excellent (5)?'

For peer review only

For peer review only - http://bmjopen.bmj.com/site/about/guidelines.xhtml

Table 4: Association between global MTBQ score and (i) quality of life (EQ-5D-5L)[11] score; and (ii) Patient Assessment of Chronic Illness Care (PACIC)[13] score. Results from linear regression model of standardised change

Outcome	N[a]	Linear regression coefficient of MTBQ standardised change score (95% CI)	P value	N	Adjusted[b] linear regression coefficient of MTBQ standardised change score (95% CI)	P value
**EQ-5D-5L standardised change score**	1270	-0.14 (-0.19 to -0.08)	< 0.0001	1239	-0.14 (-0.20 to -0.08)	< 0.0001
**PACIC standardised change score**	930	-0.16 (-0.22 to -0.10)	< 0.0001	914	-0.17 (-0.23 to -0.11)	< 0.0001
**Outcome**	**N[c]**	**Standard deviation change in score between baseline and nine-month follow-up**				
**EQ-5D-5L**	1344	0.17				
**PACIC**	946	0.86				
**MTBQ**	1285	11.9				

[a] This analysis included participants who completed the outcome questionnaire (EQ-5D-5L or PACIC) and the MTBQ questionnaire at baseline and nine-month follow-up. [b] Linear regression model further adjusted for age, gender, number of co-morbidities, age left full time education and individual deprivation score. [c] This analysis included participants who completed the outcome questionnaire (EQ-5D-5L, PACIC or MTBQ) at baseline and nine-month follow-up

For peer review only - http://bmjopen.bmj.com/site/about/guidelines.xhtml

Table 5: Characteristics by categories of treatment burden (main study baseline data)

		N	None (0)	Low (<10)	Medium (10-22)	High (≥ 22)	Unadjusted OR*	Adjusted OR**	P value
**Participants**		1524	308	385	425	406			
**Age (mean)**		1524	74	73	71	66	**0.96 (0.95 to 0.97)**	**0.96 (0.95 to 0.97)**	**<0.0001**
**Gender [n, (%)]**	**Male**	651	168 (22)	208 (28)	193 (26)	182 (24)	**0.74 (0.62 to 0.88)**	**0.73 (0.60 to 0.87)**	**0.001**
**Number of long-term conditions [n,(%)]**	**Three**	1217	246 (20)	323 (27)	335 (28)	313 (26)			
	**Four or more**	307	62 (20)	62 (20)	90 (29)	93 (30)	1.21 (0.97 to 1.52)	**1.38 (1.09 to 1.74)**	**0.007**
**Long-term conditions [n, (%)]**	**Cardiovascular disease**	1423	294 (21)	367 (26)	389 (27)	373 (26)	**0.62 (0.44 to 0.91)**	0.79 (0.54 to 1.14)	0.208
	**Stroke/TIA**	517	127 (25)	140 (27)	135 (26)	115 (22)	**0.69 (0.57 to 0.83)**	0.82 (0.67 to 1.01)	0.059
	**Diabetes**	800	158 (20)	200 (25)	211 (26)	231 (29)	1.13 (0.94 to 1.35)	1.04 (0.87 to 1.26)	0.633
	**Chronic kidney disease**	454	101 (22)	121 (27)	115 (25)	117 (26)	0.86 (0.71 to 1.05)	1.10 (0.89 to 1.36)	0.356
	**COPD or asthma**	758	148 (20)	185 (24)	222 (29)	203 (27)	1.08 (0.90 to 1.29)	0.91 (0.75 to 1.10)	0.326
	**Epilepsy**	76	14 (18)	21 (28)	24 (32)	17 (22)	0.94 (0.63 to 1.41)	0.76 (0.50 to 1.17)	0.216
	**Atrial fibrillation**	524	119 (23)	155 (30)	142 (27)	108 (21)	0.68 (0.56 to 0.82)	0.91 (0.74 to 1.12)	0.369
	**Severe mental health problems**[a]	66	7 (11)	10 (15)	17 (26)	32 (48)	**2.61 (1.64 to 4.15)**	**1.75 (1.08 to 2.82)**	**0.022**
	**Depression**	553	85 (15)	105 (19)	169 (31)	194 (35)	**1.92 (1.59 to 2.32)**	**1.43 (1.16 to 1.77)**	**0.001**
	**Dementia**	58	14 (24)	10 (17)	12 (21)	22 (38)	1.27 (0.78 to 2.11)	**2.26 (1.34 to 3.81)**	**0.002**
	**Learning disability**	14	2 (14)	2 (14)	6 (43)	4 (29)	1.47 (0.59 to 3.69)	1.07 (0.36 to 3.21)	0.907
	**Rheumatoid arthritis**	102	15 (15)	18 (18)	40 (39)	29 (28)	1.41 (0.99 to 2.01)	1.28 (0.88 to 1.82)	0.202
	**Heart failure**	154	36 (23)	41 (27)	38 (25)	39 (25)	0.85 (0.63 to 1.14)	1.06 (0.77 to 1.44)	0.340
**Age left full-time education [n, (%)]**	**≤16 years**	681	164 (24)	172 (25)	177 (26)	168 (25)	1.00 (0.99 to 1.01)	1.01 (0.99 to 1.02)	0.450
**Deprivation score (mean)***	**England**	1078	15	15	15	16	1.01 (1.00 to 1.01)	1.00 (0.99 to 1.01)	0.904
	**Scotland**	467	26	26	24	24	1.00 (0.99 to 1.01)	**0.99 (0.99 to 1.00)**	**0.032**
**EQ-5D-5L[11] (mean)**		1520	0.67	0.63	0.56	0.42	**0.11 (0.08 to 0.16)**	**0.09 (0.06 to 0.12)**	**<0.0001**
**Disease-burden score[12] (mean)**		1443	12.8	15.7	19.0	26.1	**1.06 (1.06 to 1.08)**	**1.07 (1.07 to 1.09)**	**<0.0001**
**Self-rated health [n, (%)]**	**Poor**	315	36 (11)	42 (13)	75 (24)	162 (51)			
	**Fair**	674	112 (17)	168 (25)	216 (32)	178 (26)	**0.39 (0.30 to 0.50)**	**0.41 (0.31 to 0.53)**	**<0.0001**
	**Good**	422	111 (26)	138 (33)	116 (27)	57 (14)	**0.20 (0.15 to 0.26)**	**0.19 (0.14 to 0.26)**	**<0.0001**
	**Very good**	87	40 (46)	28 (32)	16 (18)	3 (3)	**0.08 (0.05 to 0.13)**	**0.08 (0.05 to 0.12)**	**<0.0001**

For peer review only

For peer review only - http://bmjopen.bmj.com/site/about/guidelines.xhtml

	**Excellent**	5	3 (60)	2 (40)	0	0	**0.04 (0.01 to 0.23)**	**0.03 (0.00 to 0.16)**	**<0.0001**

*ordinal logistic regression comparing no burden (0), low burden (<10), medium burden (10-22) and high burden (≥22) ** ordinal logistic regression comparing no burden (0), low burden (<10), medium burden (10-22) and high burden (≥22), adjusted for age, gender, number of co-morbidities, age left full time education and individual deprivation score *** Individual Index of Multiple Deprivation (IMD) score, 2010, for England, and Scottish Index of Multiple Deprivation (SIMD) score, 2010, for Scotland, for both a higher score correlates with greater deprivation. [a]Including schizophrenia and psychotic illesss

For peer review only

Appendix A: Characteristics of the participants who took part in the cognitive interviews (n=8)

Characteristic	Value
Mean age years (SD, min, max)	55.5 (14.1, 30, 78)
Male	2 (25%)
White British ethnicity	8 (100%)
Mean number of self-reported long-term conditions (SD, min, max)	2.1 (1.5, 1, 5)

For peer review only

Appendix B: Cronbach's alpha including the optional questions (questions 3, 9 and 10) in the various combinations

	Optional questions						
	**3**	**9**	**10**	**3, 9, 10**	**3, 9**	**3, 10**	**9, 10**
**Cronbach's alpha**	0.82	0.83	0.83	0.84	0.83	0.83	0.84

Optional questions: Please tell us how much difficulty you have with the following:

Question 3. Paying for prescriptions, over the counter medication or equipment
Question 9. Getting health care in the evenings and at weekends
Question 10. Getting help from community services (e.g. physiotherapy, district nurses etc)

For peer review only

Appendix C: A comparison of the floor effects and missing data of the MTBQ and the HCTD (pilot study data)

MTBQ Question	Floor effect[a] %	Missing data %	HCTD question with a similar latent construct	Floor effect [b] %	Missing data (%)
**1.** Taking lots of medications	78	1	3. Difficulty taking medications	95	1
**2.** Remembering how and when to take medication	80	1	2. Difficulty planning medication schedule	94	3
3. *Paying for prescriptions, over the counter medication or equipment*	94	4	5. Difficulty paying prescription charges	78	19
**4.** Collecting prescription medication	83	2	1. Difficulty obtaining medications	87	1
**5.** Monitoring your medical conditions (eg. checking your blood pressure or blood sugar, monitoring your symptoms etc)	83	2	No question to compare with		
**6.** Arranging appointments with health professionals	59	3	6. Difficulty scheduling medical appointment	69	4
**7.** Seeing lots of different health professionals	62	2	No question to compare with		
**8.** Attending appointments with health professionals (eg. getting time off work, arranging transport etc)	74	1	7. Difficulty arranging transportation	76	6
9. *Getting health care in the evenings and at weekends*	70	3	No question to compare with		
10. *Getting help from community services (eg. physiotherapy, district nurses etc)*	83	2	No question to compare with		
**11.** Obtaining clear and up-to-date information about your condition	70	2	8. Difficulty getting information	74	4
**12.** Making recommended lifestyle changes (eg. diet and exercise)	57	3	No question to compare with		
**13.** Having to rely on help from family and friends	69	1	No question to compare with		

[a] proportion (%) of 'does not apply' or 'not difficult' responses
[b] proportion (%) 'not difficult' responses
Please note: Questions 3, 9 and 10 were excluded from the main analysis due to a high proportion of 'does not apply' responses. They are shown in italics. As they may be relevant to other populations, they can be considered as optional

For peer review only

Appendix D: Histogram of global MTBQ scores and global HCTD scores (pilot study and main study)

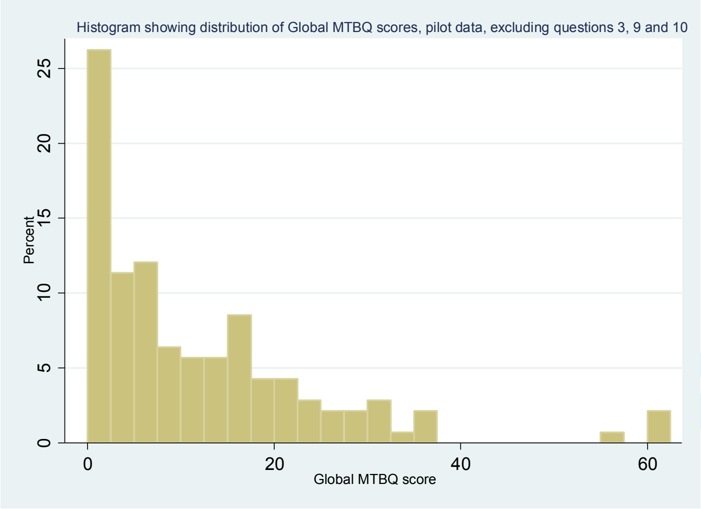

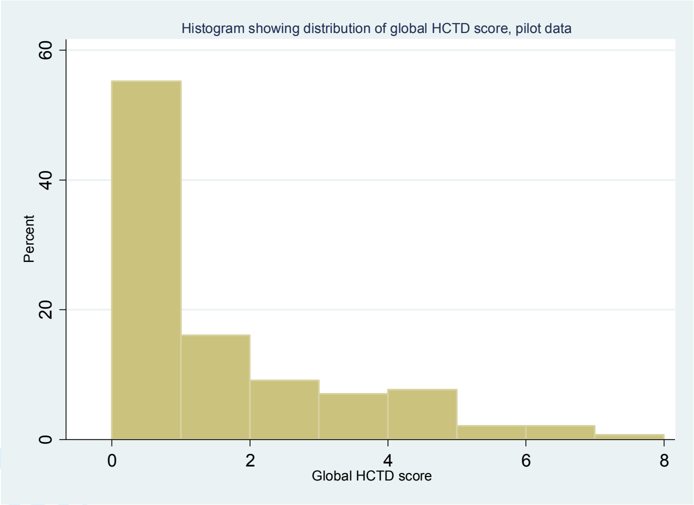

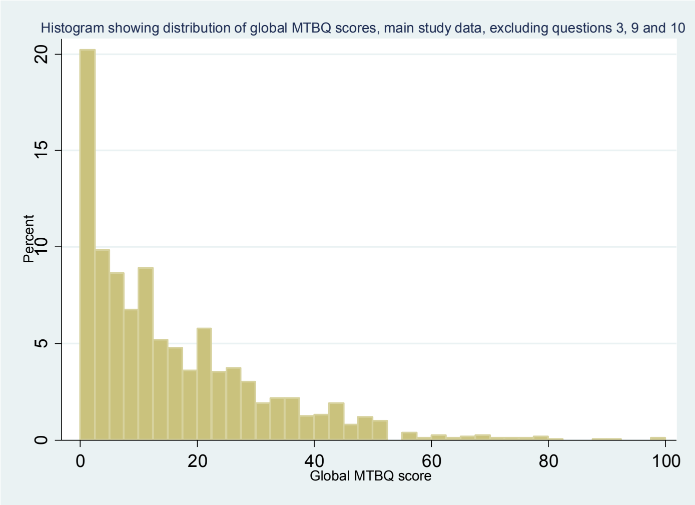

For peer review only - http://bmjopen.bmj.com/site/about/guidelines.xhtml

Appendix E: Inter-item correlation coefficient and Cronbach's Alpha (main study data, excluding questions 3, 9 and 10)

Cronbach's alpha = 0.83

Question:	1	2	4	5	6	7	8	11	12	13
**1**	1.00									
**2**	0.69	1.00								
**4**	0.30	0.26	1.00							
**5**	0.35	0.33	0.31	1.00						
**6**	0.26	0.23	0.28	0.31	1.00					
**7**	0.34	0.29	0.29	0.38	0.62	1.00				
**8**	0.32	0.32	0.40	0.33	0.37	0.44	1.00			
**11**	0.24	0.19	0.27	0.27	0.45	0.46	0.33	1.00		
**12**	0.28	0.27	0.23	0.32	0.29	0.34	0.31	0.35	1.00	
**13**	0.32	0.25	0.30	0.26	0.28	0.34	0.40	0.29	0.33	1.00

Questions:

Please tell us how much difficulty you have with the following:

1. Taking lots of medications
2. Remembering how and when to take medication
4. Collecting prescription medication
5. Monitoring your medical conditions (eg. checking your blood pressure or blood sugar, monitoring your symptoms etc)
6. Arranging appointments with health professionals
7. Seeing lots of different health professionals
8. Attending appointments with health professionals (eg. getting time off work, arranging transport etc)
11. Obtaining clear and up-to-date information about your condition
12. Making recommended lifestyle changes (eg. diet and exercise)
13. Having to rely on help from family and friends

Please note: Questions 3, 9 and 10 were excluded from the main analysis due to a high proportion of 'does not apply' responses.

For peer review only

OPEN ACCESS

UNIVERSITY OF DUNDEE

University of Dundee

Improving the management of multimorbidity in general practice

Man, Mei See; Chaplin, Katherine; Mann, Cindy; Bower, Peter; Brookes, Sara; Fitzpatrick, Bridie; Guthrie, Bruce; Shaw, Alison; Hollinghurst, Sandra; Mercer, Stewart; Rafi, Imran; Thorn, Joanna; Salisbury, Chris

Published in:
BMJ Open

DOI:
10.1136/bmjopen-2016-011261

Publication date:
2016

Document Version
Final published version

Link to publication in Discovery Research Portal

Citation for published version (APA):
Man, M. S., Chaplin, K., Mann, C., Bower, P., Brookes, S., Fitzpatrick, B., ... Salisbury, C. (2016). Improving the management of multimorbidity in general practice: protocol of a cluster randomised controlled trial (The 3D Study). BMJ Open, 6(4), 1-11. [e011261]. DOI: 10.1136/bmjopen-2016-011261

General rights
Copyright and moral rights for the publications made accessible in Discovery Research Portal are retained by the authors and/or other copyright owners and it is a condition of accessing publications that users recognise and abide by the legal requirements associated with these rights.

- Users may download and print one copy of any publication from Discovery Research Portal for the purpose of private study or research.
- You may not further distribute the material or use it for any profit-making activity or commercial gain.
- You may freely distribute the URL identifying the publication in the public portal.

Take down policy
If you believe that this document breaches copyright please contact us providing details, and we will remove access to the work immediately and investigate your claim.

For peer review only

For peer review only - http://bmjopen.bmj.com/site/about/guidelines.xhtml

Downloaded from http://bmjopen.bmj.com/ on May 3, 2016 - Published by group.bmj.com

BMJ Open

Improving the management of multimorbidity in general practice: protocol of a cluster randomised controlled trial (The 3D Study)

Mei-See Man,[1] Katherine Chaplin,[1] Cindy Mann,[1] Peter Bower,[2] Sara Brookes,[1] Bridie Fitzpatrick,[3] Bruce Guthrie,[4] Alison Shaw,[1] Sandra Hollinghurst,[1] Stewart Mercer,[3] Imran Rafi,[5] Joanna Thorn,[1] Chris Salisbury[1]

To cite: Man M-S, Chaplin K, Mann C, *et al*. Improving the management of multimorbidity in general practice: protocol of a cluster randomised controlled trial (The 3D Study). *BMJ Open* 2016;**6**:e011261. doi:10.1136/bmjopen-2016-011261

▸ Prepublication history and additional material is available. To view please visit the journal (http://dx.doi.org/10.1136/bmjopen-2016-011261).

Received 22 January 2016
Revised 26 February 2016
Accepted 10 March 2016

For numbered affiliations see end of article.

Correspondence to
Professor Chris Salisbury; c.salisbury@bristol.ac.uk

ABSTRACT

Introduction: An increasing number of people are living with multimorbidity. The evidence base for how best to manage these patients is weak. Current clinical guidelines generally focus on single conditions, which may not reflect the needs of patients with multimorbidity. The aim of the 3D study is to develop, implement and evaluate an intervention to improve the management of patients with multimorbidity in general practice.

Methods and analysis: This is a pragmatic two-arm cluster randomised controlled trial. 32 general practices around Bristol, Greater Manchester and Glasgow will be randomised to receive either the '3D intervention' or usual care. 3D is a complex intervention including components affecting practice organisation, the conduct of patient reviews, integration with secondary care and measures to promote change in practice organisation. Changes include improving continuity of care and replacing reviews of each disease with patient-centred reviews with a focus on patients' quality of life, mental health and polypharmacy. We aim to recruit 1383 patients who have 3 or more chronic conditions. This provides 90% power at 5% significance level to detect an effect size of 0.27 SDs in the primary outcome, which is health-related quality of life at 15 months using the EQ-5D-5L. Secondary outcome measures assess patient centredness, illness burden and treatment burden. The primary analysis will be a multilevel regression model adjusted for baseline, stratification/minimisation, clustering and important co-variables. Nested process evaluation will assess implementation, mechanisms of effectiveness and interaction of the intervention with local context. Economic analysis of cost-consequences and cost-effectiveness will be based on quality-adjusted life years.

Ethics and dissemination: This study has approval from South-West (Frenchay) National Health Service (NHS) Research Ethics Committee (14/SW/0011). Findings will be disseminated via final report, peer-reviewed publications and guidance to healthcare professionals, commissioners and policymakers.

Trial registration number: ISRCTN06180958; Pre-results.

Strengths and limitations of this study

- This large trial design draws on considerable evidence about problems experienced by patients with multimorbidity and is based on an evidence-based conceptual framework for how best to improve their management in general practice.
- The healthcare landscape is constantly changing and 'usual care' is variable; therefore, a nested process evaluation will explore how, why and in what contexts the intervention is or is not effective.
- This study is limited by its focus on how the UK National Health Service organises general practice in England and Scotland. The findings may not all be generalisable to countries which have different types of healthcare system.
- Given the lack of a universally agreed definition of multimorbidity, we have defined our multimorbidity study population based on having three or more conditions included in the UK Quality and Outcomes Framework. Although this will include participants with a wide range of disease combinations, different definitions of multimorbidity would lead to inclusion of patients with different characteristics.

INTRODUCTION

An increasing number of people are living with multiple chronic conditions or multimorbidity. At least 16% of adult patients in primary care in the UK have multimorbidity and prevalence increases with age.[1 2] These patients experience a high level of 'illness burden' due to poor quality of life, high rates of depression (which often goes unrecognised) and reduced life expectancy.[2 3] They also experience 'treatment burden' due to having to attend multiple specialist clinics and seeing many different professionals, which can be inconvenient for

For peer review only - http://bmjopen.bmj.com/site/about/guidelines.xhtml

Downloaded from http://bmjopen.bmj.com/ on May 3, 2016 - Published by group.bmj.com

patients as well as inefficient for the health service.[4–6] They may have to take multiple medications in complex regimes.[7] This polypharmacy can be burdensome for patients, increases the likelihood of interactions and adverse effects (including those causing hospital admissions), and may reduce medication adherence.[8–11]

In qualitative studies, patients with multimorbidity describe a lack of holistic patient-centred care, and a concern that no single professional takes overall responsibility for their treatment and treats them as a whole person.[4 5] Current treatment guidelines and professional incentive schemes tend to be focused on individual diseases, which can lead clinicians to focus on disease-based metrics rather than on the problems that are of most concern to the individual with multimorbidity.[12] Many different sets of guidelines can be relevant to one patient with multimorbidity, and attempting to follow all of these guidelines may be excessively burdensome, inefficient and ineffective.[7]

Multimorbidity represents a challenge to healthcare systems as well as to individual patients. Patients with multimorbidity have high rates of primary care consultations and hospital admissions and they account for a disproportionate amount of overall health service expenditure.[13] In the USA, it is estimated that 75% of the healthcare expenditure is spent on treating chronic conditions, while in Europe, the aggregated healthcare cost multiplies with each additional condition (mean cost estimate for three conditions=€1631 compared with €562 for zero conditions).[13] From the healthcare professional's point of view, patients with multimorbidity can be challenging to manage.[14 15] Clinicians express frustration with the lack of time, fragmentation of the healthcare system and inadequate guidelines which limit the care they can offer these patients.[16] Complex medication management is also cited as a particular issue in multimorbidity.[14–16]

The majority of healthcare for people with chronic conditions is provided in primary care, and therefore this should be the main setting for approaches to improve the management of multimorbidity. A recent Cochrane review highlighted the paucity of research on interventions to improve the outcomes of patients with multimorbidity in primary care.[17] Ten studies were identified examining a range of complex interventions which demonstrated mixed effects. The most effective were organisational interventions focused on areas of concern for patients or where they have difficulties, such as functional ability and medication management. No studies included an economic analysis of cost-effectiveness, although a trend towards improved prescribing and medication adherence suggests the potential for cost-savings. The authors of the systematic review called for further pragmatic studies based in primary care settings, using clear definitions of participants and appropriate outcomes.

In summary, patients with multimorbidity experience problems of illness burden (poor quality of life, depression), treatment burden (multiple uncoordinated appointments, polypharmacy) and lack of person-centred care (low continuity, little attention paid to patients' priorities). This research is designed to test the hypothesis that a patient-centred intervention in general practice designed to address the needs and priorities of patients with multimorbidity will improve their health-related quality of life, reduce their burden of illness and treatment and improve their experience of care, while being more cost-effective than conventional service models. This will be examined using a cluster randomised controlled trial (RCT), with economic evaluation and mixed-methods process evaluation.

METHODS AND ANALYSIS

Trial design

This is a multicentre pragmatic, two-arm, practice-level cluster RCT (see figure 1), with parallel mixed-methods process evaluation and economic analysis of cost-effectiveness. The design is based on the Medical Research Council (MRC) framework for the evaluation of complex interventions.[18]

Conceptual framework

The underlying theoretical basis for the intervention is the patient-centred care model.[19–21] This includes four key components, all of which are highly relevant to improving care for patients with multimorbidity:

- A focus on the patient's *individual disease and illness experience*: exploring the main reasons for their visit, their concerns and need for information.
- A *biopsychosocial perspective*: seeking an integrated understanding of the whole person, including their emotional needs and life issues.
- Finding *common ground* on what the problem is and mutually agreeing management plans.
- Enhancing the *continuing relationship* between the patient and doctor (the therapeutic alliance).

The intervention design is based on a conceptual framework which delineates the main problems experienced by patients with multimorbidity (drawing on the existing research evidence) and uses strategies based on the patient-centred care model to seek to address these problems. The general approach has many commonalities with well-recognised frameworks such as the chronic care model[22] and the House of Care.[23]

Participants and setting

This study is based in general practices serving different patient populations in three geographical areas; in and around Bristol, Greater Manchester and Glasgow. Practices in this study will be selected from areas with a range of socioeconomic characteristics, particularly levels of deprivation.

In the UK, each patient is registered with one general practice, typically with between 2 and 10 general practitioners (GPs) and a smaller number of practice nurses.

For peer review only - http://bmjopen.bmj.com/site/about/guidelines.xhtml

Downloaded from http://bmjopen.bmj.com/ on May 3, 2016 - Published by group.bmj.com

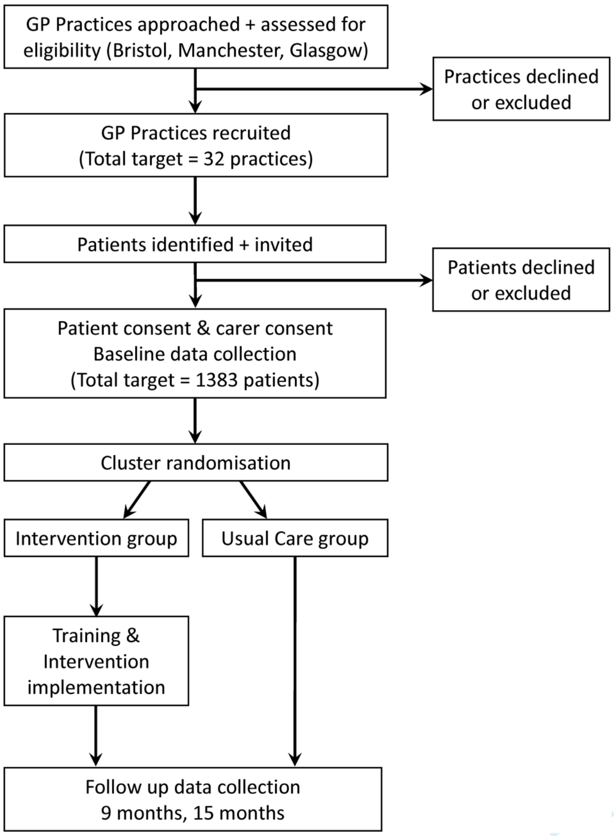

Figure 1 Flow chart of practice and patient recruitment, implementation and follow-up. GP, general practitioner.

Box 1 Chronic conditions for inclusion

Included patients have three or more diagnoses from the following groups of chronic conditions:

- Cardiovascular disease or chronic kidney disease (including coronary heart disease, hypertension, heart failure, peripheral arterial disease, chronic kidney disease stage 3–5)*
- Stroke
- Diabetes
- Chronic obstructive pulmonary disease or asthma*
- Epilepsy
- Atrial fibrillation
- Severe mental health problems (schizophrenia or psychotic illness)*
- Depression
- Dementia
- Learning disability
- Rheumatoid arthritis

*Groups are counted only once even if a patient has multiple conditions within a group. For example, having both hypertension and heart failure would just count for one condition.

Patients receive almost all of their primary medical care from their general practice, which acts as gatekeeper to secondary care services. Patients with multimorbidity are called in for regular review of each of their medical conditions, often having separate reviews for each condition. Many reviews are conducted by nurses who use disease-specific computerised templates to collect relevant data according to clinical guidelines.

Inclusion and exclusion criteria

General practices

To be eligible for inclusion practices need a minimum of three GP partners, a minimum list of 4500 registered patients and to use EMIS Web or EMIS PCS as their computer system. EMIS is the most common clinical records system in UK general practice.

Patients

Inclusion criteria are being aged 18 years or over, being registered with a usual doctor who is participating in the research study and having three of more chronic conditions from those included in the National Health Service (NHS) Quality and Outcomes Framework[24] (QOF, V.31.0)—see box 1.

Exclusion criteria are: having a life expectancy of less than 12 months; serious suicidal risk; known to be leaving the practice within 12 months; unable to complete questionnaires in English even with the help of carers; actively taking part in other research involving extra visits to primary care or other health services; lacking capacity to consent (as coded in their practice records, or determined by their GPs, in Scotland only); being considered unsuitable for the research study by their GP (eg, recently bereaved or currently hospitalised).

Carers

Formal or informal carers of patients consenting to take part in the study will also be invited to contribute by completing a carer's questionnaire. Not all patients may have carers and not all carers may want to take part; therefore, this constitutes a small and separate substudy population.

Recruitment of practices

General practices which are potentially interested in taking part in the trial will be identified with help from the NHS Clinical Research Networks in England and the Scottish Primary Care Network. These nationwide networks facilitate clinical research by identifying and recruiting general practices and providing resources to help practices do research. Local researchers will meet with key stakeholders at the practice (practice manager, GPs, practice nurses) in order to explain the study and its requirement of a commitment to organisational and procedural change. The practice manager or lead GP will sign a practice-level consent agreement.

Recruitment of patients

Each participating practice will be asked to search their practice database using a standard electronic search provided by the research team to identify potentially eligible patients who have three or more chronic conditions as

For peer review only

For peer review only - http://bmjopen.bmj.com/site/about/guidelines.xhtml

Downloaded from http://bmjopen.bmj.com/ on May 3, 2016 - Published by group.bmj.com

defined by the inclusion criteria. In some practices, not all GPs will participate, so in practices which have a policy for patients to see the same GP, only those patients who usually see one of the participating GPs will be included. This is to minimise the potential distress of asking a patient to change their GP for the purpose of the study. If there are more than 150 eligible patients, a simple random sample of 150 of these patients will be selected. GPs will be asked to review the resulting list to screen out patients meeting the exclusion criteria. The practice will send the remaining patients a patient invitation pack including information about the study (see online supplementary appendix 1), a consent form (see online supplementary appendix 2) and baseline questionnaire. Non-respondents will be sent one postal reminder, supplemented by a telephone reminder when possible in practices where recruitment targets are not met.

At sites in England, if a patient lacks capacity to consent, we will obtain the assent of the patient's carer, legal guardian or consultee on behalf of the patient to take part in the study. Carers will be invited to complete a separate carer contact form, and those who wish to participate in the carer's substudy will be sent an information sheet, consent form and baseline questionnaire.

Recruitment of patients began on 20 May 2015 and ended in December 2015. Intervention training began in June 2015 with intervention delivery period starting in August 2015 and due to finish in March 2017.

The intervention

Development

The intervention was developed to address the problems identified in earlier qualitative and quantitative research on the problems experienced by patients with multimorbidity,[25–27] along with experience from previous trials summarised in a systematic review.[17] This was followed by a series of workshops and stakeholder events with patients, carers, health professionals and health service managers. This resulted in a complex intervention with multiple interacting components at the different levels of individual patient–clinician interactions, practice organisation, primary–secondary care integration, and measures to support and incentivise practices to make changes in their services.

Three general practices participated in an external pilot and feasibility study in which the feasibility of the intervention was assessed and improved, and aspects of trial delivery were tested. The views of the patients and healthcare professionals delivering the intervention were fed back to the research team. The key learning points and changes resulting from the optimisation phase are described in online supplementary appendix 3.

Intervention components

The name '3D' was chosen because it acts as a mnemonic for 'dimensions of health; drugs; depression' and also because it alludes to the concept of a holistic, three dimensional perspective. The main components of the final 3D intervention, to be tested in the definitive trial, are illustrated in figure 2 and described below.

The problems experienced by patients with multimorbidity in current care were broadly grouped under the headings of a lack of holistic patient-centred care, high illness burden and high treatment burden. Strategies were identified to try to address each of these problems, as shown in the middle column of figure 2. Finally the specific operational mechanisms or active components of the intervention which will be used to implement each strategy are described in the third column.

Components at practice level relating to organisation of care

The aim is to identify a group of patients with high levels of multimorbidity and on several QOF disease registers in order to prioritise them for a different form of care, recognising that they have more complex needs than most patients. Consenting patients with multimorbidity (as defined in box 1) will be identified and 'flagged' on practice computer systems. They will be allocated a named GP with responsibility for their care (and nurse if possible, particularly in larger practices where several nurses are involved in chronic disease management). These patients will be provided with a '3D' card in order to identify themselves with practice receptionists when booking appointments. The 3D card reminds the patient of their named responsible GP, and encourages them to ask for a longer appointment than usual when they think they need one. This recognises that these patients often need to discuss several problems at one appointment.

In most general practices in the UK, patients with one of the chronic conditions listed in box 1 are invited for review of that condition on a regular basis, such as every 6 or 12 months. At these reviews, the GP or nurse follow computerised disease management templates to collect relevant data about aspects of disease control and management. Patients with multimorbidity may be repeatedly called for separate reviews of each of their chronic conditions, often to see different health professionals, who use different disease management templates which include a large amount of duplication (eg, most templates include measurement of blood pressure and asking about smoking habits). Under the 3D approach, these separate disease-focused reviews will be replaced by a 3D review every 6 months at which all problems will be reviewed at one time.

Components relating to clinicians conduct of reviews

The 3D reviews are comprehensive and, although they include the important aspects of disease management included in single disease reviews, they have a different focus. The conduct of the reviews will be supported by a bespoke 'dynamic' template which automatically personalises for individual patients to only include prompts relevant to the conditions that the patient is recorded as

For peer review only - http://bmjopen.bmj.com/site/about/guidelines.xhtml

Downloaded from http://bmjopen.bmj.com/ on May 3, 2016 - Published by group.bmj.com

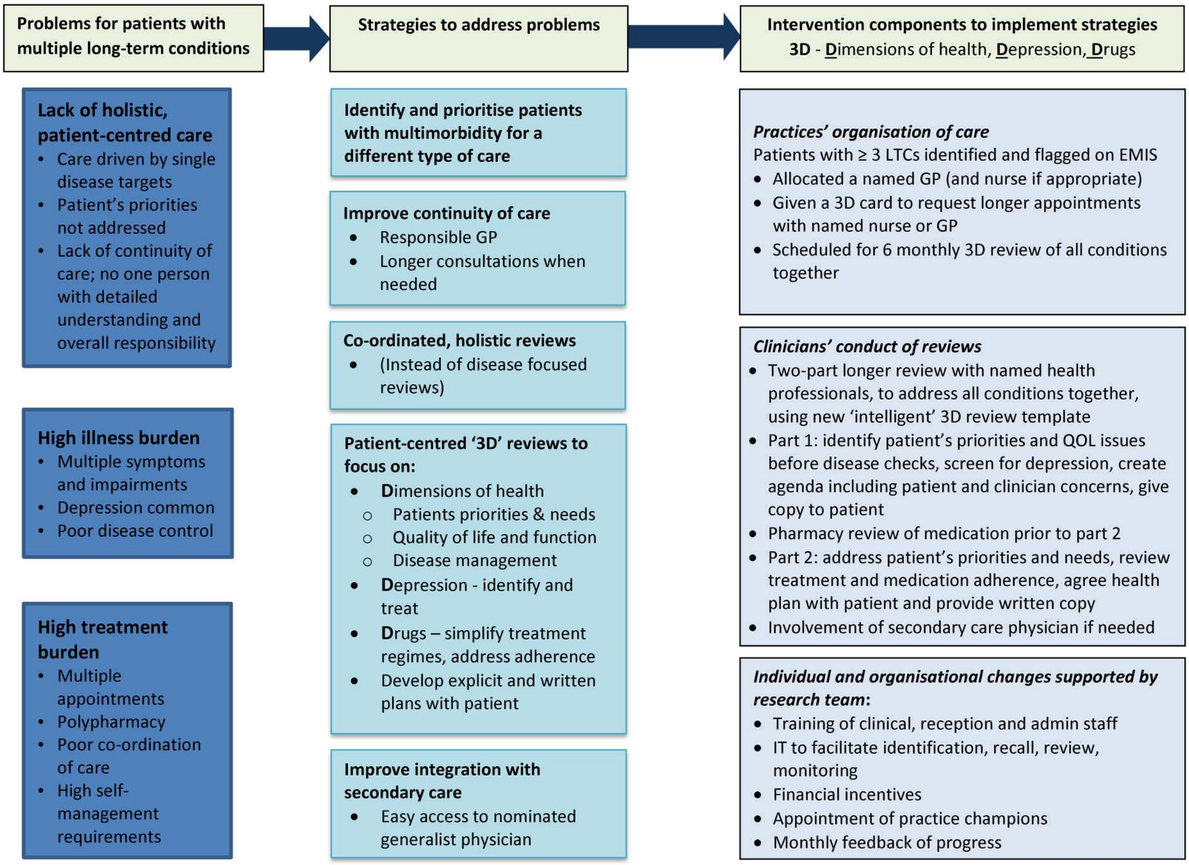

Figure 2 3D logic model. GP, general practitioner; LTC, long term conditions; QOL, quality of life.

having in the electronic medical record. It eliminates the problem of duplication of information between different single disease templates and also provides a structure to encourage the clinicians to enact the 3D approach. The term '3D' acts as a mnemonic to encourage clinicians to focus on the following:

Dimensions of health: This includes first eliciting patients' concerns and priorities for improving their quality of life and function, before collecting data about disease metrics such as weight or blood pressure.

Depression: The clinicians should screen for depression and seek to treat it if identified.

Drugs: In order to address problems of polypharmacy, a pharmacist will review the patient's medical records prior to the 3D review and make recommendations about low priority drugs that might be discontinued, or other ways of simplifying drug regimes, for example, using long-acting medications, so that all tablets can be taken in the morning. The pharmacist review is performed online through remote access to patients' electronic medical records by prior arrangement between the practice and pharmacist. As part of the 3D review, the GP will be trained to ask questions to detect problems with medication adherence and how to help the patient to address this.

Each six-monthly 3D review consists of two appointments. At the first appointment (lasting approximately 30–40 min), the practice nurse will collect information to complete the template and organise all relevant blood tests or other investigations. The nurse review includes collecting information about the patient's priorities for change and aspects of quality of life such as pain and function, and also includes screening for depression using the Patient Health Questionnaire 9 (PHQ9) questionnaire.[28] Following the nurse appointment, the patient will be given a document, known as the '3D agenda', which summarises their assessment and details their top priorities for change. This will set the agenda for the second appointment, approximately 1 week later, with the patient's usual GP. At this 20 min appointment the GP will review all the information collected by the nurse and from the test results, undertake a thorough medication review with the help of the pharmacist's recommendations, seek to address the patient's priorities and problems identified in the assessment, and agree a written care plan for the patient to take away. This 3D Health Plan spells out the specific problems identified (which may be a combination of patients' priorities and problems identified by the nurse or doctor during the review), along with mutually agreed actions that patients and clinicians each have responsibility for. Providing patients with a 3D health plan as a printed summary of their 3D review and test results is intended to promote patient engagement.

For peer review only

For peer review only - http://bmjopen.bmj.com/site/about/guidelines.xhtml

Downloaded from http://bmjopen.bmj.com/ on May 3, 2016 - Published by group.bmj.com

Each general practice will be allocated a designated 'generalist physician' (usually a geriatrician) in secondary care whom they can contact to discuss individuals with complex problems and (if possible) to help coordinate use of hospital investigations and appointments where patients are attending numerous different specialist clinics or having multiple hospital-based tests on different days.

Components relating to supporting practices

In order to support the implementation of the intervention, the study team developed a training package of two half-day sessions for healthcare professionals. These sessions are facilitated by a clinician trainer and at least one local researcher, covering tasks and discussion topics including eliciting of patient concerns, exploring strategies to promote patient-centred care, ways to improve continuity of care, negotiating a patient health plan, improving medication adherence, the aims of the 3D reviews and use of the 3D review template. A substantial element of the training will be devoted to promoting attitudinal change among clinicians towards identifying and responding to patients' own priorities and problems with broader quality of life, as organisational change is unlikely to be effective unless clinicians 'buy into' the underlying philosophy of the new approach.[29] Practice receptionists will also be offered training in promoting continuity of care and offering longer appointments to patients with multimorbidity.

A number of other strategies are being followed to promote implementation of the intervention within practices. In addition to the bespoke computerised 3D review template, we have also developed software to facilitate identification and monitoring of the participants. Financial reimbursement is provided to practices to cover the costs of practice staff training and setting up of the necessary patient recall systems. Modest financial incentives (£60 per patient) are also provided to practices based on the number of patients that complete both of their six-monthly 3D reviews within the 15-month follow-up period. Each practice will be asked to nominate a GP champion to help monitor and promote the intervention within the practice, and also to meet and share good ideas and experiences with other GP champions in local collaboratives. The practice champions will be provided with monthly feedback reports about their practice's progress in implementing the reviews. We will allow local adaptation of the intervention to reflect local context while ensuring the key elements of the conceptual framework (those shown in figure 2) are maintained.[30]

Control group

Patients in practices allocated to the control arm will continue to receive care as usual. In most practices, this will mean patients are recalled to different clinics to see different practice nurses to review each of their long-term conditions. The nurses will usually follow disease-specific computerised protocols for their management, and will mainly focus on collecting data related to QOF targets rather than quality of life or patients' priorities. The nature of 'care as usual' may vary between practices and over time—this will be explored in the process evaluation.

Participant withdrawal

Among intervention practices, if any participant later requests not to receive the 3D intervention, they will revert to the usual care provided for other patients in their practice. Unless a patient requests to withdraw from the trial they will continue to be followed up and will be analysed in the group to which the practice was allocated. If they wish to withdraw from the trial, then no further follow-up data will be requested but data already provided will be used.

Outcome measures

Primary outcome measures

The primary outcome for patient and carers will be health-related quality of life (HRQoL) as measured by the EQ-5D-5L after 15 months following patient recruitment.[31] The EQ-5D is a widely used self-reported generic measure of HRQoL which has been validated in many different patient populations including diabetes, cardiovascular problems, chronic obstructive pulmonary disease, cancer, chronic pain and rheumatoid arthritis. The five-level version (EQ-5D-5L) contains the same dimensions as the earlier three-level version (EQ-5D-3L) but has been designed to provide greater reliability and sensitivity.

Secondary outcomes

Secondary outcome measures for participants are grouped under domains as shown in box 2.

Secondary outcome measures for carers will assess measures of carer quality of life and strain, including the EQ-5D-5L,[31] the Carer Experience Scale[39] and the Brief Treatment Burden Questionnaire for carers. These will be reported separately, as they are not participant outcomes.

Measures of process of care

We will monitor processes in the intervention practices in order to report the degree of implementation of the intervention. This will include the number of nurse and GP 3D reviews undertaken, the extent to which the 3D template was fully completed, the number of pharmacy reviews performed, whether an agenda and health plan were created and printed off to give to the patient, and the number of times the hospital general physician was contacted.

The continuity of care (COC) measure[40] will be used as a measure of longitudinal continuity, for all telephone or face-to-face consultations by participants with GPs or nurses within the practice over the 15-month follow-up

For peer review only - http://bmjopen.bmj.com/site/about/guidelines.xhtml

Downloaded from http://bmjopen.bmj.com/ on May 3, 2016 - Published by group.bmj.com

Box 2 Secondary outcomes for patients participating in the 3D trial

Experience of holistic patient-centred care

- Consultation and Relational Empathy (CARE) measure of relational continuity in general practitioner and nurse* consultations[32]
- Coordination of care (two questions from LTC6 Quality Innovation Productivity and Prevention (QIPP) programme)
- Patient Assessment of Chronic Illness Care (PACIC) measure[33]
- Overall satisfaction (single item)

Burden of illness measures

- Self-rated health
- Illness burden in multimorbidity (Bayliss)[34]
- Quality of disease management (a composite measure of Quality and Outcomes Framework (QOF) achievement)[35]
- Hospital Anxiety Depression Scale (HADS)[36]

Burden of treatment

- Brief Treatment Burden Questionnaire†
- Morisky Medication Adherence Scale (eight-item)[37]
- Number of prescribed drugs
- Number of high-risk drug combinations[38]

*Not collected at 9 months follow-up

†New measure developed for this study, based on qualitative interviews, item generation, principle components analysis and testing of psychometric properties.

period, adjusted for continuity in the 15 months before the intervention.

Although also required for the economic analysis, the number of primary care consultations and the number of hospital admissions will be of particular interest as indicators of the effect of the intervention on primary and secondary health services.

We will report descriptively the systems in place to provide care for patients with multimorbidity in practices in both arms of the trial at baseline and at the end of the 15-month follow-up period, in particular to capture whether there are differences in 'usual care' in the control arm practices over the period of this study.

Economic evaluation

The economic evaluation will be undertaken from the perspectives of (1) NHS and personal social services (PSS) and (2) patients. We will compare the extra cost of caring for patients in the intervention group with the difference in outcome as measured by the EQ-5D-5L and related quality-adjusted life years (QALYs). Resource use data will be collected from patient self-reported postal questionnaires at baseline, 9 and 15 months and GP practice records. The questionnaires will ask about the use of community and secondary care health services, social services, informal care, and personal costs (including travel, loss of earnings and dependent care costs). Patients indicating use of hospital services will be contacted by telephone to obtain more detail about the inpatient stay or accident and emergency visit. GP practice records will be used to obtain information about all available primary care contacts, including type of consultation and who was seen, tests and investigations, and prescribed medication.

Trial records will be used to estimate the cost of setting up the 3D service and training staff. This will be identified and reported separately from the running costs.

NHS resources will be valued using national published sources such as Curtis,[41] NHS reference costs[42] and the British National Formulary (BNF).[43]

Data collection

At baseline, data will be collected on the sociodemographic measures (number of long-term conditions; age; gender; education; ethnicity; deprivation status (index of multiple deprivation based on postcode); work status) and all primary and secondary outcomes. The primary outcome will be collected 9 and 15 months after recruitment, with the primary outcome time point being at 15 months. All but one of the secondary outcomes will be collected at 9 months, as shown in box 2. All secondary outcomes, measures of the process of care and measures of resource utilisation will be collected 15 months after recruitment. Practice randomisation occurs after patient recruitment, and it then takes approximately 3 months to train practices to deliver the 3D intervention. Patients have their 3D reviews on a six-monthly cycle. Therefore, collecting outcome data 9 and 15 months after patient recruitment allows for a 3-month lag time and ensures that most patients will be invited to have two 3D reviews before outcomes are measured.

The primary method of self-reported data collection will be via postal questionnaires; however, alternative completion methods including by telephone or via a home visit by a researcher masked to treatment allocation will be offered if necessary in order to maximise response rates.

Two reminders, the first by letter or email (approximately 10–14 days after posting the questionnaire) and the second by phone (approximately 10–14 days after the first reminder), will be made for participants who have not returned their questionnaire. Patients will be given £5 gift vouchers for completion of questionnaires.

No data about identifiable patients will leave the practice unless patients have provided consent. All data will be stored securely and confidentially at the University of Bristol in line with its data management policies.

Sample size

The study is designed to detect an effect size of 0.274 SDs in the primary outcome of the EQ-5D-5L. Data about the variability of the new five-level (5L) version of the EQ-5D is currently more limited than for the well-established three-level (3L) version. The SD of the EQ-5D-3L in the UK general population is 0.23, rising to 0.27 in the oldest respondents (aged over 75).[44] Hence, an effect size of 0.274 would equate to a detectable

For peer review only - http://bmjopen.bmj.com/site/about/guidelines.xhtml

Downloaded from http://bmjopen.bmj.com/ on May 3, 2016 - Published by group.bmj.com

difference of (0.274×0.27)=0.074 on the EQ-5D-3L, previously deemed to be the minimum important difference.[45] Although there are less data about the variability in the 5L version of the EQ-5D than the 3L version, this latest version is likely to have greater sensitivity to change.[31]

Based on data available from our previous studies,[1] we estimated that 2.3% of adult patients would have multimorbidity as defined in this study. This equates to about 108 patients in an average-sized practice of 6000 patients. Recruiting 32 practices would therefore provide 3456 potentially eligible patients. Assuming 40% of patients agree to participate (n=1382), 80% are followed up to 12 months, and an intraclass correlation coefficient (ICC) of 0.03 for clustering at the practice level (based on the WISE trial),[46] 32 practices will provide approximately 90% power, with a 5% α level to detect an effect size of 0.274 SDs in the EQ-5D-5L measure between the intervention and control groups.

Allocation

General practices will be the unit of allocation. Practices will be allocated in a 1:1 ratio to receive either the intervention or continue care as usual (control group). Randomisation will be stratified by area (Bristol, Greater Manchester, Glasgow) and minimised by deprivation level and practice size. Within each area allocation will be performed in blocks of two, with both practices in a block randomised at the same time and released to the trial manager together to ensure allocation concealment and no selection bias. It was not deemed possible to increase or vary the block sizes given the small number of practices recruited to each area and the dynamic nature of recruitment. The trial manager will notify the local research team of the two allocations and they will then notify the practices and arrange training of the intervention practice. The allocation schedule will be computer-generated by the trial statistician, blind to details of the practices apart from those needed for stratification and minimisation.

Randomisation of a practice will take place after patients in that practice have been identified and invited to participate in order to avoid selection bias.

Blinding

Once participants have been recruited, it will not be possible to mask participants or healthcare professionals to the group allocation of their practice. It is also not feasible to blind all members of the study team actively involved in the execution of the study. However, data entry and checks of data quality will be conducted by administrative staff masked to treatment allocation. Analysis of outcomes will be performed by the trial statistician, also masked to treatment allocation.

Statistical methods

Data will be analysed in accordance with CONSORT principles and its extension for cluster randomised trials. Descriptive statistics will be used to summarise characteristics of practices and patients and compare baseline characteristics between groups. A full statistical analysis plan will be developed and agreed by the Data Monitoring Committee (DMC) and the Trial Steering Committee (TSC) after completion of the pilot phase and prior to undertaking any analyses of the main trial.

All analyses of primary and secondary outcomes will be at the patient level and will account for clustering by practice using multilevel regression models. Analyses will be performed on an 'as allocated' basis. Primary analysis comparing EQ-5D-5L between the intervention and control practices will employ a linear multilevel regression model adjusted for stratification/minimisation variables. Subsequent models will adjust for baseline EQ-5D-5L, any variables demonstrating imbalance at baseline and other important prognostic variables such as age, number of long-term conditions, deprivation and depression. Preplanned analyses of secondary outcomes will also employ linear or logistic (as appropriate) multilevel regression models.

Formal tests of interaction will be performed to consider the following potential effect modifiers: age, number of chronic conditions, index of deprivation, and presence or absence of depression alongside physical health problems. The trial is not specifically powered for such interaction tests; hence, interpretation will focus on the CIs and will be hypothesis-generating only. The potential impact of missing data will be examined through sensitivity analyses.

Anonymised data will be used in order to compare descriptive data for consenting versus non-consenting patients. We will explore the possibility of comparing QOF performance in patients with chronic conditions both with and without multimorbidity—this is to assess for the potential unintended consequence that concentrating effort on patients with multimorbidity may have a positive or negative impact on the care of other patients.

No interim analyses are planned.

Economic analysis

Cost per patient will be estimated by applying unit costs to the resources used. In a cost-consequences analysis, we will relate the mean cost per participant in each group with changes in a range of outcomes; cost-effectiveness analysis from the NHS and PSS perspective will estimate the incremental cost per QALY gain where QALYs are estimated using the EQ-5D-5L. Uncertainty will be addressed in sensitivity analyses and by using bootstrapping to estimate the net monetary benefit and a cost-effectiveness acceptability curve.

Process evaluation

Alongside the main analysis of quantitative outcomes from the trial, we are conducting a nested process evaluation. This mixed-methods study aims to better understand how and why the intervention was effective or ineffective and to identify contextually relevant strategies

For peer review only - http://bmjopen.bmj.com/site/about/guidelines.xhtml

Downloaded from http://bmjopen.bmj.com/ on May 3, 2016 - Published by group.bmj.com

for successful implementation as well as practice difficulties in adoption, delivery and maintenance of the intervention. Further details of the protocol for the process evaluation will be published in a separate paper.[47]

ETHICS

Ethics approval

This study will be conducted in accordance with principles of good clinical practice.

Patients will not be denied any form of care that is currently available in the NHS by participating in this study. Patients from usual care practices will still have access to all locally recommended treatments and services. Patients from intervention practices will still have full access to their GP and secondary care services in addition to their six-monthly 3D assessments. Any changes in medication prescribing will be performed by a GP in the context of normal clinical care.

Patient safety

We will monitor and report descriptively the numbers of serious adverse events in each arm which appeared to be related to the intervention or the trial, and also the number of deaths in each trial arm. Given that patients with multimorbidity may be heavy users of secondary care services, new medical diagnoses, hospital admissions and deaths are expected and will not be considered as potential serious adverse events unless anyone involved in the study (participants, general practice staff or research staff) notify the research team of any events that they consider may have been related to the intervention or the research process. All deaths will be investigated for relatedness by requesting the patient's GP provide details of cause of death and relatedness to study.

Study management and oversight

The 3D Study is managed by the Trial Management Group, consisting of the chief investigator, principal investigators and researchers from each of the recruiting sites and other co-applicants. There is additional governance oversight by an independent TSC and an independent DMC, both constituted in line with guidance from the National Institute for Health Research (NIHR). An advisory group with members from key local and national stakeholder organisations and lay members has been convened to provide advice about the wider context, other related initiatives and to facilitate communication and eventual knowledge mobilisation with regard to this trial. There is an active patient and carer forum which meets regularly to advise on the design and conduct of the study.

The project will seek to maximise the impact of the research by adopting a model of knowledge transfer. We aim to disseminate our findings to patients, healthcare professionals, commissioners and other academics. In addition to publication of study results, guides for commissioners and for practices will be produced to enable wider implementation of the new 3D approach. The RCGP Clinical Innovation and Research Centre will facilitate wide dissemination to practices and the production of these resources.

The research team is committed to full publication of the results. Authorship will be in accordance with the guidance of the International Committee of Medical Journal Editors. All authors will have full access to the study data. Once the main results have been published, data may be available to other investigators subject to agreement about the protocol with the chief investigator and compliance with policies of the funder and sponsor in relation to data sharing. The study sponsor and the funder will have no role in study design, data collection, management, analysis or interpretation of data, writing of the final report or the decision to submit for publication.

DISCUSSION

This large and rigorous trial will provide robust evidence about the benefits and costs of a pragmatic intervention to improve the management of multimorbidity in general practice. It builds on a considerable evidence base about the difficulties experienced by patients with multimorbidity and the health professionals who seek to care for them. Through the use of a patient-centred conceptual framework, it tests a range of strategies which should address these difficulties and improve outcomes that matter to patients. The study is highly pragmatic.[48] It is based in a range of normal general practice settings and in the different health economies of England and Scotland, which will enhance generalisability. It includes patients with broad inclusion criteria and few exclusion criteria, and assesses a wide range of outcomes including those relating to health status, patient experience and resource utilisation. Implementation of the intervention is flexible to local context, but the extent to which the intervention adheres to the key-intended principles will be monitored.

The study is being conducted with considerable attention to principles of knowledge translation. If the intervention is effective, it will be possible to roll it out quickly to general practices across the UK, and the 3D approach is also likely to be applicable to the management of patients with multimorbidity in many other countries.

Author affiliations
[1]Centre for Academic Primary Care, School of Social and Community Medicine, University of Bristol, Bristol, UK
[2]Centre for Primary Care, Institute of Population Health, University of Manchester, Manchester, UK
[3]Institute of Health and Wellbeing, College of Medical, Veterinary and Life Sciences, University of Glasgow, Glasgow, UK
[4]Quality, Safety and Informatics Research Group, University of Dundee, Dundee, UK
[5]Clinical Innovation and Research, Royal College of General Practitioners, London, UK

For peer review only - http://bmjopen.bmj.com/site/about/guidelines.xhtml

Downloaded from http://bmjopen.bmj.com/ on May 3, 2016 - Published by group.bmj.com

Twitter Follow authors from the Centre for Academic Primary Care at @CAPCBristol. www.bristol.ac.uk/3d-study

Acknowledgements The authors would like to thank Bristol Clinical Commissioning Group (CCG) for hosting this research, in particular Emma Moody, Joanne Atkinson and Rebecca Robinson. The authors thank the Avon Primary Care Research Collaborative for their help and support, PRIMIS for developing the search and templates, and John McLeod and Keith Moffat for developing the EMIS PCS version of the template. They would also like to thank members of the independent TSC, DMC, advisory group, and public and patient involvement group for their advice and input into the design and conduct of the study. Finally, they would like to thank the pilot practices, patients and trainers for their feedback in developing and testing the intervention.

Contributors CS conceived the original study. CS, PB, SM, BG, IR, SB, AS, SH and CM are co-applicants on the funding application. M-SM led the writing of the first draft of the paper with contribution from SB (statistical analyses), SH and JT (questionnaire outcomes and economic analyses), KC and CM (intervention development and optimisation). All authors contributed to the development of the protocol and to the editing of this manuscript.

Funding This project was funded by the National Institute for Health Research Health Services and Delivery Research Programme (project number 12/130/15). The trial sponsor is the University of Bristol, (Senate House, Tyndall Avenue, Bristol BS8 1TH, UK).

Disclaimer The views and opinions expressed therein are those of the authors and do not necessarily reflect those of the HS&DR Programme, NIHR, NHS or the Department of Health.

Competing interests None declared.

Ethics approval South-West (Frenchay) NHS Research Ethics Committee (14/SW/0011) and local NHS R&D approvals from the appropriate participating trusts.

Provenance and peer review Not commissioned; externally peer reviewed.

Data sharing statement Once the main results have been published, data may be available to other investigators subject to agreement about the protocol with the chief investigator and compliance with policies of the funder and sponsor in relation to data sharing.

Open Access This is an Open Access article distributed in accordance with the terms of the Creative Commons Attribution (CC BY 4.0) license, which permits others to distribute, remix, adapt and build upon this work, for commercial use, provided the original work is properly cited. See: http://creativecommons.org/licenses/by/4.0/

REFERENCES

1. Salisbury C, Johnson L, Purdy S, *et al*. Epidemiology and impact of multimorbidity in primary care: a retrospective cohort study. *Br J Gen Pract* 2011;61:e12–21.
2. Barnett K, Mercer S, Norbury M, *et al*. The epidemiology of multimorbidity in a large cross-sectional dataset: implications for health care, research and medical education. *Lancet* 2012;380:37–43.
3. Lawson KD, Mercer SW, Wyke S, *et al*. Double trouble: the impact of multimorbidity and deprivation on preference-weighted health related quality of life a cross sectional analysis of the Scottish Health Survey. *Int J Equity Health* 2013;12:67.
4. Bayliss EA, Edwards AE, Steiner JF, *et al*. Processes of care desired by elderly patients with multimorbidities. *Fam Pract* 2008;25:287–93.
5. Noël PH, Chris Frueh B, Larme AC, *et al*. Collaborative care needs and preferences of primary care patients with multimorbidity. *Health Expect* 2005;8:54–63.
6. Bayliss EA, Ellis JL, Steiner JF. Barriers to self-management and quality-of-life outcomes in seniors with multimorbidities. *Ann Fam Med* 2007;5:395–402.
7. Hughes LD, McMurdo ME, Guthrie B. Guidelines for people not for diseases: the challenges of applying UK clinical guidelines to people with multimorbidity. *Age Ageing* 2013;42:62–9.
8. Marcum ZA, Gellad WF. Medication adherence to multidrug regimens. *Clin Geriatr Med* 2012;28:287–300.
9. Bourgeois FT, Shannon MW, Valim C, *et al*. Adverse drug events in the outpatient setting: an 11-year national analysis. *Pharmacoepidemiol Drug Saf* 2010;19:901–10.
10. Guthrie B, Payne K, Alderson P, *et al*. Adapting clinical guidelines to take account of multimorbidity. *BMJ* 2012;345:e6341.
11. Dumbreck S, Flynn A, Nairn M, *et al*. Drug-disease and drug-drug interactions: systematic examination of recommendations in 12 UK national clinical guidelines. *BMJ* 2015;350:h949.
12. Chew-Graham CA, Hunter C, Langer S, *et al*. How QOF is shaping primary care review consultations: a longitudinal qualitative study. *BMC Fam Pract* 2013;14:103.
13. Glynn LG, Valderas JM, Healy P, *et al*. The prevalence of multimorbidity in primary care and its effect on health care utilization and cost. *Fam Pract* 2011;28:516–23.
14. Smith SM, O'Kelly S, O'Dowd T. GPs' and pharmacists' experiences of managing multimorbidity: a Pandora's box. *Br J Gen Pract* 2010;60:e285–94.
15. O'Brien R, Wyke S, Guthrie B, *et al*. An 'endless struggle': a qualitative study of general practitioners' and practice nurses' experiences of managing multimorbidity in socio-economically deprived areas of Scotland. *Chronic Illn* 2011;7:45–59.
16. Sinnott C, Mc Hugh S, Browne J, *et al*. GPs' perspectives on the management of patients with multimorbidity: systematic review and synthesis of qualitative research. *BMJ Open* 2013;3:e003610.
17. Smith SM, Soubhi H, Fortin M, *et al*. Managing patients with multimorbidity: systematic review of interventions in primary care and community settings. *BMJ* 2012;345:e5205.
18. Craig P, Dieppe P, Macintyre S, *et al*. Developing and evaluating complex interventions: the new Medical Research Council guidance. *BMJ* 2008;337:a1655.
19. Stewart M. Towards a global definition of patient centred care. *BMJ* 2001;322:444–5.
20. American Geriatric Society Expert Panel. Patient-centered care for older adults with multiple chronic conditions: a stepwise approach from the American Geriatrics Society: American Geriatrics Society expert panel on the care of older adults with multimorbidity. *J Am Geriatr Soc* 2012;60:1957–68.
21. Mead N, Bower P. Patient-centredness: a conceptual framework and review of the empirical literature. *Soc Sci Med (1982)* 2000;51:1087–110.
22. Wagner EH, Austin BT, Von KM. Improving outcomes in chronic illness. *Manag Care Q* 1996;4:12–25.
23. Coulter A, Roberts S, Dixon A. Delivering better services for people with long-term conditions: building the house of care. Secondary delivering better services for people with long-term conditions: building the house of care 2013. http://www.kingsfund.org.uk/sites/files/kf/field/field_publication_file/delivering-better-services-for-people-with-long-term-conditions.pdf
24. Health and Social Care Information Centre. Quality and Outcomes Framework. 2015. http://www.hscic.gov.uk/qof (accessed 10 Oct 2015).
25. Salisbury C. Multimorbidity: redesigning health care for people who use it. *Lancet* 2012;380:7–9.
26. Salisbury C. Multimorbidity: time for action rather than words. *Br J Gen Pract* 2013;63:64–5.
27. Wallace E, Salisbury C, Guthrie B, *et al*. Managing patients with multimorbidity in primary care. *BMJ* 2015;350.
28. Kroenke K, Spitzer R, Williams JB. The PHQ-9 validity of a Brief Depression Severity Measure. *J Gen Intern Med* 2001;16:606–13.
29. Year of Care: report of findings from the pilot programme. 2011. http://www.networks.nhs.uk/nhs-networks/national-pbc-clinical-leaders-network/documents/YOC_Report.pdf
30. Hawe P, Shiell A, Riley T. Complex interventions: how "out of control" can a randomised controlled trial be? *BMJ* 2004;328:1561–3.
31. EuroQol Group. EuroQol Group EQ-5D-5L. Secondary EuroQol Group EQ-5D-5L. http://www.euroqol.org/about-eq-5d/valuation-of-eq-5d/eq-5d-5l-value-sets.html
32. Mercer SW, McConnachie A, Maxwell M, *et al*. Relevance and practical use of the Consultation and Relational Empathy (CARE) measure in general practice. *Fam Pract* 2005;22:328–34.
33. Glasgow RE, Wagner EH, Schaefer J, *et al*. Development and validation of the Patient Assessment of Chronic Illness Care (PACIC). *Med Care* 2005;43:436–44.
34. Bayliss EA, Ellis JL, Steiner JF. Seniors' self-reported multimorbidity captured biopsychosocial factors not incorporated into two other data-based morbidity measures. *J Clin Epidemiol* 2009;62:550–7.e1.
35. Reeves D, Campbell SM, Adams J, *et al*. Combining multiple indicators of clinical quality—an evaluation of different analytic approaches. *Med Care* 2007;45:489–96.

For peer review only - http://bmjopen.bmj.com/site/about/guidelines.xhtml

Downloaded from http://bmjopen.bmj.com/ on May 3, 2016 - Published by group.bmj.com

36. Zigmond AS, Snaith RP. The hospital anxiety and depression scale. *Acta Psychiatr Scand* 1983;67:361–70.
37. Morisky DE, Green LW, Levine DM. Concurrent and predictive validity of a self-reported measure of medication adherence. *Med Care* 1986;24:67–74.
38. Guthrie B, McCowan C, Davey P, *et al*. High risk prescribing in primary care patients particularly vulnerable to adverse drug events: cross sectional population database analysis in Scottish general practice. *BMJ* 2011;342:d3514.
39. Al-Janabi H, Flynn TN, Coast J. Estimation of a preference-based carer experience scale. *Med Decis Making* 2011;31:458–68.
40. Bice TW, Boxerman SB. A quantitative measure of continuity of care. *Med Care* 1977;15:347–9.
41. Curtis L. *Unit costs of health and social care*. Canterbury, Kent: Personal Social Services Research Unit, 2012:1–272.
42. Department of Health. NHS Reference Costs. 2014. http://www.gov.uk/government/collections/nhs-reference-costs (accessed 10 Oct 2015).
43. British Medical Association, Royal Pharmaceutical Society. *British National Formulary*. London: BMJ Group and Pharmaceutical Press, 2015.
44. Kind P, Hardman G, Macran S. *UK population norms for EQ-5D*. York: University of York, 1999:98.
45. Walters SJ, Brazier JE. Comparison of the minimally important difference for two health state utility measures: EQ-5D and SF-6D. *Qual Life Res* 2005;14:1523–32.
46. Kennedy A, Bower P, Reeves D, *et al*. Implementation of self management support for long term conditions in routine primary care settings: cluster randomised controlled trial. *BMJ* 2013;346:f2882.
47. Mann C, A Shaw A, Guthrie B, *et al*. Protocol for the process evaluation of a cluster randomised controlled trial to improve management of multi-morbidity in general practice (The 3D study). *BMJ Open* 2016. In press. doi:10.1136/bmjopen-2016-011260
48. Loudon K, Treweek S, Sullivan F, *et al*. The PRECIS-2 tool: designing trials that are fit for purpose. *BMJ* 2015;350:h2147.

For peer review only

For peer review only - http://bmjopen.bmj.com/site/about/guidelines.xhtml

Downloaded from http://bmjopen.bmj.com/ on May 3, 2016 - Published by group.bmj.com

Improving the management of multimorbidity in general practice: protocol of a cluster randomised controlled trial (The 3D Study)

Mei-See Man, Katherine Chaplin, Cindy Mann, Peter Bower, Sara Brookes, Bridie Fitzpatrick, Bruce Guthrie, Alison Shaw, Sandra Hollinghurst, Stewart Mercer, Imran Rafi, Joanna Thorn and Chris Salisbury

BMJ Open 2016 6:
doi: 10.1136/bmjopen-2016-011261

Updated information and services can be found at:
http://bmjopen.bmj.com/content/6/4/e011261

These include:

References
This article cites 38 articles, 20 of which you can access for free at:
http://bmjopen.bmj.com/content/6/4/e011261#BIBL

Open Access
This is an Open Access article distributed in accordance with the terms of the Creative Commons Attribution (CC BY 4.0) license, which permits others to distribute, remix, adapt and build upon this work, for commercial use, provided the original work is properly cited. See: http://creativecommons.org/licenses/by/4.0/

Email alerting service
Receive free email alerts when new articles cite this article. Sign up in the box at the top right corner of the online article.

Topic Collections
Articles on similar topics can be found in the following collections

General practice / Family practice (444)
Geriatric medicine (183)
Health services research (928)
Patient-centred medicine (285)

Notes

To request permissions go to:
http://group.bmj.com/group/rights-licensing/permissions

To order reprints go to:
http://journals.bmj.com/cgi/reprintform

To subscribe to BMJ go to:
http://group.bmj.com/subscribe/

For peer review only

Qual Life Res
DOI 10.1007/s11136-012-0344-y

ISOQOL recommends minimum standards for patient-reported outcome measures used in patient-centered outcomes and comparative effectiveness research

Bryce B. Reeve · Kathleen W. Wyrwich · Albert W. Wu · Galina Velikova · Caroline B. Terwee · Claire F. Snyder · Carolyn Schwartz · Dennis A. Revicki · Carol M. Moinpour · Lori D. McLeod · Jessica C. Lyons · William R. Lenderking · Pamela S. Hinds · Ron D. Hays · Joanne Greenhalgh · Richard Gershon · David Feeny · Peter M. Fayers · David Cella · Michael Brundage · Sara Ahmed · Neil K. Aaronson · Zeeshan Butt

Accepted: 17 December 2012
Springer Science+Business Media Dordrecht 2013

Abstract

Purpose An essential aspect of patient-centered outcomes research (PCOR) and comparative effectiveness research (CER) is the integration of patient perspectives and experiences with clinical data to evaluate interventions. Thus, PCOR and CER require capturing patient-reported outcome (PRO) data appropriately to inform research, health-care delivery, and policy. This initiative's goal was to identify minimum standards for the design and selection of a PRO measure for use in PCOR and CER.

Methods We performed a literature review to find existing guidelines for the selection of PRO measures. We also conducted an online survey of the International Society for Quality of Life Research (ISOQOL) membership to solicit input on PRO standards. A standard was designated as "recommended" when >50 % respondents endorsed it as "required as a minimum standard."

Results The literature review identified 387 articles. Survey response rate was 120 of 506 ISOQOL members. The respondents had an average of 15 years experience in PRO research, and 89 % felt competent or very competent providing feedback. Final recommendations for PRO measure standards included: documentation of the conceptual and measurement model; evidence for reliability, validity (content validity, construct validity, responsiveness); interpretability of scores; quality translation, and acceptable patient and investigator burden.

Conclusion The development of these minimum measurement standards is intended to promote the appropriate use of PRO measures to inform PCOR and CER, which in turn can improve the effectiveness and efficiency of healthcare delivery. A next step is to expand these

This study was conducted on behalf of the International Society for Quality of Life Research (ISOQOL).

Electronic supplementary material The online version of this article (doi:10.1007/s11136-012-0344-y) contains supplementary material, which is available to authorized users.

B. B. Reeve (✉)
Department of Health Policy and Management, Gillings School of Global Public Health, University of North Carolina at Chapel Hill, 1101-D McGavran-Greenberg Building, 135 Dauer Drive, CB 7411, Chapel Hill, NC 27599-7411, USA
e-mail: bbreeve@email.UNC.edu

B. B. Reeve · J. C. Lyons
Lineberger Comprehensive Cancer Center, University of North Carolina at Chapel Hill, Chapel Hill, NC, USA

K. W. Wyrwich · D. A. Revicki · W. R. Lenderking
United BioSource Corporation, Bethesda, MD, USA

A. W. Wu · C. F. Snyder
Johns Hopkins School of Medicine, Baltimore, MD, USA

G. Velikova · J. Greenhalgh
University of Leeds, Leeds, UK

C. B. Terwee
VU University Medical Center, Amsterdam, The Netherlands

C. Schwartz
DeltaQuest Foundation, Inc., Concord, MA, USA

C. M. Moinpour
Fred Hutchinson Cancer Research Center, Seattle, WA, USA

L. D. McLeod
Research Triangle Institute Health Solutions, Durham, NC, USA

Published online: 04 January 2013

For peer review only - http://bmjopen.bmj.com/site/about/guidelines.xhtml

minimum standards to identify best practices for selecting decision-relevant PRO measures.

Keywords Patient-reported outcomes · Comparative effectiveness · Patient-centered outcomes research · Psychometrics · Questionnaire

Introduction

An essential aspect of patient-centered outcomes research (PCOR) and comparative effectiveness research (CER) is the integration of patients' perspectives about their health with clinical and biological data to evaluate the safety and effectiveness of interventions. Such integration recognizes that health-related quality of life (HRQOL) and how it is affected by disease and treatment complements traditional clinical endpoints such as survival or tumor response in cancer. For HRQOL endpoints, it is widely accepted that the patient's report is the best source of information about what he or she is experiencing. The challenge for PCOR and CER is how to best capture patient-reported data in a way that can inform decision making in healthcare delivery, research, and policy settings.

Observational and experimental studies have increasingly included patient-reported outcome (PRO) measures, defined by the Food and Drug Administration (FDA) as "any report of the status of a patient's health condition that comes directly from the patient, without interpretation of the patient's response by a clinician or anyone else [1]." Patients can report accurately on a number of domains that are important for evaluating an intervention or disease burden, including symptom experiences (e.g., pain, fatigue, nausea), functional status (e.g., sexual, bowel, or urinary functioning), well-being (e.g., physical, mental, social), quality of life, and satisfaction with care or with a treatment [1–4]. Arguably, patients are the gold standard source of information for assessing such domains. To draw valid research conclusions regarding patient-centered outcomes, PROs must be measured in a standardized way using scales that demonstrate sufficiently robust measurement properties [4–9].

The goal of this study was to identify minimum standards for the selection of PRO measures for use in PCOR and CER. We defined minimum standards such that if a PRO measure did not meet these criteria, it would be judged not suitable for a PCOR study. A central aim in developing this set of standards was to clearly define the critical attributes for judging a PRO measure for a PCOR study. We identified these standards using two complementary approaches. The first was an extensive review of the literature including both published and unpublished guidance documents. The second was to seek input, via a formal survey, from an international group of experts in PRO measurement and PCOR who are members of the International Society for Quality of Life Research (ISOQOL) [10]. Although not the primary objective of this study, our approach allowed us to also identify criteria that were not deemed as a necessary minimum standard, but would rather be considered "best practice" standards for PRO measures.

Identification of minimal standards is a first step toward enabling PCOR and CER to achieve their goals of enhancing healthcare delivery and ultimately improving patients' health and well-being. Access to scientifically sound and decision-relevant PRO measures will allow investigators to collect empirical evidence on the differential benefits of interventions from the patients' perspective [6, 9, 11, 12]. This information can then be disseminated to patients, providers, and policy makers to provide a richer perspective on the impact of interventions on patients' lives using endpoints that are meaningful to them [13].

P. S. Hinds
Children's National Medical Center, Washington, DC, USA

P. S. Hinds
The George Washington University School of Medicine, Washington, DC, USA

R. D. Hays
David Geffen School of Medicine at UCLA, Los Angeles, CA, USA

R. Gershon · D. Cella · Z. Butt
Northwestern University Feinberg School of Medicine, Chicago, IL, USA

D. Feeny
University of Alberta, Alberta, Canada

P. M. Fayers
University of Aberdeen, Aberdeen, UK

P. M. Fayers
Norwegian University of Science and Technology (NTNU), Trondheim, Norway

M. Brundage
Queen's University, Kingston, ON, Canada

S. Ahmed
McGill University, Montreal, QC, Canada

N. K. Aaronson
The Netherlands Cancer Institute, Amsterdam, The Netherlands

N. K. Aaronson
University of Amsterdam, Amsterdam, The Netherlands

For peer review only - http://bmjopen.bmj.com/site/about/guidelines.xhtml

Methods

This paper is based on a study funded by the U.S. Patient-Centered Outcomes Research Institute (PCORI) [14]. The paper does not represent PCORI's Methodology Committee standards, issued separately by PCORI, though some of those standards were informed by this work [15]. An ISOQOL scientific advisory task force (SATF), consisting of the authors on this article, was set up to guide the drafting and final selection of recommended standards. We conducted a literature review that helped the SATF draft the recommendations that were subsequently reviewed by ISOQOL members in the formal survey. The literature review and the responses and feedback from ISOQOL members informed the final recommendations provided in this article.

Literature review

We conducted a systematic review of the published and unpublished literature to identify existing guidance documents related to PRO measures. The review identified current practices in selecting PRO measures in PCOR and CER, relevant scale attributes (e.g., reliability, validity, response burden, interpretability), and use of qualitative and quantitative methods to assess these properties. We focused on consensus statements, guidelines, and evidence-based papers, with an emphasis on articles or documents that described broadly generalizable principles. However, some papers that were population- or instrument-specific were included because of the rigor of the psychometric methods.

For the literature review, we adapted a published MEDLINE search strategy to identify measurement properties of PRO measures [16]. The published strategy was used as a foundation and adapted by using terms from MEDLINE thesaurus, Medical Subject Headings (MeSH), and the American Psychological Association's (APA) online Thesaurus of Psychological terms. We conducted parallel searches in several relevant electronic databases, including MEDLINE, PsycINFO, and Combined Index to Nursing and Allied Health Literature (CINAHL) (see database search terms in Appendix 1, ESM). There was no a priori restriction by publication date or age of sample. We also obtained relevant articles through a request to the ISOQOL membership email distribution list.

The titles and abstracts of identified articles and guidelines were reviewed by one of the authors (ZB). The full text of relevant articles was obtained and reviewed. The references cited in the selected articles were reviewed to identify additional relevant articles. ZB abstracted the necessary information for the study; two other authors (DC and RG) independently reviewed several of the articles to ensure coding consistency.

Based on PRO measurement standards gleaned from the literature review, the ISOQOL SATF drafted recommendations that were reviewed by ISOQOL members in a survey described below. Through an iterative series of SATF e-mails and conference calls, the potential standards identified by the systematic literature review were discussed and debated. Redundancies between potential standards were minimized, and similar items consolidated. Where there were differences in opinion among the members, different options were retained in the survey in order that the membership at large could rate and comment on each potential standard. The resultant survey consisted of 23 potential minimum standards to be rated by the ISOQOL membership.

Survey of ISOQOL membership

ISOQOL is dedicated to advancing the scientific study of HRQOL and other patient-centered outcomes to identify effective interventions, enhance the quality of healthcare and promote the health of populations [10]. Since 1993, ISOQOL has been an international collaborative network including researchers, clinicians, patient advocates, government scientists, industry representatives, and policy makers. Many ISOQOL members are PRO methodologists who focus on using state-of-the-art methods, both qualitative and quantitative, to improve the measurement and application of patient-reported data in research, healthcare delivery, and population surveillance. Many of the PRO measures widely used in research as well as the guidelines for developing and evaluating a PRO measure were developed by ISOQOL members. At the time of the survey, there were 506 ISOQOL members on the email distribution list.

In the web-based survey, we sought ISOQOL members' views on draft minimum standards, paying particular attention to areas where there did not appear to be consensus in the literature. For example, we asked ISOQOL members to rank the relative importance of various approaches for assessing reliability, including test–retest and internal consistency for multi-item PRO measures. In addition, we sought agreement on recommendations for six key attributes of PRO measures: (1) conceptual and measurement model, (2) reliability, (3) validity, (4) interpretability of scores, (5) translation, and (6) patient and investigator burden.

In the survey, it was deemed critical that respondents had a clear definition of a minimum standard. The second screen of the survey provided this guidance: "Please remember as you answer the questions in this survey that we are developing the minimum standards for the selection and design of a PRO measure for use in patient-centered outcomes research (PCOR). That is, we are saying a PRO measure that does not meet the minimum standard should not be considered appropriate for the research study." This statement was not intended to suggest that a PRO measure would not continue to be validated and strengthened as part of a maturation model of development. The survey directly mentioned PCOR, but the SATF believes these recommendations

For peer review only - http://bmjopen.bmj.com/site/about/guidelines.xhtml

are consistent for CER. For brevity, we use just "PCOR" in describing the results.

For each recommendation created by the SATF's synthesis of the literature review, the participant could select one of the following response options: required as a minimum standard, desirable but not required as a minimum standard, not required at all (not needed for a PRO measure), not sure, or no opinion. In analyzing the results, we used the general rule that if 50 % or more agreed that the recommendation was required as a minimum standard, then the recommendation was accepted. If less than 50 % of respondents were in agreement, then the recommendation was reviewed by the ISOQOL SATF to determine whether the recommendation may have been unclear or whether it would better be considered as a "best practice" (or "ideal standard") for PRO measures rather than a "minimum standard." Respondents were also encouraged to comment using a free text box that was provided after each recommendation. This text was extracted from the survey and helped inform the ISOQOL SATF's decisions and final recommendations.

The survey and a description of the survey methodology were submitted to the Institutional Review Board (IRB) at the University of North Carolina at Chapel Hill (UNC) for review and were determined to be exempt from IRB approval by the UNC Office of Human Research and Ethics. The online survey was designed and administered using the Qualtrics Software System under the UNC site license [17].

The survey link was sent out through the ISOQOL member email distribution list ($n = 506$) on 20 February , 2012. Survey instructions asked members to complete the survey within 9 days to meet deadlines for the PCORI contract. However, the response interval was extended to 20 March , 2012 (29 days), to accommodate more ISOQOL respondents. Information about the purpose of the voluntary survey, goals of the project, and funding source was included. All responses were anonymous, and no personal identifying information was collected. Two reminders were sent during the period the survey was available.

We did not expect responses from all ISOQOL members, because: (1) the survey was specifically aimed at those ISOQOL members who considered themselves to have the requisite expertise in the area of PRO measurement, and (2) we sought expert input in a short amount of time. Although we did not limit eligibility to those members who had such expertise, we did ask respondents to self-report their expertise level as part of the survey.

Results

Guidance identified through the literature review

A number of well-known guidance documents were identified, including guidance from the FDA [1, 18–20]; the 2002 Medical Outcomes Trust guidelines on attributes of a good HRQOL measure [2]; the extensive, international expert-driven recommendations from COSMIN (COnsensus-based Standards for the selection of health Measurement INstruments) [3, 4, 21–25]; the European Organization for Research and Treatment of Cancer (EORTC) guidelines for developing questionnaires [26]; the Functional Assessment of Chronic Illness Therapy (FACIT) approach [27]; the International Society for Pharmacoeconomics and Outcomes Research (ISPOR) task force recommendation documents [28–31]; the American Psychological Association (APA) Standards for Educational and Psychological Testing [32]; and several others [33–38]. We also had access to the recent standards documents just completed by the National Institutes of Health's Patient-Reported Outcomes Measurement Information System (PROMIS) network, which we considered useful for informing the minimal standards for PRO measures. In addition, ISOQOL recently completed two guidance documents relevant for this landscape review on the use of PRO measures in comparative effectiveness research and on integrating PRO measures in healthcare delivery settings [5, 39].

ISOQOL members identified a total of 301 additional references relevant for our task. Our formal search of the MEDLINE database yielded 821 references, which were individually reviewed, resulting in 60 additional relevant articles. Review of the 172 potentially relevant PsycINFO results provided 22 additional relevant articles, and an additional four unique references were uncovered after review of 126 abstracts identified through CINAHL.

Table 1 describes 28 key guidance documents identified from the literature review that helped to inform the ISOQOL SATF's draft minimum guidelines to be evaluated in the ISOQOL survey. The documents selected for further review and discussion by our ISOQOL SATF represented exemplar description of guidelines and standards for the selection of PRO in PCOR. As part of our literature review, we identified many more relevant references; however, our focus was on existing guidance documents that had broad relevance. Multiple publications describing the same set of guidelines were not cited separately.

Characteristics of participants responding to the ISOQOL survey

Table 2 summarizes the characteristics of the 120 ISOQOL members (23.7 %) who responded to the survey. Approximately 64 % of the sample had a PhD (or similar doctoral degree) and 18 % had a MD. The sample included 68 % academic researchers, 21 % clinicians, 8 % industry representatives, 23 % industry consultants, and 6 % federal government employees. There was diverse geographic distribution with 48 % of respondents from North America

For peer review only - http://bmjopen.bmj.com/site/about/guidelines.xhtml

Table 1 Identified guidelines for patient-reported outcomes measures

Author, year	Guideline	Research design	Description
Acquadro et al. [48]	The Literature review of methods to translate health-related quality of life questionnaires for use in multinational clinical trials	Formal literature review	Call for more empirical research on translation methodology; reviews several existing guidelines; advocates multistep process for translations
Cella [27]	Manual for the Functional Assessment of Chronic Illness Therapy (FACIT)	Description of method	Provides summary of FACIT scale development and translation methodologies; presents basic psychometric info for existing measures
Coons et al. [28]	Recommendations on evidence needed to support measurement equivalence between electronic and paper-based patient-reported outcome measures	Expert opinion and literature review	Provides a general framework for decisions regarding evidence needed to support migration of paper PRO measures to electronic delivery
COSMIN group, 2010 [24]	COSMIN study: COnsensus-based Standards for the selection of health Measurement INstruments	Guidelines established via systematic literature review and iterative Delphi process	Consensus was reached on design requirements and preferred statistical methods for the assessment of internal consistency, reliability, measurement error, content validity, construct validity, criterion validity, responsiveness, and interpretability
Crosby et al. [49]	Defining clinically meaningful change in health-related quality of life	Literature review	Reviews current approaches to defining clinically meaningful change in health-related quality of life and provides guidelines for their use
Dewolf et al. [36]	Translation procedure	Expert opinion	Provides guidance on the methodology for translating EORTC Quality of Life Questionnaires (QLQ)
Erickson et al. [19]	A concept taxonomy and an instrument hierarchy: tools for establishing and evaluating the conceptual framework of a patient-reported outcome (PRO) instrument as applied to product labeling claims	Expert opinion	Proposes a PRO concept taxonomy and instrument hierarchy that may be useful for demonstration of PRO measure claim for drug development, although they have not been tested for such purpose
Frost et al. [50]	What is sufficient evidence for the reliability and validity of patient-reported outcome measures?	Literature review	Article provides specific guidance on necessary psychometric properties of a PRO measure, with special reference to the FDA guidance, using the literature as a guide for specific statistical thresholds
Hays et al. [51]	The concept of clinically meaningful change in health-related quality of life research: How meaningful is it?	Expert opinion	Argues against a single threshold to define the minimally clinical important difference
Johnson et al. [26]	Guidelines for developing questionnaire modules	Expert opinion	Provides detailed description of PRO measure module development per the EORTC methodology related to generation of issues, construction of item list, pre- and field-testing
Kemmler et al. [52]	A new approach to combining clinical relevance and statistical significance for evaluation of quality of life changes in the individual patient	Longitudinal data from a chemotherapy trial	Data from this trial were used to evaluate change for individual participants (vs. groups). Stressed the importance of evaluation on the basis of statistical and clinical significance
Kottner et al. [53]	Guidelines for reporting reliability and agreement studies (GRRAS) were proposed	Literature review and expert consensus	Proposes a set of guidelines for reporting inter-rater agreement, inter-rater reliability in healthcare and medicine
Magasi et al. [33]	Content validity of patient-reported outcome measures: Perspectives from a PROMIS meeting	Expert presentation and discussion	The paper describes findings from a PROMIS meeting focused on content validity. Several recommendations were outlined as a result, including the need for consensus driven guidelines (none were proposed)

For peer review only - http://bmjopen.bmj.com/site/about/guidelines.xhtml

Table 1 continued

Author, year	Guideline	Research design	Description
Norquist et al. [42]	Choice of recall period for patient-reported outcome measures: criteria for consideration	Literature review	Choice of recall period for a PRO measure depends on nature of the disease, stability of symptoms, and trajectory of symptoms over time
Revicki et al. [12]	Recommendations on health-related quality of life research to support labeling and promotional claims in the United States	Review	Outlines the importance of an evidentiary base for making claims with respect to medical labeling or promotional claims
Revicki et al. [7]	Documenting the rationale and psychometric characteristics of patient-reported outcomes for labeling and promotional claims: the PRO Evidence Dossier	Report	Describes the purpose and content of a PRO measure Evidence Dossier, as well as its potential role with respect to regulatory review
Revicki et al. [34]	Recommended methods for determining responsiveness and minimally important differences for patient-reported outcomes	Literature review and expert opinion	Makes concrete recommendations regarding estimation of minimally important differences (MID), which should be based on patient-based and clinical anchors and convergence across multiple approaches and methods
Rothman et al. [30]	Use of existing patient-reported outcome (PRO) instruments and their modification	Expert opinion	Discusses key issues regarding the assessment and documentation of content validity for an existing instrument; discusses potential threats to content validity and methods to ameliorate
Schmidt et al. [54]	Current issues in cross-cultural quality of life instrument development	Literature review	Provides an overview of cross-cultural adaptation of PRO measure and provides broad development guidelines, as well as a call for additional focus on international research
Schunemann et al. [8]	Interpreting the results of patient-reported outcome measures in clinical trials: The clinician's perspective	Report based on examples	The authors provided several examples to describe how to attach meaning to PROM score thresholds and/or score differences
Scientific Advisory Committee of Medical Outcomes Trust [2]	Assessing health status and quality of life instruments: attributes and review criteria	Expert opinion	Describes 8 key attributes of PRO measures, including conceptual and measurement model, reliability, validity, responsiveness, interpretability, respondent and administrative burden, alternate forms, and cultural and language adaptations
Sprangers et al. [55]	Assessing meaningful change in quality of life over time: a users' guide for clinicians	Literature review and expert opinion	Proposes a set of guidelines/questions to help guide clinicians as to how to use PRO data in the treatment decision process
Snyder et al. [5]	Implementing patient-reported outcomes assessment in clinical practice: a review of the options and considerations	Literature review	The ISOQOL group developed a series of options and considerations to help guide the use of PROs in clinical practice, along with strengths and weaknesses of alternate approaches
Turner et al. [56]	Patient-reported outcomes: Instrument development and selection issues	Literature review	Provides a broad summary of concepts and issues to consider in the development and selection of a PRO measure
United States Food and Drug Administration [1]	Guidance for Industry: Patient-reported outcome measures: use in medical product development to support drug labeling claims	Expert opinion	"This guidance describes how the Food and Drug Administration (FDA) reviews and evaluates existing, modified, or newly created patient-reported outcome instruments used to support claims in approved medical product labeling." It covers conceptual frameworks, content validity, reliability, validity, ability to detect change, modification of PRO, and use of PRO in special populations

For peer review only - http://bmjopen.bmj.com/site/about/guidelines.xhtml

Table 1 continued

Author, year	Guideline	Research design	Description
Wild et al. [29]	Principles of good practice for the translation and cultural adaptation process for patient-reported outcomes measures	Literature review and expert opinion/ consensus	The ISPOR Task Force produced a critique of the strengths and weaknesses of various methods for translation and cultural adaptation of PROMS
Wild et al. [31]	Multinational trials-recommendations on the translations required, approaches to using the same language in different countries, and the approaches to support pooling the data	Expert opinion and literature review	Provides decision tools to decide on translation required for PRO measure; approach to use when same language is spoken in more than one country; and methods to gather evidence to support pooling of data across different language versions
Wyrwich et al. [38]	Methods for interpreting change over time in patient-reported outcome measures	Literature review	This article reviews the evolution of the methods and the terminology used to describe and aid in the communication of meaningful PRO change score thresholds

(86 % of these from the United States) and 33 % from Europe.

The participants reported being skilled in qualitative and quantitative methods and felt comfortable providing guidance for recommendations for PRO measurement standards. Approximately 81 % of the sample reported they had moderate to extensive training in quantitative methods and 53 % reported they had moderate to extensive training in qualitative methods. Overall, 89 % reported they felt competent or very competent providing guidance. As a sensitivity analysis, we examined the endorsement of recommendations excluding the 11 % who felt only somewhat or a little competent, but this resulted in no changes for our final recommendations. On average, the sample had 15 years of PRO measurement and research experience in the field.

Minimum standards for selecting a PRO measure for use in PCOR

Table 3 provides definitions of the properties of a PRO measure, and Table 4 provides an overview of the results from the ISOQOL survey on draft recommendations for minimal standards. Table 5 provides final recommendations based on these results and the feedback from ISOQOL members. A review of the findings from our literature review and survey is provided below.

Conceptual and measurement model

ISOQOL members were very supportive of the minimum standards described in Table 4 (#1) with 90 % of respondents endorsing the statement that a PRO measure should have documentation that defines the PRO construct and describes the intended application of the measure in the intended population. Also, 61 % of respondents agreed the documentation should describe how the measured concept(s) are operationalized in the measurement model.

Reliability of a PRO measure

A majority of ISOQOL respondents agreed that as a minimum standard a multi-item PRO measure should be assessed for internal consistency reliability, and a single-item PRO measure should be assessed by test–retest reliability (see Table 4, #2). However, they did not support as a minimum standard that a multi-item PRO measure should be required to have evidence of test–retest reliability. They noted practical concerns regarding test–retest reliability; primarily that some populations studied in PCOR are not stable and that their HRQOL can fluctuate. This phenomenon would reduce estimates of test–retest reliability, making the PRO measure look unreliable when it may be accurately detecting changes over time. In addition, memory effects will positively influence the test–retest reliability when the two survey points are scheduled close to each other.

Respondents endorsed the minimum level of reliability of 0.70 for group-level comparisons, which is commonly accepted in the field [2, 40, 41]. The standard error of measurement at this reliability level is approximately 0.55 of a standard deviation. However, there were concerns that establishing an absolute cut-off would be too prescriptive (e.g., a PRO measure with an estimated reliability coefficient of 0.69 would be deemed unreliable). Some respondents (36 %) supported the statement that "no minimum level of reliability should be stated; however, the reliability should be appropriately justified for the context of the proposed PRO measurement application."

For peer review only - http://bmjopen.bmj.com/site/about/guidelines.xhtml

Table 2 Participant-reported sample characteristics

Sample characteristic	% (n = 120)
Degrees[a]	
MD	18 %
PhD/Other Doctoral Degree (e.g., ScD)	64 %
RN/NP	5 %
Physical/Occupational Therapist	7 %
MA, MSc, MPH, or other Master's	43 %
Role[a]	
Academic Researcher	68 %
Clinician	21 %
Industry Representative	8 %
Industry Consultant/CRO Employee	23 %
Federal Government Employee	6 %
Patient Advocate	2 %
Other	8 %
Geographic location	
North America	48 %
United States	(86 %)
Europe	33 %
South America	5 %
Asia	10 %
Africa	1 %
Australia	3 %
Quantitative training in PRO measure design and evaluation	
Extensive training	37 %
Moderate amount of training	44 %
A little training	16 %
Not any training	3 %
Qualitative training in PRO measure design and evaluation	
Extensive training	18 %
Moderate amount of training	35 %
A little training	40 %
Not any training	7 %
Competency	
Very competent	50 %
Competent	39 %
Somewhat competent	8 %
A little competent	3 %
Average number of years in health-related quality (HRQOL) or patient-reported outcomes (PROs) field	
Mean years in HRQOL or PRO field	15 years; (range 1–40 years)

[a] More than one response was allowed for this characteristic

Validity of a PRO measure

The most common types of validity that were considered for minimum standards were content validity, construct validity, and responsiveness. Responsiveness is often regarded as an aspect of validity [4, 37]; however, it is often discussed separately given its importance to PRO measurement in longitudinal studies [4]. Criterion-related validity was not considered since there is generally no "gold standard" to which to compare a PRO measure. In the survey of ISOQOL members, only 7 and 10 % felt criterion-related validity was critical to have for a PRO measure in a cross-sectional or longitudinal study, respectively. It should be noted that the APA standards manual [32] suggests that validity is a unitary concept including all aspects of validity. However, the field of

For peer review only - http://bmjopen.bmj.com/site/about/guidelines.xhtml

Table 3 Definition of PRO measure properties

Conceptual and measurement model—The conceptual model provides a description and framework for the targeted construct(s) to be included in a PRO measure. The measurement model maps the individual items in the PRO measure to the construct
Reliability—The degree to which a PRO measure is free from measurement error [2, 4, 40, 41]
Internal consistency reliability—The degree of the interrelatedness among the items in a multi-item PRO measure [2, 4]
Test–retest reliability—A measure of the reproducibility of the scale, that is, the ability to provide consistent scores over time in a stable population [2]
Validity—The degree to which a PRO instrument measures the PRO concept it purports to measure [2, 4, 41]
Content validity—The extent to which the PRO measure includes the most relevant and important aspects of a concept in the context of a given measurement application [50]
Construct validity—The degree to which scores on the PRO measure relate to other measures (e.g., patient-reported or clinical indicators) in a manner that is consistent with theoretically derived a priori hypotheses concerning the concepts that are being measured [40]
Criterion validity—The degree to which the scores of a PRO measure are an adequate reflection of a "gold standard." [4]
Responsiveness—The extent to which a PRO measure can detect changes in the construct being measured over time [2, 37]
Interpretability of scores—The degree to which one can assign easily understood meaning to a PRO measure's scores [2, 4]
Minimal important difference (MID)—The smallest difference in score in the outcome of interest that informed patients or informed proxies perceive as important, either beneficial or harmful, and that would lead the patient or clinician to consider a change in the management [44, 57, 58]
Burden—The time, effort, and other demands placed on those to whom the instrument is administered (respondent burden) or on those who administer the instrument (investigator or administrative burden) [2]

For peer review only

outcomes research still distinguishes the above terms, probably because different methodologies are needed to address different forms of validity.

Content validity was rated as one of the most critical forms of validity to be assessed for a PRO measure with 58 and 61 % of ISOQOL members indicating a PRO measure must have evidence for content validity before using it in a cross-sectional or longitudinal study, respectively (data not shown in Table 4) [1]. Although the recommendations for minimum standards for content validity were endorsed by ISOQOL members (see Table 4, #3a), there was disagreement about the recall period, which is the period of time of reference (e.g., currently, past 24 h, past 7 days, past 4 weeks) for patients to describe their experiences with the measured PRO. Most (52 %) believed that a justification for the recall period was desirable but not required as a minimum standard for a PRO measure. In the final recommendation, we recommend that the reference period must be considered carefully in order for research participants to provide valid responses. However, we do not recommend a single recall period as it varies depending on the PRO domain being measured, the research context, and the population being studied [42].

Another aspect of content validity has to do with the provenance of items. One statement that was considered as a minimum standard but not supported by ISOQOL members was for the "documentation of sources from which items were derived, modified, and prioritized during the PRO measure development process." Because a majority of respondents felt this standard was important (46 % voted "required as minimum standard" and 46 % voted "desirable but not required"), we recommend this documentation be considered as a "best practice" but not a minimum standard for PRO measures.

Construct validity was also judged a critical component of validity. A majority of respondents (55 %) judged documentation of empirical findings supporting a priori hypotheses regarding expected associations among similar and dissimilar measures to be a minimal standard for a PRO measure (see Table 4, #3b). Another part of our original recommendation considered documented evidence for "known groups" validity, requiring empirical findings that support predefined hypotheses of the expected differences in scores between "known" groups. We considered this to be an important part of the evaluation of construct validity as it demonstrates the ability of a PRO measure to distinguish between one group and another where there is past empirical evidence of differences between the groups. However, the majority of ISOQOL members (57 %) rated it as a desirable but not required standard. Therefore, we considered this as a standard for "best practice" rather than a minimum standard.

Responsiveness, also referred to as longitudinal validity, is an aspect of construct validity [23, 37, 43]. A majority of ISOQOL respondents supported minimum standards of obtaining empirical evidence of changes in scores consistent with predefined hypotheses prior to using the PRO measure in longitudinal research (see Table 4, #3c). However, 65 % of respondents reported that they would use a PRO measure in a longitudinal study even if there was no prior study to support the responsiveness of the scale, but did have scientific evidence in a cross-sectional study of the reliability, content validity, and construct validity of the PRO measure.

For peer review only - http://bmjopen.bmj.com/site/about/guidelines.xhtml

Table 4 ISOQOL survey results on draft recommendations

	Draft recommendation for minimal standards	Survey results (n = 120)
1	Conceptual and measurement model	
	A PRO measure should have documentation defining and describing the concept(s) included and the intended population(s) for use	Required as a minimum standard—90 %<br>Desirable but not required as a minimum standard—9 %<br>Not required—0 %<br>Not sure—1 %<br>No opinion—0 %
	In addition, there should be documentation of how the concept(s) are organized into a measurement model, including evidence for the dimensionality of the measure, how items relate to each measured concept, and the relationship among concepts included in the PRO measure	Required as a minimum standard—61 %<br>Desirable but not required—35 %<br>Not required—3 %<br>Not sure—1 %<br>No opinion—0 %
2	Reliability	
	The reliability of a PRO measure should ideally be at or above 0.70 for group-level comparisons	Yes, it should be at or above 0.70—54 %<br>No, it should be at or above _fill in blank_—8 % (responses ranged from 0.50 to 0.80)<br>No minimum level of reliability should be appropriately justified for the context of the proposed application—36 %<br>No opinion—2 %
	Reliability for a multi-item unidimensional scale should include an assessment of internal consistency	Required as a minimum standard—79 %<br>Desirable but not required—14 %<br>Not required—2 %<br>Not sure—3 %<br>No opinion—2 %
	Reliability for a multi-item unidimensional scale should include an assessment of test–retest reliability	Required as a minimum standard—43 %<br>Desirable but not required—51 %<br>Not required—3 %<br>Not sure—3 %<br>No opinion—0 %
	Reliability for a single-item measure should be assessed by test–retest reliability	Required as a minimum standard—60 %<br>Desirable but not required—34 %<br>Not required—2 %<br>Not sure—3 %<br>No opinion—1 %
3	Validity	
3a	Content validity	
	A PRO measure should have evidence supporting its content validity, including evidence that patients and/or experts consider the content of the PRO measure relevant and comprehensive for the concept, population, and aim of the measurement application	Required as a minimum standard—78 %<br>Desirable but not required—19 %<br>Not required—2 %<br>Not sure—0 %<br>No opinion—1 %
	Documentation of qualitative and/or quantitative methods used to solicit and confirm attributes (i.e., concepts measured by the items) of the PRO relevant to the measurement application	Required as a minimum standard—53 %<br>Desirable but not required—44 %<br>Not required—2 %<br>Not sure—1 %<br>No opinion—0 %

For peer review only

For peer review only - http://bmjopen.bmj.com/site/about/guidelines.xhtml

Table 4 continued

	Draft recommendation for minimal standards	Survey results (n = 120)
	Documentation of the characteristics of participants included in the evaluation (e.g., race/ethnicity, culture, age, socio-economic status, literacy)	Required as a minimum standard—52 %<br>Desirable but not required—47 %<br>Not required—0 %<br>Not sure—0 %<br>No opinion—1 %
	Documentation of sources from which items were derived, modified, and prioritized during the PRO measure development process	Required as a minimum standard—46 %<br>Desirable but not required—46 %<br>Not required—7 %<br>Not sure—0 %<br>No opinion—1 %
	Justification for the recall period for the measurement application	Required as a minimum standard—41 %<br>Desirable but not required—52 %<br>Not required—5 %<br>Not sure—1 %<br>No opinion—1 %
3b	Construct validity	
	A PRO measure should have evidence supporting its construct validity, including documentation of empirical findings that support predefined hypotheses on the expected associations among measures similar or dissimilar to the measured PRO	Required as a minimum standard—55 %<br>Desirable but not required—44 %<br>Not required—1 %<br>Not sure—0 %<br>No opinion—0 %
	A PRO measure should have evidence supporting its construct validity, including documentation of empirical findings that support predefined hypotheses of the expected differences in scores between "known" groups	Required as a minimum standard—41 %<br>Desirable but not required—57 %<br>Not required—2 %<br>Not sure—0 %<br>No opinion—0 %
3c	Responsiveness	
	A PRO measure for use in longitudinal research study should have evidence of responsiveness, including empirical evidence of changes in scores consistent with predefined hypotheses regarding changes in the target population for the research application	Required as a minimum standard—57 %<br>Desirable but not required—42 %<br>Not required—1 %<br>Not sure—0 %<br>No opinion—0 %
	If a PRO measure has cross-sectional data that provide sufficient evidence in regard to the reliability (internal consistency), content validity, and construct validity but has no data yet on responsiveness over time (i.e., ability of a PRO measure to detect changes in the construct being measured over time), would you accept use of the PRO measure to provide valid data over time in a longitudinal study if no other PRO measure was available?	Yes—65 %<br>No, I would require evidence of responsiveness before accepting it—32 %<br>No opinion—0 %<br>Comments (fill in blank response)—22 %
4	Interpretability of Scores	
	A PRO measure should have documentation to support interpretation of scores, including what low and high scores represent for the measured concept	Required as a minimum standard—64 %<br>Desirable but not required—35 %<br>Not required—1 %<br>Not sure—0 %<br>No opinion—0 %
	A PRO measure should have documentation to support interpretation of scores, including representative mean(s) and standard deviation(s) in the reference population	Required as a minimum standard—39 %<br>Desirable but not required—57 %<br>Not required—4 %<br>Not sure—0 %<br>No opinion—0 %

For peer review only - http://bmjopen.bmj.com/site/about/guidelines.xhtml

Table 4 continued

	Draft recommendation for minimal standards	Survey results (n = 120)
	A PRO measure should have documentation to support interpretation of scores, including guidance on the minimally important difference in scores between groups and/or over time that can be considered meaningful from the patient and/or clinical perspective	Required as a minimum standard—23 %<br>Desirable but not required—72 %<br>Not required—5 %<br>Not sure—0 %<br>No opinion—0 %
5	Translation of a PRO measure	
	A PRO measure translated to one or more languages should have evidence of the equivalence of measurement properties for translated versions, allowing comparison or combination of data across language forms	Required as a minimum standard—47 %<br>Desirable but not required—49 %<br>Not required—4 %<br>Not sure—0 %<br>No opinion—0 %
	Documentation of background and experience of the persons involved in the translation	Required as a minimum standard—43 %<br>Desirable but not required—49 %<br>Not required—8 %<br>Not sure—0 %<br>No opinion—0 %
	Documentation of methods used to translate and evaluate the PRO measure in each language	Required as a minimum standard—81 %<br>Desirable but not required—16 %<br>Not required—3 %<br>Not sure—0 %<br>No opinion—0 %
	Documentation of extent of harmonization across different language versions	Required as a minimum standard—38 %<br>Desirable but not required—53 %<br>Not required—7 %<br>Not sure—2 %<br>No opinion—0 %
6	Patient and investigator Burden	
	The reading level of the PRO measure for research involving adult respondents from the general population should be at a minimum of...	4th grade education level—7 %<br>6th grade education level—23 %<br>8th grade education level—6 %<br>Other grade level ____—8 %<br>There should be no minimum requirement of the literacy level of the PRO measure; however, it should be appropriately justified for the context of it proposed application—43 %<br>Not sure—9 %<br>No opinion—4 %

For peer review only

Interpretability of scores

For a PRO measure to be well accepted for the use in PCOR, it must provide scores that are easily interpreted by different stakeholders including patients, clinicians, researchers, and policy makers [38]. The literature review revealed several ways to enhance interpretability of scores that may be considered for standard setting. End-users must be able to know what a high or low score represents. In addition, knowing what comprises a meaningful difference or change in the score from one group to another (or one time to another) would enhance understanding of the outcome being measured. Another way to enhance the interpretability of PRO measure scores would involve comparing scores from a study to known scores in a population (e.g., the general US population or a specific disease population). The availability of such benchmarks would enhance understanding of how the study group scored as compared to some reference or normative group.

A majority of respondents endorsed as a minimum standard that a PRO measure should have documentation to support the interpretation of scores including description of what low and high scores represent (see Table 4, #4). However, more useful metrics such as norm or reference

For peer review only - http://bmjopen.bmj.com/site/about/guidelines.xhtml

Table 5 Final recommendations for minimum standards for patient-reported outcome (PRO) measures used in patient-centered outcomes research or comparative effectiveness research

1	Conceptual and measurement model—A PRO measure should have documentation defining and describing the concept(s) included and the intended population(s) for use. In addition, there should be documentation of how the concept(s) are organized into a measurement model, including evidence for the dimensionality of the measure, how items relate to each measured concept, and the relationship among concepts included in the PRO measure
2	Reliability—The reliability of a PRO measure should preferably be at or above 0.70 for group-level comparisons, but may be lower if appropriately justified. Reliability can be estimated using a variety of methods including internal consistency reliability, test–retest reliability, or item response theory. Each method should be justified
3	Validity
3a	Content validity—A PRO measure should have evidence supporting its content validity, including evidence that patients and experts consider the content of the PRO measure relevant and comprehensive for the concept, population, and aim of the measurement application. This includes documentation of as follows: (1) qualitative and/or quantitative methods used to solicit and confirm attributes (i.e., concepts measured by the items) of the PRO relevant to the measurement application; (2) the characteristics of participants included in the evaluation (e.g., race/ethnicity, culture, age, gender, socio-economic status, literacy level) with an emphasis on similarities or differences with respect to the target population; and (3) justification for the recall period for the measurement application
3b	Construct validity—A PRO measure should have evidence supporting its construct validity, including documentation of empirical findings that support predefined hypotheses on the expected associations among measures similar or dissimilar to the measured PRO
3c	Responsiveness—A PRO measure for use in longitudinal research study should have evidence of responsiveness, including empirical evidence of changes in scores consistent with predefined hypotheses regarding changes in the measured PRO in the target population for the research application
4	Interpretability of scores—A PRO measure should have documentation to support interpretation of scores, including what low and high scores represent for the measured concept
5	Translation of the PRO measure—A PRO measure translated to one or more languages should have documentation of the methods used to translate and evaluate the PRO measure in each language. Studies should at least include evidence from qualitative methods (e.g., cognitive testing) to evaluate the translations
6	Patient and investigator Burden—A PRO measure must not be overly burdensome for patients or investigators. The length of the PRO measure should be considered in the context of other PRO measures included in the assessment, the frequency of PRO data collection, and the characteristics of the study population. The literacy demand of the items in the PRO measure should usually be at a 6th grade education level or lower (i.e., 12 year old or lower); however, it should be appropriately justified for the context of the proposed application

scores or minimally important difference (MID) estimates were not considered required, but were considered highly desirable [34, 44, 45].

Translation of a PRO measure

PCOR and CER are often carried out in multi-national or multi-cultural settings that require the PRO measure to be translated into different languages. To be able to compare or combine HRQOL results across those groups, it is critical that the measured HRQOL concept and the wording of the questionnaire used to measure it is interpreted in the same way across translations [29, 46].

Of the original draft recommendations reviewed in the survey (see Table 4, #5), ISOQOL members supported as a minimum standard the statement, “Documentation of methods used to translate and evaluate the PRO measure in each language.” In response to follow-up questions (not summarized in Table 4), 41 % of respondents considered it necessary, while 40 % felt it was expected but not required, to employ qualitative methods (e.g., cognitive interviews) for reviewing the quality of translations before using a translated PRO measure. Only 24 % of respondents thought that quantitative methods should be required for reviewing the quality of the translations (e.g., differential item functioning testing) before using the PRO measure, and 42 % of respondents indicated that it was expected (but not absolutely necessary) to include quantitative evaluation before they would use a translated PRO measure. Based on these findings, the ISOQOL SATF recommended that qualitative evidence be included as a minimum standard for translated PRO measures (Table 5).

Patient and investigator Burden

The committee agreed that burden on patients and investigators must be considered when selecting PRO measures for a PCOR study. A PRO measure must not be overly burdensome for patients as they are often ill and should not be subjected to overly long questionnaires or too frequent data collection that disrupts their lives. Ninety-two percent of the survey respondents concurred, endorsing “respondent burden” as an important or very important consideration for selecting PRO measures for PCOR.

Similarly, 90 % of respondents endorsed literacy as an important or very important consideration in selecting PRO measures in PCOR. Data collected from PRO measures are

For peer review only - http://bmjopen.bmj.com/site/about/guidelines.xhtml

only valid if the participants in a study can understand what is being asked of them and can provide a response that accurately reflects their experiences or perspectives. It is critical that developers of PRO measures ensure the questions, and response options are clear and easy to understand. Qualitative testing of the PRO measure (e.g., cognitive interviewing) should include individuals with low literacy to evaluate the questions [47]. Twenty-three percent of respondents indicated that a PRO measure should be written at 6th grade education level (ages 11–12 years), while 43 % indicated that the literacy level should be appropriately justified for the given research application.

Discussion

Based on a literature review of existing guidelines and a survey of experts in PRO measurement and research, we, on behalf of the ISOQOL, put forth minimum standards for PRO measures to be used in patient-centered outcomes research and comparative effectiveness research. These recommendations include the documentation of the characteristics of the conceptual and measurement model, evidence for reliability, validity, and interpretability of scores, quality translations, and acceptable patient and investigator burden (summarized in Table 5). The extent to which a PRO measure adheres to the standards described in this report reflects the quality of the PRO measurement.

Good documentation of the evidence that a PRO measure meets and exceeds these measurement properties will result in greater acceptance of the PRO measure for use in PCOR and CER. This documentation could include a focused methodologically rigorous study of the measurement properties of the PRO measure or analysis of HRQOL data collected from the PRO measure within a PCOR or CER study. Such documentation should be made available in peer-reviewed literature as well as on publically accessible websites. To the extent that the evidence was obtained from populations similar to the target population in the study, the investigator(s) will have greater confidence in the PRO measure to capture patients' experiences and perspectives.

There are a number of considerations when applying these minimum standards in PCOR and CER. The populations participating in PCOR and CER will likely be more heterogeneous than those that are typically included in phase II or III clinical trials. This population heterogeneity should be reflected in the samples included in the evaluation of the measurement properties for the PRO measure. For example, both qualitative and quantitative studies may require quota sampling based on race/ethnicity, gender, or age groups that reflect the prevalence of the condition in the study target population.

Researchers must consider carefully the strength of evidence supporting the measurement properties of the PRO measure. There is no threshold for which an instrument is valid or not valid for all populations or applications. In addition, no single study can confirm all the measurement properties for all research contexts. Like all scientific disciplines, measurement science relies on the iterative accumulation of a body of evidence (maturation model), replicated in different settings. Thus, it is the weight of the evidence (i.e., the number and quality of the studies and consistency of findings) that informs the evaluation of the appropriateness of a PRO measure. Older PRO measures will sometimes have the benefit of having more evidence than newer measures, and this will be reflected in the standards.

A possible limitation of this study is the potential for the biases of individual members of the SATF to influence the survey content. The transparency of the process used, and the wide variety of expertise and perspectives among the members, mitigated against substantive bias being introduced. In addition, the response rate to the survey was modest, again indicating the potential for bias. We point out, however, that the demographic data collected on the survey indicated that the respondents were experienced ISOQOL members with a variety of professional perspectives, the vast majority of whom self-identified as being competent in providing ratings and responses for the survey items.

These minimum standards were created by ISOQOL to reflect when a PRO measure may be considered appropriate or inappropriate for a specific PCOR study; thus, the intent was to have a minimum standard by which PRO measures could be judged acceptable. These standards do not reflect "ideal standards" or "best practices," which will have more stringent criteria [2, 3, 40]. For example, established minimally important differences for a PRO measure will enhance the interpretability of scores to inform decision making. As another example, establishing measurement equivalence of the PRO across different modes of assessment (e.g., paper forms, computers, handheld devices, phone) may facilitate broader patient participation in PCOR. ISOQOL's recommendations for "best practices" for PRO measures in PCOR and CER will be a next step in the organization's strategic initiative to advance the science of HRQOL measurement.

The findings from this study were reviewed by the PCORI Methodology Committee as part of that Committee's review of relevant standards and guidelines pertinent to patient-centered outcomes research. The ISOQOL recommendations presented here focus on more specific information about PRO measurement properties than those found in the PCORI Methodology Committee standards [15].

For peer review only - http://bmjopen.bmj.com/site/about/guidelines.xhtml

The identification and selection of PRO measures meeting and exceeding these current ISOQOL recommended minimum standards will increase the likelihood that the evidence generated in PCOR and CER reliably and validly represents the patients' perspective on health-related outcomes. This PRO evidence, based on instruments with sound measurement properties, can then be used to inform clinical and health policy decision making about the benefits and risks associated with different health interventions or to monitor population health.

Acknowledgments This study was funded by the Patient-Centered Outcomes Research Institute (PCORI-SOL-RMWG-001: PIs: Zeeshan Butt, PhD, Northwestern University; Bryce Reeve, PhD, University of North Carolina at Chapel Hill). The views expressed in this article are those of the authors and do not necessarily reflect those of PCORI.

References

1. US Food and Drug Administration. (2009). Patient-reported outcome measures: Use in medical product development to support labeling claims. Guidance for industry. http://www.fda.gov/downloads/Drugs/GuidanceComplianceRegulatoryInformation/Guidances/UCM071975.pdf. Accessed November 26, 2011.
2. Scientific Advisory Committee of the Medical Outcomes Trust. (2002). Assessing health status and quality-of-life instruments: Attributes and review criteria. Quality of Life Research, 11(3), 193–205.
3. Mokkink, L. B., Terwee, C. B., Knol, D. L., Stratford, P. W., Alonso, J., Patrick, D. L., et al. (2006). Protocol of the COSMIN study: COnsensus-based standards for the selection of health measurement INstruments. BMC Medical Research Methodology, 6, 2. doi:10.1186/1471-2288-6-2.
4. Mokkink, L. B., Terwee, C. B., Patrick, D. L., Alonso, J., Stratford, P. W., Knol, D. L., et al. (2010). The COSMIN study reached international consensus on taxonomy, terminology, and definitions of measurement properties for health-related patient-reported outcomes. Journal of Clinical Epidemiology, 63(7), 737–745. doi:10.1016/j.jclinepi.2010.02.006.
5. Snyder, C. F., Aaronson, N. K., Choucair, A. K., Elliott, T. E., Greenhalgh, J., Halyard, M. Y., et al. (2011). Implementing patient-reported outcomes assessment in clinical practice: A review of the options and considerations. Quality of Life Research,. doi:10.1007/s11136-011-0054-x.
6. Basch, E. M., Reeve, B. B., Mitchell, S. A., Clauser, S. B., Minasian, L., Sit, L., et al. (2011). Electronic toxicity monitoring and patient-reported outcomes. Cancer Journal, 17(4), 231–234. doi:10.1097/PPO.0b013e31822c28b3.
7. Revicki, D. A., Gnanasakthy, A., & Weinfurt, K. (2007). Documenting the rationale and psychometric characteristics of patient reported outcomes for labeling and promotional claims: The PRO Evidence Dossier. Quality of Life Research, 16(4), 717–723. doi:10.1007/s11136-006-9153-5.
8. Schunemann, H. J., Akl, E. A., & Guyatt, G. H. (2006). Interpreting the results of patient reported outcome measures in clinical trials: The clinician's perspective. Health and Quality of Life Outcomes, 4, 62. doi:10.1186/1477-7525-4-62.
9. Deyo, R. A., & Patrick, D. L. (1989). Barriers to the use of health status measures in clinical investigation, patient care, and policy research. Medical Care, 27(3 Suppl), S254–S268.
10. International Society for Quality of Life Research. http://www.isoqol.org/. Accessed July 30, 2012.
11. Guyatt, G., & Schunemann, H. (2007). How can quality of life researchers make their work more useful to health workers and their patients? Quality of Life Research, 16(7), 1097–1105. doi:10.1007/s11136-007-9223-3.
12. Revicki, D. A., Osoba, D., Fairclough, D., Barofsky, I., Berzon, R., Leidy, N. K., et al. (2000). Recommendations on health-related quality of life research to support labeling and promotional claims in the United States. Quality of Life Research, 9(8), 887–900.
13. Lipscomb, J., Donaldson, M. S., Arora, N. K., Brown, M. L., Clauser, S. B., Potosky, A. L., et al. (2004). Cancer outcomes research. Journal of the National Cancer Institute Monographs (33), 178–197. doi:10.1093/jncimonographs/lgh039.
14. US Patient-Centered Outcomes Research Institute http://www.pcori.org. Accessed 26 November, 2011.
15. Methodology Committee of the Patient-Centered Outcomes Research, I. (2012). Methodological standards and patient-centeredness in comparative effectiveness research: The PCORI perspective. Journal of the American Medical Association, 307(15), 1636–1640. doi:10.1001/jama.2012.466.
16. Terwee, C. B., Jansma, E. P., Riphagen, I. I., & de Vet, H. C. (2009). Development of a methodological PubMed search filter for finding studies on measurement properties of measurement instruments. Quality of Life Research, 18(8), 1115–1123. doi:10.1007/s11136-009-9528-5.
17. Qualtrics Labs Inc. Why choose qualtrics survey software? https://www.qualtrics.com/why-survey-software. Accessed November 26, 2011.
18. US Food and Drug Administration. (2010). Qualification process for drug development tools. Draft Guidance for Industry. http://www.fda.gov/downloads/Drugs/GuidanceComplianceRegulatoryInformation/Guidances/UCM230597.pdf. Accessed November 26, 2011.
19. Erickson, P., Willke, R., & Burke, L. (2009). A concept taxonomy and an instrument hierarchy: Tools for establishing and evaluating the conceptual framework of a patient-reported outcome (PRO) instrument as applied to product labeling claims. Value in Health, 12(8), 1158–1167. doi:10.1111/j.1524-4733.2009.00609.x.
20. Patrick, D. L., Burke, L. B., Powers, J. H., Scott, J. A., Rock, E. P., Dawisha, S., et al. (2007). Patient-reported outcomes to support medical product labeling claims: FDA perspective. Value in Health, 10(Suppl 2), S125–S137. doi:10.1111/j.1524-4733.2007.00275.x.
21. Angst, F. (2011). The new COSMIN guidelines confront traditional concepts of responsiveness. BMC Medical Research Methodology, 11, 152; author reply 152. doi:10.1186/1471-2288-11-152.
22. Mokkink, L. B., Terwee, C. B., Gibbons, E., Stratford, P. W., Alonso, J., Patrick, D. L., et al. (2010). Inter-rater agreement and reliability of the COSMIN (COnsensus-based standards for the selection of health status measurement instruments) checklist. BMC Medical Research Methodology, 10, 82. doi:10.1186/1471-2288-10-82.
23. Mokkink, L. B., Terwee, C. B., Knol, D. L., Stratford, P. W., Alonso, J., Patrick, D. L., et al. (2010). The COSMIN checklist for evaluating the methodological quality of studies on measurement properties: A clarification of its content. BMC Medical Research Methodology, 10, 22. doi:10.1186/1471-2288-10-22.
24. Mokkink, L. B., Terwee, C. B., Patrick, D. L., Alonso, J., Stratford, P. W., Knol, D. L., et al. (2010). The COSMIN checklist for assessing the methodological quality of studies on measurement properties of health status measurement instruments: An international Delphi study. Quality of Life Research, 19(4), 539–549. doi:10.1007/s11136-010-9606-8.
25. Terwee, C. B., Mokkink, L. B., Knol, D. L., Ostelo, R. W., Bouter, L. M., & de Vet, H. C. (2012). Rating the methodological

For peer review only - http://bmjopen.bmj.com/site/about/guidelines.xhtml

quality in systematic reviews of studies on measurement properties: A scoring system for the COSMIN checklist. Quality of Life Research, 21(4), 651–657. doi:10.1007/s11136-011-9960-1.
26. Johnson, C., Aaronson, N., Blazeby, J. M., Bottomley, A., Fayers, P., Koller, M., et al. (2011). EORTC Quality of life group: Guidelines for developing questionnaire modules. http://groups.eortc.be/qol/sites/default/files/archives/guidelines_for_developing_questionnaire-_final.pdf. Accessed November 26, 2011.
27. Cella, D. (1997). Manual of the functional assessment of chronic illness therapy (FACIT) measurement system. Evanston, IL: Northwestern University.
28. Coons, S. J., Gwaltney, C. J., Hays, R. D., Lundy, J. J., Sloan, J. A., Revicki, D. A., et al. (2009). Recommendations on evidence needed to support measurement equivalence between electronic and paper-based patient-reported outcome (PRO) measures: ISPOR ePRO good research practices task force report. Value in Health, 12(4), 419–429. doi:10.1111/j.1524-4733.2008.00470.x.
29. Wild, D., Grove, A., Martin, M., Eremenco, S., McElroy, S., Verjee-Lorenz, A., et al. (2005). Principles of good practice for the translation and cultural adaptation process for patient-reported outcomes (PRO) measures: Report of the ISPOR task force for translation and cultural adaptation. Value in Health, 8(2), 94–104. doi:10.1111/j.1524-4733.2005.04054.x.
30. Rothman, M., Burke, L., Erickson, P., Leidy, N. K., Patrick, D. L., & Petrie, C. D. (2009). Use of existing patient-reported outcome (PRO) instruments and their modification: The ISPOR good research practices for evaluating and documenting content validity for the use of existing instruments and their modification PRO task force report. Value in Health, 12(8), 1075–1083. doi:10.1111/j.1524-4733.2009.00603.x.
31. Wild, D., Eremenco, S., Mear, I., Martin, M., Houchin, C., Gawlicki, M., et al. (2009). Multinational trials-recommendations on the translations required, approaches to using the same language in different countries, and the approaches to support pooling the data: the ISPOR patient-reported outcomes translation and linguistic validation good research practices task force report. Value in Health, 12(4), 430–440. doi:10.1111/j.1524-4733.2008.00471.x.
32. Joint Committee on Standards for Educational and Psychological Testing of the American Educational Research Association, American Psychological Association, & National Council on Measurement in Education (1998). Standards for educational and psychological testing Washington DC: American Psychological Association.
33. Magasi, S., Ryan, G., Revicki, D., Lenderking, W., Hays, R. D., Brod, M., et al. (2011). Content validity of patient-reported outcome measures: Perspectives from a PROMIS meeting. Quality of Life Research,. doi:10.1007/s11136-011-9990-8.
34. Revicki, D., Hays, R. D., Cella, D., & Sloan, J. (2008). Recommended methods for determining responsiveness and minimally important differences for patient-reported outcomes. Journal of Clinical Epidemiology, 61(2), 102–109. doi:10.1016/j.jclinepi.2007.03.012.
35. Valderas, J. M., Ferrer, M., Mendivil, J., Garin, O., Rajmil, L., Herdman, M., et al. (2008). Development of EMPRO: A tool for the standardized assessment of patient-reported outcome measures. Value in Health, 11(4), 700–708. doi:10.1111/j.1524-4733.2007.00309.x.
36. Dewolf, L., Koller, M., Velikova, G., Johnson, C., Scott, N., & Bottomley, A. (2009). EORTC quality of life group: Translation procedure. http://groups.eortc.be/qol/sites/default/files/archives/translation_manual_2009.pdf. Accessed November 26, 2011.
37. Hays, R. D., & Hadorn, D. (1992). Responsiveness to change: An aspect of validity, not a separate dimension. Quality of Life Research, 1(1), 73–75.
38. Wyrwich, K. W., Norquist, J. M., Lenderking, W. R., Acaster, S., & the Industry Advisory Committee of International Society for Quality of Life, R. (2012). Methods for interpreting change over time in patient-reported outcome measures. Quality of Life Research, 2012 Apr 17. [Epub ahead of print.]. doi:10.1007/s11136-012-0175-x.
39. Ahmed, S., Berzon, R. A., Revicki, D., Lenderking, W., Moinpour, C. M., Basch, E., et al. (2012). The use of patient-reported outcomes (PRO) within comparative effectivenss research: Implications for clinical practice and healthcare policy. Medical Care, 50(12), 1060–1070.
40. Terwee, C. B., Bot, S. D., de Boer, M. R., van der Windt, D. A., Knol, D. L., Dekker, J., et al. (2007). Quality criteria were proposed for measurement properties of health status questionnaires. Journal of Clinical Epidemiology, 60(1), 34–42. doi:10.1016/j.jclinepi.2006.03.012.
41. Nunnally, J. C., & Berstein, I. H. (1994). Psychometric theory (3rd ed.). New York: McGraw-Hill.
42. Norquist, J. M., Girman, C., Fehnel, S., Demuro-Mercon, C., & Santanello, N. (2011). Choice of recall period for patient-reported outcome (PRO) measures: Criteria for consideration. Quality of Life Research,. doi:10.1007/s11136-011-0003-8.
43. Revicki, D. A., Cella, D., Hays, R. D., Sloan, J. A., Lenderking, W. R., & Aaronson, N. K. (2006). Responsiveness and minimal important differences for patient reported outcomes. Health and Quality of Life Outcomes, 4, 70. doi:10.1186/1477-7525-4-70.
44. Brozek, J. L., Guyatt, G. H., & Schunemann, H. J. (2006). How a well-grounded minimal important difference can enhance transparency of labelling claims and improve interpretation of a patient reported outcome measure. Health and Quality of Life Outcomes, 4, 69. doi:10.1186/1477-7525-4-69.
45. Norman, G. R., Sridhar, F. G., Guyatt, G. H., & Walter, S. D. (2001). Relation of distribution- and anchor-based approaches in interpretation of changes in health-related quality of life. Medical Care, 39(10), 1039–1047.
46. Koller, M., Kantzer, V., Mear, I., Zarzar, K., Martin, M., Greimel, E., et al. (2012). The process of reconciliation: Evaluation of guidelines for translating quality-of-life questionnaires. Expert Review of Pharmacoeconomics & Outcomes Research, 12(2), 189–197. doi:10.1586/erp.11.102.
47. Jordan, J. E., Osborne, R. H., & Buchbinder, R. (2011). Critical appraisal of health literacy indices revealed variable underlying constructs, narrow content and psychometric weaknesses. Journal of Clinical Epidemiology, 64(4), 366–379. doi:10.1016/j.jclinepi.2010.04.005.
48. Acquadro, C., Conway, K., Hareendran, A., & Aaronson, N. (2008). Literature review of methods to translate health-related quality of life questionnaires for use in multinational clinical trials. Value in Health, 11(3), 509–521. doi:10.1111/j.1524-4733.2007.00292.x.
49. Crosby, R. D., Kolotkin, R. L., & Williams, G. R. (2003). Defining clinically meaningful change in health-related quality of life. Journal of Clinical Epidemiology, 56(5), 395–407.
50. Frost, M. H., Reeve, B. B., Liepa, A. M., Stauffer, J. W., Hays, R. D., & Mayo, F. D. A. P.-R. O. C. M. G. (2007). What is sufficient evidence for the reliability and validity of patient-reported outcome measures? Value in Health, 10(Suppl 2), S94–S105. doi:10.1111/j.1524-4733.2007.00272.x.
51. Hays, R. D., & Woolley, J. M. (2000). The concept of clinically meaningful difference in health-related quality-of-life research. How meaningful is it? PharmacoEconomics, 18(5), 419–423.
52. Kemmler, G., Zabernigg, A., Gattringer, K., Rumpold, G., Giesinger, J., Sperner-Unterweger, B., et al. (2010). A new approach to combining clinical relevance and statistical significance for evaluation of quality of life changes in the individual patient. Journal of Clinical Epidemiology, 63(2), 171–179. doi:10.1016/j.jclinepi.2009.03.016.
53. Kottner, J., Audige, L., Brorson, S., Donner, A., Gajewski, B. J., Hrobjartsson, A., et al. (2011). Guidelines for reporting reliability

For peer review only - http://bmjopen.bmj.com/site/about/guidelines.xhtml

and agreement studies (GRRAS) were proposed. *Journal of Clinical Epidemiology*, 64(1), 96–106. doi:10.1016/j.jclinepi.2010.03.002.

54. Schmidt, S., & Bullinger, M. (2003). Current issues in cross-cultural quality of life instrument development. *Archives of Physical Medicine and Rehabilitation*, 84(4 Suppl 2), S29–S34. doi:10.1053/apmr.2003.50244.
55. Sprangers, M. A., Moinpour, C. M., Moynihan, T. J., Patrick, D. L., Revicki, D. A., & Clinical Significance Consensus Meeting Group. (2002). Assessing meaningful change in quality of life over time: A users' guide for clinicians. *Mayo Clinic Proceedings*, 77(6), 561–571. doi:10.4065/77.6.561.
56. Turner, R. R., Quittner, A. L., Parasuraman, B. M., Kallich, J. D., Cleeland, C. S., & Mayo, F. D. A. P.-R. O. C. M. G. (2007). Patient-reported outcomes: Instrument development and selection issues. *Value in Health*, 10(Suppl 2), S86–S93. doi:10.1111/j.1524-4733.2007.00271.x.
57. Schunemann, H. J., & Guyatt, G. H. (2005). Commentary–goodbye M(C)ID! Hello MID, where do you come from? *Health Services Research*, 40(2), 593–597. doi:10.1111/j.1475-6773.2005.00374.x.
58. Schunemann, H. J., Puhan, M., Goldstein, R., Jaeschke, R., & Guyatt, G. H. (2005). Measurement properties and interpretability of the Chronic respiratory disease questionnaire (CRQ). *Copd*, 2(1), 81–89.

For peer review only

For peer review only - http://bmjopen.bmj.com/site/about/guidelines.xhtml

BMJ Open

Development and validation of the Multimorbidity Treatment Burden Questionnaire (MTBQ)

Journal:	*BMJ Open*
Manuscript ID	bmjopen-2017-019413.R1
Article Type:	Research
Date Submitted by the Author:	19-Jan-2018

Complete List of Authors:	Duncan, Polly; University of Bristol, Centre for Academic Primary Care<br>Murphy, Mairead; University of Bristol, Centre for Academic Primary Care<br>Man, Mei-See; University of Bristol School of Social and Community Medicine,<br>Chaplin, Katherine ; University of Bristol School of Social and Community Medicine<br>Gaunt, Daisy; University of Bristol Faculty of Medicine and Dentistry, Bristol Randomised Trials Collaboration & School of Social and Community Medicine<br>Salisbury, Chris; University of Bristol, Academic Unit of Primary Health Care

**Primary Subject Heading**:	General practice / Family practice
Secondary Subject Heading:	Evidence based practice, Geriatric medicine, Health services research, Patient-centred medicine
Keywords:	Treatment burden, Multimorbidity, Patient reported outcome measure, Questionnaire, PRIMARY CARE

SCHOLARONE™
Manuscripts

Title **Development and validation of the Multimorbidity Treatment Burden Questionnaire (MTBQ)**

Authors
Dr Polly Duncan
GP and NIHR In-Practice Fellow
University of Bristol

Dr Mairead Murphy
Senior Research Associate
University of Bristol

Dr Mei-See Man
Trial Manager
University of Bristol

Dr Katherine Chaplin
Senior Research Associate
University of Bristol

Miss Daisy Gaunt
Senior Research Associate in Medical Statistics
University of Bristol

Prof Chris Salisbury
Professor of Primary Health Care
University of Bristol

Corresponding author
Dr Polly Duncan
Room 1.07, Centre for Academic Primary Care,
University of Bristol
Canynge Hall
39 Whatley Road
Bristol, BS8 2PS
01173313903
polly.duncan@bristol.ac.uk

Support National Institute for Health Research funding

Prior presentations Duncan P, ‘Development and validation of the Multimorbidity Treatment Burden Questionnaire’, oral presentation at the Annual Society for Academic Primary Care Conference, Warwick, UK, 12th July 2017.

Word count Abstract 296 words, main article 4124 words

Numbers of

	Tables	5
	Figures	0
	Appendices	5

For peer review only - http://bmjopen.bmj.com/site/about/guidelines.xhtml

Abstract

Objective: To develop and validate a new scale to assess treatment burden (the effort of looking after one's health) for patients with multimorbidity.

Methods: Design: mixed-methods

Setting: UK primary care

Participants: Content of the Multimorbidity Treatment Burden Questionnaire (MTBQ) was based on a literature review and views from a patient and public involvement group. Face validity was assessed through cognitive interviews. The scale was piloted and the final version was tested in 1546 adults with multimorbidity (mean age 71 years) who took part in the 3D Study, a cluster randomised controlled trial.

For each question, we examined the proportion of missing data and the distribution of responses. Factor analysis, Cronbach's alpha, Spearman's rank correlations and longitudinal regression assessed dimensional structure, internal consistency reliability, construct validity and responsiveness respectively. We assessed interpretability by grouping the global MTBQ scores into zero and tertiles (>0) and comparing participant characteristics across these categories.

Results: Cognitive interviews found good acceptability and content validity. Factor analysis supported a one-factor solution. Cronbach's alpha was 0.83, indicating internal consistency reliability. The MTBQ score had a positive association with a comparator treatment burden scale (Rs 0.58, $p<0.0001$) and with self-reported disease burden (Rs 0.43, $p<0.0001$) and a negative association with quality of life (Rs -0.36, $p<0.0001$) and self-rated health (Rs -0.36, $p<0.0001$). Female participants, younger participants and participants with mental health conditions were more likely to have high treatment burden scores. Changes in MTBQ score over nine-month follow-up were associated, as expected, with changes in measures of quality of life (EQ-5D-5L) and patient-centred care (PACIC).

Conclusion: The MTBQ is a ten-item measure of treatment burden for patients with multimorbidity that has demonstrated good content validity, construct validity, reliability and responsiveness. It is a useful research tool for assessing the impact of interventions on treatment burden.

Key words: Treatment burden, multimorbidity, patient reported outcome measure, questionnaire, primary care

For peer review only - http://bmjopen.bmj.com/site/about/guidelines.xhtml

**Abbreviations:**	MTBQ	Multimorbidity treatment burden questionnaire
	PROM	Patient reported outcome measure
	HCTD	Health Care Task Difficulty questionnaire
	TBQ	Treatment Burden Questionnaire
	PETS	Patient Experience with Treatment and Self-management questionnaire
	MULTIPLes	Multimorbidity Illness Perceptions Scale
	EQ-5D-5L	EuroQol five dimensions, five level questionnaire
	PACIC	Patients Assessment Chronic Illness Care

For peer review only

For peer review only - http://bmjopen.bmj.com/site/about/guidelines.xhtml

Article Summary

Strengths and limitations of the study

- A concise simply worded measure based on an evidence-based framework to include all the important aspects of treatment burden
- The measure was comprehensively tested using international standards for validating questionnaires
- Validated in 1546 mostly elderly patients with three or more long-term conditions
- Study participants were recruited into a trial, which may limit generalisability
- High floor effects were found similar to other existing treatment burden questionnaires

For peer review only

For peer review only - http://bmjopen.bmj.com/site/about/guidelines.xhtml

Introduction

Treatment burden is a patient's perception of the effort required to self-manage their medical conditions and the impact that this has on their general wellbeing.[1] This includes complex medication regimens, co-ordinating health care appointments, making lifestyle changes, and self-monitoring.

This is particularly relevant to patients with multimorbidity (having multiple long-term conditions). Associated with the ageing population, multimorbidity has become the norm, affecting over two-thirds of adults attending general practice.[2] Current health policy envisages greater support for patients to self-manage their chronic medical conditions. However, the time and energy this requires of patients can be overwhelming.[3]

In order to understand the impact of treatment burden, and particularly to assess the effects of interventions which might increase or decrease burden, a valid patient reported outcome measure (PROM) is essential. There are four existing PROMs that measure aspects of treatment burden for patients with multimorbidity,[4-8] all of which have important limitations. The 13-question Treatment Burden Questionnaire (TBQ) was originally developed in French, and subsequently a revised 15-question English version was tested.[4 5] Some of the content is healthcare system specific and the wording is relatively complex, perhaps reflecting the fact that the English version was tested in a relatively young and highly educated population of volunteers recruited from the 'Patients like me' website (mean age 51 years, 78% with college education), not all of whom had multimorbidity.[4] The Patient Experience with Treatment and Self-management (PETS) PROM was recently developed in the United States and includes 48 questions grouped under nine separate domains of treatment burden.[8] Whilst this measure is comprehensive, its length is a limitation. The Multimorbidity Illness Perceptions Scale (MULTIPLes) was developed and validated in elderly patients (mean age 70 years) with multimorbidity and includes a six-question Treatment Burden Subscale and a three-question Activity Limitation subscale.[7] This measure is brief but omits several important aspects of treatment burden. Similarly, the 11-question Healthcare Task Difficulty (HCTD) questionnaire was designed to measure only one aspect of treatment burden.[6]

The purpose of this study was to develop and validate a new concise measure of treatment burden for patients with multimorbidity.

For peer review only

For peer review only - http://bmjopen.bmj.com/site/about/guidelines.xhtml

Methods

Study Setting

This questionnaire was developed and validated as part of the 3D Study, a multicentre cluster-randomised control trial that aims to improve the management of patients with multimorbidity within primary care.[9] Participants aged 18 years or older with three or more of the long-term conditions included in the 2014 UK Quality and Outcomes Framework were recruited from 33 general practices in three areas of the UK.

Development of the questionnaire

We reviewed the literature on the concept and measurement of treatment burden in multimorbidity using Pubmed in July 2014. We identified a number of relevant qualitative studies[10-12] and three relevant existing PROMs that were not specific to a particular medical condition. These were the Treatment Burden Questionnaire (TBQ),[4 5] the Multimorbidity Illness Perceptions Scale (MULTIPLes)[7] and the Healthcare Task Difficulty (HCTD) questionnaire.[6] A further measure, the PETS scale, was published later.[8] We identified relevant domains for the PROM by reviewing the three existing PROMs against a framework of treatment burden which had been developed following qualitative interviews and focus groups.[1] We then sought the views from a Patient and Public Involvement (PPI) group of eight patients with multimorbidity formed for the purpose of the 3D Study, discussing the concept of treatment burden, the existing measures, the treatment burden framework and the domains of treatment burden to be included in the questionnaire. We then developed a draft questionnaire with 12 questions and undertook two rounds of cognitive interviews with eight PPI group members to improve the face and content validity of the scale (Appendix A).[13] Participants were asked to "think aloud"[13] as they completed the questionnaire commenting on the reasoning behind their ratings; perceived question meaning, the layout, title, introduction and general wording. They also gave their own examples of treatment burden and reflected on whether these would be captured by the questionnaire. Modifications to the questionnaire were made between the two rounds and an additional question was added about accessing health care during the evenings and weekends (see results). Following written consent, the interviews were audio-taped and field notes were taken. The second round of cognitive interviews led to only minor changes to the questionnaire with no new insights emerging. A debriefing meeting was held with PPI members and final changes to the 13-question questionnaire were made.

Recruitment, data collection and measures

Data were collected in two related studies, the cross-sectional 3D pilot study, and the longitudinal main 3D study, a cluster randomised controlled trial. The 13 candidate questions were included in a questionnaire, named the Multimorbidity Treatment Burden Questionnaire (MTBQ). Socio-demographic information (see Table 1) was collected at baseline in both the pilot and main studies. Details of participant's medical conditions were collected from their family practice computer records. Measures of health-related quality of life (EQ-5D-5L),[14] self-rated health (single question item), self-reported disease burden (Bayliss)[15] and patient-centred care

For peer review only - http://bmjopen.bmj.com/site/about/guidelines.xhtml

(PACIC)[16] were collected at baseline and nine months in both the pilot and main 3D studies. Following a review of existing measures and discussion with the PPI group, the Health Care Task Difficulty (HCTD)[6] questionnaire was included in the pilot study questionnaire as the best comparator for the MTBQ. A key reason for choosing this measure was the simple wording and brevity. This was felt to be important because many of the participants of the 3D study were older people and some had low literacy levels.

The questionnaire was sent to participants by post. For non-responders, a reminder letter was sent 10-14 days later, and a second reminder phone call was made 10-14 days after this.

Analysis

Data were analysed using STATA (Version 14). We generated descriptive statistics of participant characteristics for the pilot and main studies. The pilot study data were used to test the pre-specified hypothesis of a positive association between global MTBQ score and HCTD score. The main study data were used for the remainder of the analysis.

We tested the psychometric properties of the questionnaire against the minimum standards set out by the International Society for Quality of Life Research (ISOQOL).[17] The analysis plan and results are described in relation to ISOQOL's six recommended standards.

1. <u>Conceptual and measurement model</u>

1a. Conceptual framework

See 'Development of the questionnaire'.

1b. Question properties

To assess the properties of the questions, we examined the proportion of missing data and 'does not apply' responses and the distribution of responses. Responses of 'not difficult' or 'does not apply' were scored as zero. Floor and ceiling effects of the MTBQ were compared with the HCTD.[6] Questions with a proportion of 'does not apply' responses greater than 40% were removed and excluded from the analysis.

1c. Dimensionality

To examine the dimensionality of the scale, we performed factor analysis. This is a statistical technique used to reduce a larger number of items into a smaller number of common factors that reflect shared variance.[18] Items which share a lot of variance should have high "loadings" (correlation between the item and the factor), and low uniqueness (variance which is unique to the item, not common to the factor).

For peer review only - http://bmjopen.bmj.com/site/about/guidelines.xhtml

Loading of at least 0.4 and uniqueness of less than 0.6 are acceptable[19]. The number of factors extracted was decided by a combination of Kaiser's rule (eigenvalues greater than one),[20] the scree plot,[18] and by interpretability of domains.

2. Reliability

To test internal consistency reliability, we examined the inter-item correlation matrix and calculated Cronbach's alpha, a measure of consistency between the items in a scale. Inter-item correlations between 0.2 and 0.4 were deemed ideal.[21] A Cronbach's alpha of 0.7-0.9 was acceptable.[22]

3. Validity

3a. Content validity

The content validity of the questionnaire was tested iteratively using cognitive interviews (see 'Development of the questionnaire').

3b. Construct validity

Each question was scored as follows: zero (not difficult/ does not apply), one (a little difficult), two (quite difficult), three (very difficult), four (extremely difficult). Participants were excluded if more than 50% of their responses were missing. To calculate a global score, each participant's average score was calculated from the questions answered and multiplied by 25 to give a score from 0-100.

Construct validity was examined by testing five pre-specified hypotheses: first, a positive association between global MTBQ score and global HCTD score;[6] second, a negative association between global MTBQ score and health-related quality of life (EQ-5D-5L);[14] third, a positive association between global MTBQ score and self-reported disease burden score[15] fourth, a positive association between global MTBQ score and number of self-reported co-morbidities;[15] and fifth, a negative association between global MTBQ and self-rated health (single question item). We applied Spearman's rank correlation to test these hypotheses.

3c. Responsiveness

According to the ISOQOL guidelines, responsiveness to change should be assessed.[17] Due to the non-normal distribution of the global MTBQ score, standard methods to assess responsiveness to change such as calculating an effect size[22] were not possible. We therefore tested the responsiveness of the global MTBQ score by assessing whether changes over time in measures of quality of life (EQ-5D-5L)[14] and patient centred care (PACIC)[16] were inversely associated with changes in MTBQ as anticipated. We used a linear regression model of the standardised change in quality of life (EQ-5D-5L) score between baseline and nine-months on the

For peer review only - http://bmjopen.bmj.com/site/about/guidelines.xhtml

standardised change in MTBQ between baseline and nine-months. These standardised change scores were calculated at the participant level by dividing the individual difference in nine-month and baseline MTBQ (or EQ-5D-5L) score by the standard deviation of the overall MTBQ (or EQ-5D-5L) change score for all individuals. We then further adjusted this linear regression model in a subsequent analysis by age, gender, number of long-term conditions and individual participant deprivation level. All participants that died prior to the nine-month follow-up were given an EQ-5D-5L follow-up score of zero.

We then used the same model for MTBQ specified as above but included the standardised change in PACIC scores between baseline and 9-month follow-up, defined as previously, and subsequently further adjusted this model by the additional covariates as specified.

4. Interpretability of scores

The distribution of global MTBQ scores was examined and compared with the distribution of HCTD[6] scores.

We assessed interpretability of the questionnaire by grouping the global MTBQ scores greater than zero into tertiles. Four categories were generated: no burden (score 0), low burden (score<10), medium burden (10 to 22) and high burden (≥ 22)). Participant characteristics and key outcome variables, including EQ-5D-5L,[14] Bayliss disease burden score[15] and self-rated health, were compared across these four categories. To test for associations between treatment burden score category and participant characteristics we performed ordinal logistic regression of MTBQ group (four treatment burden categories) on each participant characteristic. We then further adjusted these ordinal logistic regression models by age, gender, number of co-morbidities, age left full time education and individual deprivation score.

5. Translation

Not applicable.

6. Demands on patient respondents and investigators

The effort required of patient respondents to complete the questionnaire was assessed during the cognitive interviews, and by reviewing the proportion of missing responses. We set out to reduce the demands on investigators by providing clear instructions on how to calculate a global MTBQ score, including handling of missing data, and how to report and interpret these scores.

Ethical approval and data sharing

The 3D study was approved by South-West (Frenchay) NHS Research Ethics Committee (14/SW/0011). Trial registration number: ISRCTN06180958. Data will be

For peer review only - http://bmjopen.bmj.com/site/about/guidelines.xhtml

available from the University of Bristol Research Data Storage Facility after the main results of the 3D trial have been published in 2018.

Results

Participant Characteristics

143 adults participated in the pilot study. From 1546 participants in the main 3D study who completed the main baseline questionnaire we were able to calculate a MTBQ score for 1524 (99%) individuals who completed at least half of the baseline MTBQ questions. At nine-month follow up, 1356 returned the questionnaire and a MTBQ score could be calculated for 1299 (96%). The participants were mostly elderly (mean age 71 years for the main study), fully retired from work, had left school aged 16 years or younger and 99% were white British (Table 1). Around two-thirds of participants from England lived in areas of low deprivation (low or middle lower quartiles), whereas almost two thirds of participants from Scotland lived in areas of high deprivation (middle upper or upper quartiles).

INSERT TABLE 1

1. Conceptual and measurement model

1a. Conceptual framework

The framework developed by Eton et al,[1] describes three major themes of treatment burden: the work required to look after one's health (e.g. self-monitoring, making lifestyle changes); tools and strategies patients use to reduce their treatment burden (e.g. organising medication); and factors that increase burden (e.g. poor continuity of care). We mapped the three existing treatment burden questionnaires against this framework, and discussed this with the PPI group who felt that all of the domains of treatment burden identified in the literature should be included in the PROM. We had initially considered excluding questions about costs since health care is mostly free under the National Health Service, but our PPI group argued that they still experienced additional costs from managing illness so this domain was retained in the first draft.

1b. Question properties

The proportion of missing data for each question was between 1% and 3% (see Table 2). Questions 3, 9 and 10 with a high proportion of 'does not apply' responses (Table 2) were excluded from the main analysis. Since these questions might apply to other populations we repeated Cronbach's alpha including these questions in the various combinations (Appendix B). These extra questions may be considered as optional depending on the study population. Responses were positively skewed and a floor effect was found for some questions. However, the MTBQ had fewer floor effects than the comparator HCTD (Appendix C).

For peer review only - http://bmjopen.bmj.com/site/about/guidelines.xhtml

The Global MTBQ scores were also skewed with 26% of pilot study participants and 22% of main study participants scoring zero (Appendix D). Again, the HCTD had greater floor effects, with 54% of participants having a global score of zero.

INSERT TABLE 2

1c. Dimensionality

Both Kaiser's "eigenvalue greater than one" rule and Cattell's scree plot criterion suggested a one factor solution and this explained 93% of the common variance. Loadings on this factor were uniformly greater than 0.4. The factor solution had high uniqueness for some items. This can sometimes indicate that the item is not strongly related to others,[18] but because of the important content of these variables (e.g. lifestyle changes, collecting medication), we chose to include them.

2. Reliability

Questions 1 and 2 have a high inter-item correlation of 0.69 and questions 6 and 7 have an inter-item correlation of 0.62 (Appendix E). Almost all of the other inter-item correlations were in the ideal range of 0.2 to 0.4. A decision was made to include questions 1 and 2, and 6 and 7 despite the high inter-item correlation coefficients because it was felt these questions were about different aspects of treatment burden. Cronbach's Alpha was 0.83 indicating a high level of internal reliability. Including the optional questions (questions 3, 9 and 10) in various combinations, Cronbach's Alpha ranged from 0.82 to 0.84, again demonstrating good internal consistency (see Appendix B).

3. Validity

3a. Face and Content validity

Participants from the PPI group commented that the wording was clear and easy to understand. One participant felt that accessing health care outside of usual GP opening hours caused significant treatment burden for him. In response to this, we added a question about difficulty getting health care in the evenings and weekends (question 10). The remaining participants commented that the important areas of treatment burden were covered by the questionnaire.

3b. Construct validity

As predicted, the global MTBQ score had a positive association with the comparator HCTD scale[6] (r_s 0.58, $p<0.0001$), the Bayliss disease burden scale[12] (r_s 0.43, $p<0.0001$) and the number of self-reported co-morbidities (r_s 0.32, $p<0.0001$); and a negative association with the quality of life scale[14] (r_s -0.36, $p<0.0001$) and self-rated health (r_s -0.36, $p<0.0001$) (Table 3). This provides good evidence for construct validity of the scale.

For peer review only - http://bmjopen.bmj.com/site/about/guidelines.xhtml

INSERT TABLE 3

3c. Responsiveness

Regression analysis found that for every 1 standard deviation (i.e. 0.17) increase in EQ-5D-5L score[14] between baseline and nine-month follow-up, MTBQ score at follow-up was reduced by 1.7 (regression coefficient -0.14 multiplied by a standard deviation change in MTBQ score of 11.9, (95% CI for regression coefficient -0.19 to -0.08), p value < 0.0001) (see Table 4). This association was also seen after further adjusting the model for the specified covariates (regression coefficient -0.14 (95% CI -0.20 to -0.08), p value <0.0001).

The equivalent model for PACIC score[16] showed that for every 1 standard deviation (i.e. 0.86) increase in PACIC score between baseline and nine-month follow-up, MTBQ at follow-up was reduced by 1.9 (regression coefficient -0.16 multiplied by a standard deviation change in MTBQ score of 11.9, (95% CI for regression coefficient -0.22 to -0.10), p value < 0.0001). A similar decrease was also seen after further adjusting the model for the specified covariates (regression coefficient -0.17, (95% CI -0.23 to -0.11), p value< 0.0001).

INSERT TABLE 4

4. Interpretability of scores

Comparing participants across the four treatment burden groups (no burden, low burden, medium burden and high burden) female participants; younger participants; those with a greater number of long-term conditions; participants with depression, dementia and severe mental health problems listed on their GP records; and participants with worse EQ-5D-5L scores,[14] high disease burden scores[12] and poor self-rated health were more likely to have a high treatment burden score, after adjusting for age, gender, number of co-morbidities, age left full time education and individual deprivation level (see Table 5). There was no convincing association between deprivation score and treatment burden score.

INSERT TABLE 5

5. Translation

Not applicable.

6. Demands on patient respondents and investigators

For peer review only - http://bmjopen.bmj.com/site/about/guidelines.xhtml

We have reduced the effort required from patient responders to complete the questionnaire by developing a short ten-item questionnaire with simple wording, fitting on one side of A4 paper in size 14 font. Participants who took part in the cognitive interviews found this relatively simple to complete and the proportion of missing data was between 1% and 3%. To reduce demands on investigators, we have provided clear instructions on calculating, reporting and interpreting global MTBQ scores.

Discussion

In this study, we have developed and validated a ten-item questionnaire, named the Multimorbidity Treatment Burden Questionnaire (MTBQ). The psychometric properties of the questionnaire meet the minimum standards for a PROM set out by ISOQOL,[17] demonstrating good content validity, internal reliability consistency, construct validity and responsiveness. Three additional questions, including one question about the cost of treatment, had a high proportion of 'does not apply' responses in this study population and were omitted from the main analysis. However, these questions may be relevant to other populations (e.g. countries where patients pay for prescriptions and health care) and the scale remained internally consistent and reliable when they were included, so they may be considered as optional.

We found that younger patients were more likely to report high treatment burden scores and, interestingly, the Tran TBQ found the same phenomenon.[5] There are several possible explanations for this. First, treatment burden may impact more on younger patients because they must juggle their appointments or complex medication regimens alongside having to work or look after dependants. Second, younger patients may have different expectations of how looking after one's health might impact on their lives and, hence, suffer from a greater perceived treatment burden. As expected, we found that patients with mental health conditions including depression and dementia were more likely to have high treatment burden scores. Previous studies have reported similar findings.[6 7] High treatment burden was also associated with having a greater number of long-term conditions. No individual physical condition was found to be associated with high treatment burden. This result differs from both the TBQ study, which found an association between treatment burden and diabetes, and the HCTD study, which found an association between treatment burden and stroke, congestive heart failure and falls.[5 6] As expected, participants with low quality of life (EQ-5D-5L)[14] score, high disease burden score[15] and poor self-rated health were more likely to have high treatment burden. We also found that female participants were more likely to report high treatment burden compared to males. This has not been reported elsewhere. There was no association between deprivation level and treatment burden score. One might expect that people from more deprived areas might have fewer support networks and resources and so would experience higher treatment burden. Alternatively, one could argue that participants from more deprived areas might be more accepting of how looking after their health impacts on their day-to-day life and so report lower treatment burden.

For peer review only - http://bmjopen.bmj.com/site/about/guidelines.xhtml

A key strength of this study is that the MTBQ has been validated in a large sample of participants for whom it is intended – elderly multimorbid patients with a mean age of 71 years and three or more long-term conditions. In comparison, the English version of the Tran Treatment Burden Questionnaire was validated in a younger computer-literate population with a mean age of 51 years.[4 5] The MTBQ had good face validity, was found to be user friendly and fits on a single page of A4 paper in size 14 font. All aspects of treatment burden identified in a comprehensive evidence based framework are included in the questionnaire. In comparison, the most comprehensive existing questionnaire, the PETS questionnaire,[8] includes 48 questions and is time consuming to complete, and several of the other existing questionnaires focus on only some aspects of treatment burden.[6 7] Preliminary assessment of responsiveness found that, as expected, a positive change in both quality of life (EQ-5D-5L)[14] score and patient centred care (PACIC)[16] score between baseline and nine-month follow-up was associated with a reduction in treatment burden (MTBQ) score. Of the other relevant PROMs, only the HCTD has been assessed for responsiveness[6] but the HCTD addresses fewer topics and has a narrower range of response options, possibly contributing to its greater problems with skewness and floor effects.

For peer review only - http://bmjopen.bmj.com/site/about/guidelines.xhtml

For peer review only

A limitation of this study is that the MTBQ was developed using a framework of treatment burden developed from qualitative study in the United States.[1] However, apart from the issue of paying for care, we felt that other domains of treatment burden were likely to be generalisable and we wanted to develop a measure that covered generic issues which would be relevant in a range of settings rather than specific to one health care system. Our measure was also informed by qualitative papers from different countries (including the UK) to ensure we included the important concepts.[10-12] In cognitive interviews, participants with multimorbidity felt that the questionnaire captured the range of factors that contribute to treatment burden.

A further limitation is that the participants of this study were recruited into a trial, which creates potential for selection bias and may limit generalisability. However, the trial participants had similar characteristics to those invited but declining participation in respect of age, gender, number and type of long-term conditions (data will be reported with the 3D trial results). Almost all the participants of this study were white British and further work is planned to validate the questionnaire in other populations. We found high floor effects with 22% of participants scoring a global MTBQ score of zero. All of the other treatment burden measures also show similarly high floor effects.[4-8] One explanation for this is a 'response shift', whereby patients adapt their everyday life so that looking after their health conditions becomes more acceptable to them over time and causes less perceived burden.[23] The implications of positively skewed treatment burden scores and high floor effects are: first, this can make it difficult to detect change (i.e. it is not possible to improve from a treatment burden score of zero); and second, mean treatment burden scores should be interpreted with caution. Preliminary analysis of responsiveness, however, has shown that changes in MTBQ score correlate as expected with changes in quality of life (EQ-5D-5L)[14] score and patient centred care (PACIC),[16] over time. We recommend that, due to the skewness of global MTBQ scores, researchers should report the median and interquartile range rather than the mean and standard deviation and report the proportion of patients with high, medium, low or no treatment burden (MTBQ scores ≥22, 10-22, <10 and 0 respectively).

The MTBQ scale is a concise measure of treatment burden for patients with multimorbidity that has demonstrated good content validity, construct validity, internal consistency reliability and responsiveness. It is a useful research tool for assessing the impact of interventions on treatment burden for patients with multimorbidity. We anticipate the scale being used alongside other measures, such as disease burden, and that findings from the two measures will be related. The MTBQ could also be used in clinical practice to highlight problem areas for patients with multimorbidity, such as difficulties the patient may have with their medication or with making recommended lifestyle changes. Further work is needed to validate the MTBQ for use in a clinical setting.

For peer review only - http://bmjopen.bmj.com/site/about/guidelines.xhtml

Acknowledgements

Appreciation is extended to members of the Patient and Public Involvement group, known as the Patient Involvement in Primary Care Research (PIP-CaRe) group, who took part in the cognitive interviews. The PIP-CaRe group was formed for the purpose of the 3D Study and consists of people with two or more long-term conditions. We also thank all other members of the 3D research team and Professor Boyd for permission to use the Healthcare Task Difficulty questionnaire.

Conflict of interest statement

This work was funded by the National Institute for Health Research Health Services and Delivery Research Programme (project number 12/130/15). The views and opinions expressed therein are those of the authors and do not necessarily reflect those of the HS&DR Programme, NIHR, NHS or the Department of Health.

Competing interests statement

None declared.

Author statement

PD, MSM, and CS were responsible for study concept and design. PD, MM, DG and KC were involved in data extraction and analysis. PD drafted the manuscript. All authors critically reviewed the manuscript and approved the final version. ***All authors*** also had full access to all of the data (including statistical reports and tables) in the study and can take responsibility for the integrity of the data and the accuracy of the data analysis. ***PD*** is the guarantor.

Dr Polly Duncan	PD led this project under supervision from Professor Chris Salisbury. She designed the study, undertook a literature review, developed the questionnaire, conducted and analysed the cognitive interviews, convened meetings with the patient and public involvement group, analysed the results and drafted the paper.
Dr Mairead Murphy	MM provided methodological expertise in assessing the psychometric properties of this new patient reported outcome measure, including the approach to analysis and interpretation of the results. She critically appraised the paper and has approved the final version.
Dr Mei-See Man	MSM provided methodological and practical expertise, and obtained ethical and governance approvals for this study. She

For peer review only - http://bmjopen.bmj.com/site/about/guidelines.xhtml

	critically appraised the paper and has approved the final version.
Dr Katherine Chaplin	KC acquired and cleaned the original data and produced the database used for analysis. She critically appraised the paper and has approved the final version.
Miss Daisy Gaunt	DG provided methodological expertise in analysing the responsiveness of the MTBQ and the interpretation of these results. She critically appraised the paper and has approved the final version.
Prof Chris Salisbury	CS was Chief Investigator on the 3D study which formed the basis for this paper, and supervised PD in developing this questionnaire. He contributed to study design, analysis and interpretation. He critically appraised the paper and has approved the final version.

For peer review only

For peer review only - http://bmjopen.bmj.com/site/about/guidelines.xhtml

Table 1: Participant Characteristics (main study N = 1546, pilot study N = 143)

			Pilot study n/N* (%)	Main study n/N* (%)
Mean age (SD)			74 (10)	71 (12)
Age (years)	≤ 50		3 (2)	79 (5)
	51-60		9 (6)	196 (13)
	61-70		27 (19)	420 (27)
	71-80		67 (47)	510 (33)
	81-90		33 (23)	315 (20)
	≥ 90		4 (3)	26 (2)
Gender	Male		65 (45)	763 (49)
Number of comorbidities	Three		109 (76)	1234 (80)
	Four		23 (16)	277 (18)
	Five		10 (7)	31 (2)
	Six		1 (<1)	4 (<1)
Comorbidities*	Cardiovascular disease		138 (97)	1445 (97)
	Stroke/TIA		35 (25)	527 (34)
	Diabetes		63 (44)	811 (52)
	Chronic kidney disease		83 (58)	464 (30)
	COPD or asthma		58 (41)	770 (50)
	Epilepsy		6 (4)	76 (5)
	Atrial fibrillation		46 (32)	529 (34)
	Severe mental health problems[a]		2 (1)	66 (4)
	Depression		26 (18)	560 (36)
	Dementia		6 (4)	60 (4)
	Learning disability		3 (2)	14 (1)
	Rheumatoid arthritis		9 (6)	103 (7)
	Heart failure		14 (10)	157 (10)
Ethnicity	White British		135/136 (99)	1502/1519 (99)
Age left full-time education (years)	≤ 14		22 (15)	154/1541 (10)
	15 or 16		74 (52)	907/1541 (59)
	17 or 18		25 (17)	222/1541 (14)
	≥ 19		22 (15)	258/1541 (17)
Employment status	Fully retired from work		113/139 (81)	1044/1501 (70)
Deprivation score quartile [b]	England	Lower quartile	99/143 (69)	445/1079 (41)
		Middle lower quartile	44/143 (31)	304/1079 (28)
		Middle upper quartile	0	196/1079 (18)
		Upper quartile	0	134/1079 (12)
	Scotland	Lower quartile		105/467 (22)
		Middle lower quartile		46/467 (10)
		Middle upper quartile		156/467 (33)
		Upper quartile		160/467 (34)
**Baseline scores of outcome measures**				
Mean HCTD score[c] (SD, N)			1.14 (1.7, 143)	
Mean self-reported disease burden score[d] (SD, N)				19 (12.4, 1458)
Mean number of self-reported conditions[e] (SD, N)				8 (3.2, 1543)
Mean quality of life score[f] (SD, N)				0.6 (0.3, 1542)
Mean self-rated health score[g] (SD, N)				2 (0.8, 1523)
Mean patient centred health score[h] (SD, N)				2.5 (1.0, 1232)

* For characteristics where there is no missing data n is shown, for characteristics with missing data n/N is shown. [a]Including schizophrenia and psychotic illness. [b]Individual Index of Multiple Deprivation (IMD) score, 2010, for England, and Scottish Index of Multiple Deprivation (SIMD) score, 2012, for Scotland, based on participants postcodes. The lower quartile is the least deprived and the upper quartile is the most deprived [c]Calculation of global HCTD score: sum of scores where each question was scored 0 (no difficulty), 1 (some difficulty), or 2 (a lot of difficulty). Minimum score 0, maximum score 16. Missing data was scored 0 (not difficult) as suggested by the HCTD authors[6] [d]Sum of the weighted scores (each scored 1-5) from the Bayliss scale.[12] Responses were excluded if participants ticked that they had a condition but did not score how much the condition limited their daily activity of if they gave a score without ticking that they had the condition. [e]Number of self-reported conditions from a list of 27 conditions itemised in the Bayliss scale. [f]EQ-5D-5L score.[11] [g]Single question. 'In general, would you say your health is poor (1), fair (2), good (3), very good (4) or excellent (5)?' [h]PACIC score[13]

For peer review only

For peer review only - http://bmjopen.bmj.com/site/about/guidelines.xhtml

Table 2: Responses to the Multimorbidity Treatment Burden Questionnaire (main study baseline data, N = 1546)

Please tell us how much difficulty you have with the following:	N	Not difficult n (n/N %)	A little difficult n (n/N %)	Quite difficult n (n/N %)	Very difficult n (n/N %)	Extremely difficult n (n/N %)	Does not apply n (n/N %)
1. Taking lots of medications	1518	1083 (71)	257 (17)	104 (7)	25 (2)	20 (1)	29 (2)
2. Remembering how and when to take medication	1519	1123 (74)	271 (18)	60 (4)	21 (1)	23 (2)	21 (1)
3. Paying for prescriptions, over the counter medication or equipment	1506	312 (21)	17 (1)	18 (1)	4 (<1)	8 (1)	1147 (76)
4. Collecting prescription medication	1514	951 (63)	221 (15)	63 (4)	22 (1)	28 (2)	229 (15)
5. Monitoring your medical conditions (eg. checking your blood pressure or blood sugar, monitoring your symptoms etc)	1513	748 (49)	191 (13)	111 (7)	35 (2)	37 (2)	391 (26)
6. Arranging appointments with health professionals	1507	765 (51)	321 (21)	210 (14)	81 (5)	66 (4)	64 (4)
7. Seeing lots of different health professionals	1506	642 (43)	309 (21)	192 (13)	85 (6)	68 (5)	210 (14)
8. Attending appointments with health professionals (eg. getting time off work, arranging transport etc)	1512	771 (51)	187 (12)	107 (7)	51 (3)	44 (3)	352 (23)
9. Getting health care in the evenings and at weekends	1496	311 (21)	156 (10)	184 (12)	106 (7)	121 (8)	618 (41)
10. Getting help from community services (eg. physiotherapy, district nurses etc)	1500	393 (26)	138 (9)	111 (7)	51 (3)	54 (4)	753 (50)
11. Obtaining clear and up-to-date information about your condition	1499	794 (53)	263 (18)	179 (12)	62 (4)	47 (3)	154 (10)
12. Making recommended lifestyle changes (eg. diet and exercise)	1505	534 (35)	327 (21)	203 (13)	112 (7)	75 (5)	254 (17)
13. Having to rely on help from family and friends	1509	675 (45)	213 (14)	140 (9)	59 (4)	70 (5)	352 (23)

Please note: Questions 3, 9 and 10 were excluded from the main analysis due to a high proportion of 'does not apply' responses. They are shown in italics. As they may be relevant to other populations, they can be considered as optional.

For peer review only

For peer review only - http://bmjopen.bmj.com/site/about/guidelines.xhtml

Table 3: Association between global MTBQ score and global HCTD score, self-reported disease burden score, quality of life score, number of self-reported conditions and self-rated health at baseline

Variable	N	Spearman's rank correlations (Rs)	P value
Global HCTD score[a]	141	0.58	< 0.0001
Self-reported disease burden score[b]	1443	0.42	< 0.0001
Number of self-reported conditions[c]	1523	0.31	< 0.0001
Quality of life score[d]	1520	-0.36	< 0.0001
Self-rated health[e]	1503	-0.36	< 0.0001

[a] Calculation of global HCTD score: sum of scores where each question was scored 0 (no difficulty), 1 (some difficulty), or 2 (a lot of difficulty). Minimum score 0, maximum score 16. Missing data was scored 0 (not difficult) as suggested by the HCTD authors[6] [b]Sum of the weighted scores (each scored 1-5) from the Bayliss scale.[12] Responses were excluded if participants ticked that they had a condition but did not score how much the condition limited their daily activity of if they gave a score without ticking that they had the condition. [c]Number of self-reported conditions from the Bayliss scale. [d]EQ-5D-5L score.[11] [e]Single question. 'In general, would you say your health is poor (1), fair (2), good (3), very good (4) or excellent (5)?'

For peer review only

For peer review only - http://bmjopen.bmj.com/site/about/guidelines.xhtml

Table 4: Association between global MTBQ score and (i) quality of life (EQ-5D-5L)[11] score; and (ii) Patient Assessment of Chronic Illness Care (PACIC)[13] score. Results from linear regression model of standardised change

Outcome	N[a]	Linear regression coefficient of MTBQ standardised change score (95% CI)	P value	N	Adjusted[b] linear regression coefficient of MTBQ standardised change score (95% CI)	P value
EQ-5D-5L standardised change score	1270	-0.14 (-0.19 to -0.08)	< 0.0001	1239	-0.14 (-0.20 to -0.08)	< 0.0001
PACIC standardised change score	930	-0.16 (-0.22 to -0.10)	< 0.0001	914	-0.17 (-0.23 to -0.11)	< 0.0001

Outcome	N[c]	Standard deviation change in score between baseline and nine-month follow-up
EQ-5D-5L	1344	0.17
PACIC	946	0.86
MTBQ	1285	11.9

[a] This analysis included participants who completed the outcome questionnaire (EQ-5D-5L or PACIC) and the MTBQ questionnaire at baseline and nine-month follow-up. [b] Linear regression model further adjusted for age, gender, number of co-morbidities, age left full time education and individual deprivation score. [c] This analysis included participants who completed the outcome questionnaire (EQ-5D-5L, PACIC or MTBQ) at baseline and nine-month follow-up

For peer review only - http://bmjopen.bmj.com/site/about/guidelines.xhtml

Table 5: Characteristics by categories of treatment burden (main study baseline data)

		N	None (0)	Low (<10)	Medium (10-22)	High (≥ 22)	Unadjusted OR*	Adjusted OR**	P value
**Participants**		1524	308	385	425	406			
**Age (mean)**		1524	74	73	71	66	**0.96 (0.95 to 0.97)**	**0.96 (0.95 to 0.97)**	**<0.0001**
**Gender [n, (%)]**	**Male**	651	168 (22)	208 (28)	193 (26)	182 (24)	**0.74 (0.62 to 0.88)**	**0.73 (0.60 to 0.87)**	**0.001**
**Number of long-term conditions [n,(%)]**	**Three**	1217	246 (20)	323 (27)	335 (28)	313 (26)			
	**Four or more**	307	62 (20)	62 (20)	90 (29)	93 (30)	1.21 (0.97 to 1.52)	**1.38 (1.09 to 1.74)**	**0.007**
**Long-term conditions [n, (%)]**	**Cardiovascular disease**	1423	294 (21)	367 (26)	389 (27)	373 (26)	**0.62 (0.44 to 0.91)**	0.79 (0.54 to 1.14)	0.208
	**Stroke/TIA**	517	127 (25)	140 (27)	135 (26)	115 (22)	**0.69 (0.57 to 0.83)**	0.82 (0.67 to 1.01)	0.059
	**Diabetes**	800	158 (20)	200 (25)	211 (26)	231 (29)	1.13 (0.94 to 1.35)	1.04 (0.87 to 1.26)	0.633
	**Chronic kidney disease**	454	101 (22)	121 (27)	115 (25)	117 (26)	0.86 (0.71 to 1.05)	1.10 (0.89 to 1.36)	0.356
	**COPD or asthma**	758	148 (20)	185 (24)	222 (29)	203 (27)	1.08 (0.90 to 1.29)	0.91 (0.75 to 1.10)	0.326
	**Epilepsy**	76	14 (18)	21 (28)	24 (32)	17 (22)	0.94 (0.63 to 1.41)	0.76 (0.50 to 1.17)	0.216
	**Atrial fibrillation**	524	119 (23)	155 (30)	142 (27)	108 (21)	0.68 (0.56 to 0.82)	0.91 (0.74 to 1.12)	0.369
	**Severe mental health problems[a]**	66	7 (11)	10 (15)	17 (26)	32 (48)	**2.61 (1.64 to 4.15)**	**1.75 (1.08 to 2.82)**	**0.022**
	**Depression**	553	85 (15)	105 (19)	169 (31)	194 (35)	**1.92 (1.59 to 2.32)**	**1.43 (1.16 to 1.77)**	**0.001**
	**Dementia**	58	14 (24)	10 (17)	12 (21)	22 (38)	1.27 (0.78 to 2.11)	**2.26 (1.34 to 3.81)**	**0.002**
	**Learning disability**	14	2 (14)	2 (14)	6 (43)	4 (29)	1.47 (0.59 to 3.69)	1.07 (0.36 to 3.21)	0.907
	**Rheumatoid arthritis**	102	15 (15)	18 (18)	40 (39)	29 (28)	1.41 (0.99 to 2.01)	1.28 (0.88 to 1.82)	0.202
	**Heart failure**	154	36 (23)	41 (27)	38 (25)	39 (25)	0.85 (0.63 to 1.14)	1.06 (0.77 to 1.44)	0.340
**Age left full-time education [n, (%)]**	**≤16 years**	681	164 (24)	172 (25)	177 (26)	168 (25)	1.00 (0.99 to 1.01)	1.01 (0.99 to 1.02)	0.450
**Deprivation score (mean)***	**England**	1078	15	15	15	16	1.01 (1.00 to 1.01)	1.00 (0.99 to 1.01)	0.904
	**Scotland**	467	26	26	24	24	1.00 (0.99 to 1.01)	**0.99 (0.99 to 1.00)**	**0.032**
**EQ-5D-5L[11] (mean)**		1520	0.67	0.63	0.56	0.42	**0.11 (0.08 to 0.16)**	**0.09 (0.06 to 0.12)**	**<0.0001**
**Disease-burden score[12] (mean)**		1443	12.8	15.7	19.0	26.1	**1.06 (1.06 to 1.08)**	**1.07 (1.07 to 1.09)**	**<0.0001**
**Self-rated health [n, (%)]**	**Poor**	315	36 (11)	42 (13)	75 (24)	162 (51)			
	**Fair**	674	112 (17)	168 (25)	216 (32)	178 (26)	**0.39 (0.30 to 0.50)**	**0.41 (0.31 to 0.53)**	**<0.0001**
	**Good**	422	111 (26)	138 (33)	116 (27)	57 (14)	**0.20 (0.15 to 0.26)**	**0.19 (0.14 to 0.26)**	**<0.0001**
	**Very good**	87	40 (46)	28 (32)	16 (18)	3 (3)	**0.08 (0.05 to 0.13)**	**0.08 (0.05 to 0.12)**	**<0.0001**

For peer review only

For peer review only - http://bmjopen.bmj.com/site/about/guidelines.xhtml

	**Excellent**	5	3 (60)	2 (40)	0	0	**0.04 (0.01 to 0.23)**	**0.03 (0.00 to 0.16)**	**<0.0001**

*ordinal logistic regression comparing no burden (0), low burden (<10), medium burden (10-22) and high burden (≥22) ** ordinal logistic regression comparing no burden (0), low burden (<10), medium burden (10-22) and high burden (≥22), adjusted for age, gender, number of co-morbidities, age left full time education and individual deprivation score *** Individual Index of Multiple Deprivation (IMD) score, 2010, for England, and Scottish Index of Multiple Deprivation (SIMD) score, 2010, for Scotland, for both a higher score correlates with greater deprivation. [a]Including schizophrenia and psychotic illesss

For peer review only

References

1. Eton DT, Ramalho de Oliveira D, Egginton JS, et al. Building a measurement framework of burden of treatment in complex patients with chronic conditions: a qualitative study. *Patient Relat Outcome Meas* 2012;3:39-49. doi: 10.2147/prom.s34681 [published Online First: 2012/11/28]
2. Salisbury C, Johnson L, Purdy S, et al. Epidemiology and impact of multimorbidity in primary care: a retrospective cohort study. *Br J Gen Pract* 2011;61(582):e12-21.
3. May CR, Eton DT, Boehmer K, et al. Rethinking the patient: using Burden of Treatment Theory to understand the changing dynamics of illness. *BMC Health Serv Res* 2014;14:281. doi: 10.1186/1472-6963-14-281 [published Online First: 2014/06/28]
4. Tran VT, Harrington M, Montori VM, et al. Adaptation and validation of the Treatment Burden Questionnaire (TBQ) in English using an internet platform. *BMC Med* 2014;12:109. doi: 10.1186/1741-7015-12-109 [published Online First: 2014/07/06]
5. Tran VT, Montori VM, Eton DT, et al. Development and description of measurement properties of an instrument to assess treatment burden among patients with multiple chronic conditions. *BMC Medicine* 2012;10:68. doi: http://dx.doi.org/10.1186/1741-7015-10-68
6. Boyd CM, Wolff JL, Giovannetti E, et al. Healthcare task difficulty among older adults with multimorbidity. *Med Care* 2014;52 Suppl 3:S118-25. doi: 10.1097/MLR.0b013e3182a977da [published Online First: 2014/02/25]
7. Gibbons CJ, Kenning C, Coventry PA, et al. Development of a multimorbidity illness perceptions scale (MULTIPleS). *PLoS ONE [Electronic Resource]* 2013;8(12):e81852. doi: http://dx.doi.org/10.1371/journal.pone.0081852
8. Eton DT, Yost KJ, Lai JS, et al. Development and validation of the Patient Experience with Treatment and Self-management (PETS): a patient-reported measure of treatment burden. *Qual Life Res* 2016 doi: 10.1007/s11136-016-1397-0 [published Online First: 2016/08/26]
9. Man MS, Chaplin K, Mann C, et al. Improving the management of multimorbidity in general practice: protocol of a cluster randomised controlled trial (The 3D Study). *Bmj Open* 2016;6(4):e011261. doi: 10.1136/bmjopen-2016-011261
10. Bayliss EA, Steiner JF, Fernald DH, et al. Descriptions of barriers to self-care by persons with comorbid chronic diseases. *Ann Fam Med* 2003;1(1):15-21. [published Online First: 2004/03/27]
11. Morris RL, Sanders C, Kennedy AP, et al. Shifting priorities in multimorbidity: a longitudinal qualitative study of patient's prioritization of multiple conditions. Chronic Illn. United States2011:147-61.

For peer review only - http://bmjopen.bmj.com/site/about/guidelines.xhtml

12. Bower P, Harkness E, Macdonald W, et al. Illness representations in patients with multimorbid long-term conditions: qualitative study. *Psychol Health* 2012;27(10):1211-26. doi: 10.1080/08870446.2012.662973 [published Online First: 2012/03/07]
13. Willis GB, Casper RA, Lessler JT. Cognitive Interviewing: A " How To" Guide. Short course presented at the Meeting of American Statistical Association, 1999.
14. EuroQol--a new facility for the measurement of health-related quality of life. *Health Policy* 1990;16(3):199-208. [published Online First: 1990/11/05]
15. Bayliss EA, Ellis JL, Steiner JF. Seniors' self-reported multimorbidity captured biopsychosocial factors not incorporated into two other data-based morbidity measures. *J Clin Epidemiol* 2009;62(5):550-7.e1. doi: 10.1016/j.jclinepi.2008.05.002 [published Online First: 2008/09/02]
16. Glasgow RE, Wagner EH, Schaefer J, et al. Development and validation of the Patient Assessment of Chronic Illness Care (PACIC). *Med Care* 2005;43(5):436-44.
17. Reeve BB, Wyrwich KW, Wu AW, et al. ISOQOL recommends minimum standards for patient-reported outcome measures used in patient-centered outcomes and comparative effectiveness research. *Qual Life Res* 2013;22(8):1889-905. doi: 10.1007/s11136-012-0344-y [published Online First: 2013/01/05]
18. Tabachnick BG, Fidell LS, Dawsonera. Using multivariate statistics. 6th ed2014.
19. Factor Analysis http://www.stata.com/manuals13/mvfactor.pdf2015 [accessed 22.08.2017.
20. Kaiser HF. The application of electronic computers to factor analysis. Educational and Psychological Measurement, 1960:141-51.
21. Piedmont RL. Inter-item Correlations. In: Michalos AC, ed. Encyclopedia of Quality of Life and Well-Being Research. Dordrecht: Springer Netherlands 2014:3303-04.
22. Streiner DL, Norman GR. Health Measurement Scales a practical guide to their development and use. Fourth edition ed: Oxford University Press 2008.
23. Sprangers MAG, Schwartz CE. Integrating response shift into health-related quality of life research: a theoretical model. *Social Science & Medicine* 1999;48(11):1507-15. doi: http://dx.doi.org/10.1016/S0277-9536(99)00045-3

For peer review only

For peer review only - http://bmjopen.bmj.com/site/about/guidelines.xhtml

Appendix A: Characteristics of the participants who took part in the cognitive interviews (n=8)

Characteristic	Value
Mean age years (SD, min, max)	55.5 (14.1, 30, 78)
Male	2 (25%)
White British ethnicity	8 (100%)
Mean number of self-reported long-term conditions (SD, min, max)	2.1 (1.5, 1, 5)

For peer review only

Appendix B: Cronbach's alpha including the optional questions (questions 3, 9 and 10) in the various combinations

	Optional questions						
	**3**	**9**	**10**	**3, 9, 10**	**3, 9**	**3, 10**	**9, 10**
**Cronbach's alpha**	0.82	0.83	0.83	0.84	0.83	0.83	0.84

Optional questions: Please tell us how much difficulty you have with the following:

Question 3. Paying for prescriptions, over the counter medication or equipment
Question 9. Getting health care in the evenings and at weekends
Question 10. Getting help from community services (e.g. physiotherapy, district nurses etc)

For peer review only

Appendix C: A comparison of the floor effects and missing data of the MTBQ and the HCTD (pilot study data)

MTBQ Question	Floor effect[a] %	Missing data %	HCTD question with a similar latent construct	Floor effect [b] %	Missing data (%)
**1.** Taking lots of medications	78	1	3. Difficulty taking medications	95	1
**2.** Remembering how and when to take medication	80	1	2. Difficulty planning medication schedule	94	3
3. *Paying for prescriptions, over the counter medication or equipment*	94	4	5. Difficulty paying prescription charges	78	19
**4.** Collecting prescription medication	83	2	1. Difficulty obtaining medications	87	1
**5.** Monitoring your medical conditions (eg. checking your blood pressure or blood sugar, monitoring your symptoms etc)	83	2	No question to compare with		
**6.** Arranging appointments with health professionals	59	3	6. Difficulty scheduling medical appointment	69	4
**7.** Seeing lots of different health professionals	62	2	No question to compare with		
**8.** Attending appointments with health professionals (eg. getting time off work, arranging transport etc)	74	1	7. Difficulty arranging transportation	76	6
9. *Getting health care in the evenings and at weekends*	70	3	No question to compare with		
10. *Getting help from community services (eg. physiotherapy, district nurses etc)*	83	2	No question to compare with		
**11.** Obtaining clear and up-to-date information about your condition	70	2	8. Difficulty getting information	74	4
**12.** Making recommended lifestyle changes (eg. diet and exercise)	57	3	No question to compare with		
**13.** Having to rely on help from family and friends	69	1	No question to compare with		

[a] proportion (%) of 'does not apply' or 'not difficult' responses
[b] proportion (%) 'not difficult' responses
Please note: Questions 3, 9 and 10 were excluded from the main analysis due to a high proportion of 'does not apply' responses. They are shown in italics. As they may be relevant to other populations, they can be considered as optional

For peer review only

Appendix D: Histogram of global MTBQ scores and global HCTD scores (pilot study and main study)

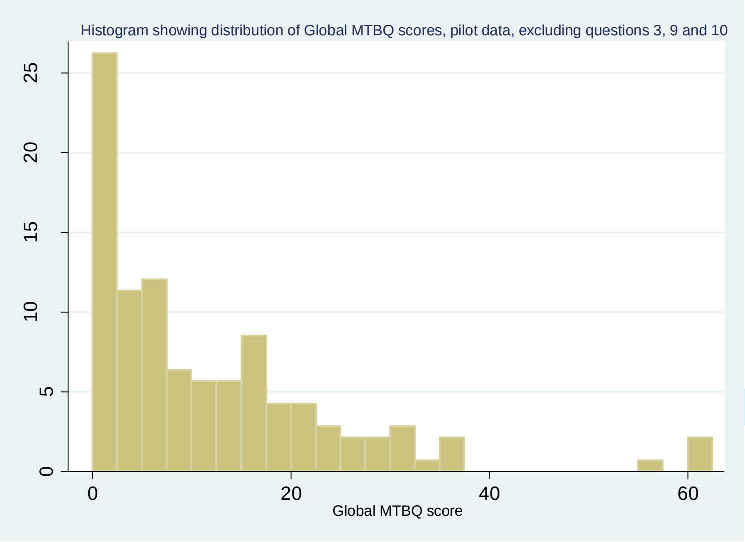

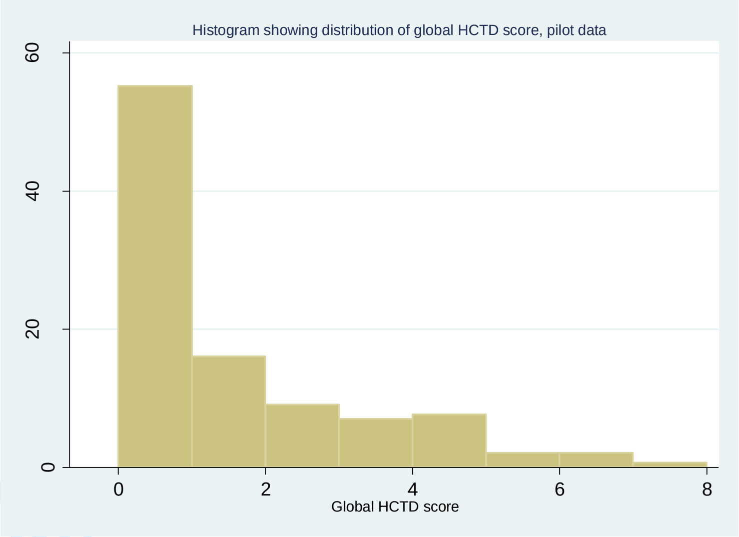

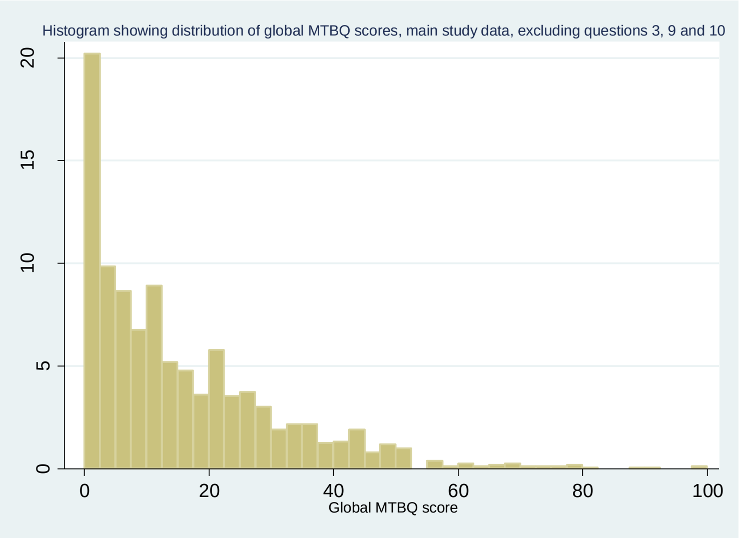

For peer review only - http://bmjopen.bmj.com/site/about/guidelines.xhtml

Appendix E: Inter-item correlation coefficient and Cronbach's Alpha (main study data, excluding questions 3, 9 and 10)

Cronbach's alpha = 0.83

Question:	1	2	4	5	6	7	8	11	12	13
**1**	1.00									
**2**	0.69	1.00								
**4**	0.30	0.26	1.00							
**5**	0.35	0.33	0.31	1.00						
**6**	0.26	0.23	0.28	0.31	1.00					
**7**	0.34	0.29	0.29	0.38	0.62	1.00				
**8**	0.32	0.32	0.40	0.33	0.37	0.44	1.00			
**11**	0.24	0.19	0.27	0.27	0.45	0.46	0.33	1.00		
**12**	0.28	0.27	0.23	0.32	0.29	0.34	0.31	0.35	1.00	
**13**	0.32	0.25	0.30	0.26	0.28	0.34	0.40	0.29	0.33	1.00

Questions: Please tell us how much difficulty you have with the following:

1. Taking lots of medications
2. Remembering how and when to take medication
4. Collecting prescription medication
5. Monitoring your medical conditions (eg. checking your blood pressure or blood sugar, monitoring your symptoms etc)
6. Arranging appointments with health professionals
7. Seeing lots of different health professionals
8. Attending appointments with health professionals (eg. getting time off work, arranging transport etc)
11. Obtaining clear and up-to-date information about your condition
12. Making recommended lifestyle changes (eg. diet and exercise)
13. Having to rely on help from family and friends

Please note: Questions 3, 9 and 10 were excluded from the main analysis due to a high proportion of 'does not apply' responses.

For peer review only

Qual Life Res
DOI 10.1007/s11136-012-0344-y

ISOQOL recommends minimum standards for patient-reported outcome measures used in patient-centered outcomes and comparative effectiveness research

Bryce B. Reeve · Kathleen W. Wyrwich · Albert W. Wu · Galina Velikova · Caroline B. Terwee · Claire F. Snyder · Carolyn Schwartz · Dennis A. Revicki · Carol M. Moinpour · Lori D. McLeod · Jessica C. Lyons · William R. Lenderking · Pamela S. Hinds · Ron D. Hays · Joanne Greenhalgh · Richard Gershon · David Feeny · Peter M. Fayers · David Cella · Michael Brundage · Sara Ahmed · Neil K. Aaronson · Zeeshan Butt

Accepted: 17 December 2012
© Springer Science+Business Media Dordrecht 2013

Abstract

Purpose An essential aspect of patient-centered outcomes research (PCOR) and comparative effectiveness research (CER) is the integration of patient perspectives and experiences with clinical data to evaluate interventions. Thus, PCOR and CER require capturing patient-reported outcome (PRO) data appropriately to inform research, health-care delivery, and policy. This initiative's goal was to identify minimum standards for the design and selection of a PRO measure for use in PCOR and CER.

Methods We performed a literature review to find existing guidelines for the selection of PRO measures. We also conducted an online survey of the International Society for Quality of Life Research (ISOQOL) membership to solicit input on PRO standards. A standard was designated as "recommended" when >50 % respondents endorsed it as "required as a minimum standard."

Results The literature review identified 387 articles. Survey response rate was 120 of 506 ISOQOL members. The respondents had an average of 15 years experience in PRO research, and 89 % felt competent or very competent providing feedback. Final recommendations for PRO measure standards included: documentation of the conceptual and measurement model; evidence for reliability, validity (content validity, construct validity, responsiveness); interpretability of scores; quality translation, and acceptable patient and investigator burden.

Conclusion The development of these minimum measurement standards is intended to promote the appropriate use of PRO measures to inform PCOR and CER, which in turn can improve the effectiveness and efficiency of healthcare delivery. A next step is to expand these

This study was conducted on behalf of the International Society for Quality of Life Research (ISOQOL).

Electronic supplementary material The online version of this article (doi:10.1007/s11136-012-0344-y) contains supplementary material, which is available to authorized users.

B. B. Reeve (✉)
Department of Health Policy and Management, Gillings School of Global Public Health, University of North Carolina at Chapel Hill, 1101-D McGavran-Greenberg Building, 135 Dauer Drive, CB 7411, Chapel Hill, NC 27599-7411, USA
e-mail: bbreeve@email.UNC.edu

B. B. Reeve · J. C. Lyons
Lineberger Comprehensive Cancer Center, University of North Carolina at Chapel Hill, Chapel Hill, NC, USA

K. W. Wyrwich · D. A. Revicki · W. R. Lenderking
United BioSource Corporation, Bethesda, MD, USA

A. W. Wu · C. F. Snyder
Johns Hopkins School of Medicine, Baltimore, MD, USA

G. Velikova · J. Greenhalgh
University of Leeds, Leeds, UK

C. B. Terwee
VU University Medical Center, Amsterdam, The Netherlands

C. Schwartz
DeltaQuest Foundation, Inc., Concord, MA, USA

C. M. Moinpour
Fred Hutchinson Cancer Research Center, Seattle, WA, USA

L. D. McLeod
Research Triangle Institute Health Solutions, Durham, NC, USA

Published online: 04 January 2013

For peer review only - http://bmjopen.bmj.com/site/about/guidelines.xhtml

minimum standards to identify best practices for selecting decision-relevant PRO measures.

Keywords Patient-reported outcomes · Comparative effectiveness · Patient-centered outcomes research · Psychometrics · Questionnaire

Introduction

An essential aspect of patient-centered outcomes research (PCOR) and comparative effectiveness research (CER) is the integration of patients' perspectives about their health with clinical and biological data to evaluate the safety and effectiveness of interventions. Such integration recognizes that health-related quality of life (HRQOL) and how it is affected by disease and treatment complements traditional clinical endpoints such as survival or tumor response in cancer. For HRQOL endpoints, it is widely accepted that the patient's report is the best source of information about what he or she is experiencing. The challenge for PCOR and CER is how to best capture patient-reported data in a way that can inform decision making in healthcare delivery, research, and policy settings.

Observational and experimental studies have increasingly included patient-reported outcome (PRO) measures, defined by the Food and Drug Administration (FDA) as "any report of the status of a patient's health condition that comes directly from the patient, without interpretation of the patient's response by a clinician or anyone else [1]." Patients can report accurately on a number of domains that are important for evaluating an intervention or disease burden, including symptom experiences (e.g., pain, fatigue, nausea), functional status (e.g., sexual, bowel, or urinary functioning), well-being (e.g., physical, mental, social), quality of life, and satisfaction with care or with a treatment [1–4]. Arguably, patients are the gold standard source of information for assessing such domains. To draw valid research conclusions regarding patient-centered outcomes, PROs must be measured in a standardized way using scales that demonstrate sufficiently robust measurement properties [4–9].

The goal of this study was to identify minimum standards for the selection of PRO measures for use in PCOR and CER. We defined minimum standards such that if a PRO measure did not meet these criteria, it would be judged not suitable for a PCOR study. A central aim in developing this set of standards was to clearly define the critical attributes for judging a PRO measure for a PCOR study. We identified these standards using two complementary approaches. The first was an extensive review of the literature including both published and unpublished guidance documents. The second was to seek input, via a formal survey, from an international group of experts in PRO measurement and PCOR who are members of the International Society for Quality of Life Research (ISOQOL) [10]. Although not the primary objective of this study, our approach allowed us to also identify criteria that were not deemed as a necessary minimum standard, but would rather be considered "best practice" standards for PRO measures.

Identification of minimal standards is a first step toward enabling PCOR and CER to achieve their goals of enhancing healthcare delivery and ultimately improving patients' health and well-being. Access to scientifically sound and decision-relevant PRO measures will allow investigators to collect empirical evidence on the differential benefits of interventions from the patients' perspective [6, 9, 11, 12]. This information can then be disseminated to patients, providers, and policy makers to provide a richer perspective on the impact of interventions on patients' lives using endpoints that are meaningful to them [13].

P. S. Hinds
Children's National Medical Center, Washington, DC, USA

P. S. Hinds
The George Washington University School of Medicine, Washington, DC, USA

R. D. Hays
David Geffen School of Medicine at UCLA, Los Angeles, CA, USA

R. Gershon · D. Cella · Z. Butt
Northwestern University Feinberg School of Medicine, Chicago, IL, USA

D. Feeny
University of Alberta, Alberta, Canada

P. M. Fayers
University of Aberdeen, Aberdeen, UK

P. M. Fayers
Norwegian University of Science and Technology (NTNU), Trondheim, Norway

M. Brundage
Queen's University, Kingston, ON, Canada

S. Ahmed
McGill University, Montreal, QC, Canada

N. K. Aaronson
The Netherlands Cancer Institute, Amsterdam, The Netherlands

N. K. Aaronson
University of Amsterdam, Amsterdam, The Netherlands

For peer review only - http://bmjopen.bmj.com/site/about/guidelines.xhtml

Methods

This paper is based on a study funded by the U.S. Patient-Centered Outcomes Research Institute (PCORI) [14]. The paper does not represent PCORI's Methodology Committee standards, issued separately by PCORI, though some of those standards were informed by this work [15]. An ISOQOL scientific advisory task force (SATF), consisting of the authors on this article, was set up to guide the drafting and final selection of recommended standards. We conducted a literature review that helped the SATF draft the recommendations that were subsequently reviewed by ISOQOL members in the formal survey. The literature review and the responses and feedback from ISOQOL members informed the final recommendations provided in this article.

Literature review

We conducted a systematic review of the published and unpublished literature to identify existing guidance documents related to PRO measures. The review identified current practices in selecting PRO measures in PCOR and CER, relevant scale attributes (e.g., reliability, validity, response burden, interpretability), and use of qualitative and quantitative methods to assess these properties. We focused on consensus statements, guidelines, and evidence-based papers, with an emphasis on articles or documents that described broadly generalizable principles. However, some papers that were population- or instrument-specific were included because of the rigor of the psychometric methods.

For the literature review, we adapted a published MEDLINE search strategy to identify measurement properties of PRO measures [16]. The published strategy was used as a foundation and adapted by using terms from MEDLINE thesaurus, Medical Subject Headings (MeSH), and the American Psychological Association's (APA) online Thesaurus of Psychological terms. We conducted parallel searches in several relevant electronic databases, including MEDLINE, PsycINFO, and Combined Index to Nursing and Allied Health Literature (CINAHL) (see database search terms in Appendix 1, ESM). There was no a priori restriction by publication date or age of sample. We also obtained relevant articles through a request to the ISOQOL membership email distribution list.

The titles and abstracts of identified articles and guidelines were reviewed by one of the authors (ZB). The full text of relevant articles was obtained and reviewed. The references cited in the selected articles were reviewed to identify additional relevant articles. ZB abstracted the necessary information for the study; two other authors (DC and RG) independently reviewed several of the articles to ensure coding consistency.

Based on PRO measurement standards gleaned from the literature review, the ISOQOL SATF drafted recommendations that were reviewed by ISOQOL members in a survey described below. Through an iterative series of SATF e-mails and conference calls, the potential standards identified by the systematic literature review were discussed and debated. Redundancies between potential standards were minimized, and similar items consolidated. Where there were differences in opinion among the members, different options were retained in the survey in order that the membership at large could rate and comment on each potential standard. The resultant survey consisted of 23 potential minimum standards to be rated by the ISOQOL membership.

Survey of ISOQOL membership

ISOQOL is dedicated to advancing the scientific study of HRQOL and other patient-centered outcomes to identify effective interventions, enhance the quality of healthcare and promote the health of populations [10]. Since 1993, ISOQOL has been an international collaborative network including researchers, clinicians, patient advocates, government scientists, industry representatives, and policy makers. Many ISOQOL members are PRO methodologists who focus on using state-of-the-art methods, both qualitative and quantitative, to improve the measurement and application of patient-reported data in research, healthcare delivery, and population surveillance. Many of the PRO measures widely used in research as well as the guidelines for developing and evaluating a PRO measure were developed by ISOQOL members. At the time of the survey, there were 506 ISOQOL members on the email distribution list.

In the web-based survey, we sought ISOQOL members' views on draft minimum standards, paying particular attention to areas where there did not appear to be consensus in the literature. For example, we asked ISOQOL members to rank the relative importance of various approaches for assessing reliability, including test–retest and internal consistency for multi-item PRO measures. In addition, we sought agreement on recommendations for six key attributes of PRO measures: (1) conceptual and measurement model, (2) reliability, (3) validity, (4) interpretability of scores, (5) translation, and (6) patient and investigator burden.

In the survey, it was deemed critical that respondents had a clear definition of a minimum standard. The second screen of the survey provided this guidance: "Please remember as you answer the questions in this survey that we are developing the minimum standards for the selection and design of a PRO measure for use in patient-centered outcomes research (PCOR). That is, we are saying a PRO measure that does not meet the *minimum standard* should not be considered appropriate for the research study." This statement was not intended to suggest that a PRO measure would not continue to be validated and strengthened as part of a maturation model of development. The survey directly mentioned PCOR, but the SATF believes these recommendations

For peer review only - http://bmjopen.bmj.com/site/about/guidelines.xhtml

are consistent for CER. For brevity, we use just "PCOR" in describing the results.

For each recommendation created by the SATF's synthesis of the literature review, the participant could select one of the following response options: required as a minimum standard, desirable but not required as a minimum standard, not required at all (not needed for a PRO measure), not sure, or no opinion. In analyzing the results, we used the general rule that if 50 % or more agreed that the recommendation was required as a minimum standard, then the recommendation was accepted. If less than 50 % of respondents were in agreement, then the recommendation was reviewed by the ISOQOL SATF to determine whether the recommendation may have been unclear or whether it would better be considered as a "best practice" (or "ideal standard") for PRO measures rather than a "minimum standard." Respondents were also encouraged to comment using a free text box that was provided after each recommendation. This text was extracted from the survey and helped inform the ISOQOL SATF's decisions and final recommendations.

The survey and a description of the survey methodology were submitted to the Institutional Review Board (IRB) at the University of North Carolina at Chapel Hill (UNC) for review and were determined to be exempt from IRB approval by the UNC Office of Human Research and Ethics. The online survey was designed and administered using the Qualtrics Software System under the UNC site license [17].

The survey link was sent out through the ISOQOL member email distribution list ($n = 506$) on 20 February , 2012. Survey instructions asked members to complete the survey within 9 days to meet deadlines for the PCORI contract. However, the response interval was extended to 20 March , 2012 (29 days), to accommodate more ISOQOL respondents. Information about the purpose of the voluntary survey, goals of the project, and funding source was included. All responses were anonymous, and no personal identifying information was collected. Two reminders were sent during the period the survey was available.

We did not expect responses from all ISOQOL members, because: (1) the survey was specifically aimed at those ISOQOL members who considered themselves to have the requisite expertise in the area of PRO measurement, and (2) we sought expert input in a short amount of time. Although we did not limit eligibility to those members who had such expertise, we did ask respondents to self-report their expertise level as part of the survey.

Results

Guidance identified through the literature review

A number of well-known guidance documents were identified, including guidance from the FDA [1, 18–20]; the 2002 Medical Outcomes Trust guidelines on attributes of a good HRQOL measure [2]; the extensive, international expert-driven recommendations from COSMIN (COnsensus-based Standards for the selection of health Measurement INstruments) [3, 4, 21–25]; the European Organization for Research and Treatment of Cancer (EORTC) guidelines for developing questionnaires [26]; the Functional Assessment of Chronic Illness Therapy (FACIT) approach [27]; the International Society for Pharmacoeconomics and Outcomes Research (ISPOR) task force recommendation documents [28–31]; the American Psychological Association (APA) Standards for Educational and Psychological Testing [32]; and several others [33–38]. We also had access to the recent standards documents just completed by the National Institutes of Health's Patient-Reported Outcomes Measurement Information System® (PROMIS®) network, which we considered useful for informing the minimal standards for PRO measures. In addition, ISOQOL recently completed two guidance documents relevant for this landscape review on the use of PRO measures in comparative effectiveness research and on integrating PRO measures in healthcare delivery settings [5, 39].

ISOQOL members identified a total of 301 additional references relevant for our task. Our formal search of the MEDLINE database yielded 821 references, which were individually reviewed, resulting in 60 additional relevant articles. Review of the 172 potentially relevant PsycINFO results provided 22 additional relevant articles, and an additional four unique references were uncovered after review of 126 abstracts identified through CINAHL.

Table 1 describes 28 key guidance documents identified from the literature review that helped to inform the ISOQOL SATF's draft minimum guidelines to be evaluated in the ISOQOL survey. The documents selected for further review and discussion by our ISOQOL SATF represented exemplar description of guidelines and standards for the selection of PRO in PCOR. As part of our literature review, we identified many more relevant references; however, our focus was on existing guidance documents that had broad relevance. Multiple publications describing the same set of guidelines were not cited separately.

Characteristics of participants responding to the ISOQOL survey

Table 2 summarizes the characteristics of the 120 ISOQOL members (23.7 %) who responded to the survey. Approximately 64 % of the sample had a PhD (or similar doctoral degree) and 18 % had a MD. The sample included 68 % academic researchers, 21 % clinicians, 8 % industry representatives, 23 % industry consultants, and 6 % federal government employees. There was diverse geographic distribution with 48 % of respondents from North America

For peer review only - http://bmjopen.bmj.com/site/about/guidelines.xhtml

Table 1 Identified guidelines for patient-reported outcomes measures

Author, year	Guideline	Research design	Description
Acquadro et al. [48]	The Literature review of methods to translate health-related quality of life questionnaires for use in multinational clinical trials	Formal literature review	Call for more empirical research on translation methodology; reviews several existing guidelines; advocates multistep process for translations
Cella [27]	Manual for the Functional Assessment of Chronic Illness Therapy (FACIT)	Description of method	Provides summary of FACIT scale development and translation methodologies; presents basic psychometric info for existing measures
Coons et al. [28]	Recommendations on evidence needed to support measurement equivalence between electronic and paper-based patient-reported outcome measures	Expert opinion and literature review	Provides a general framework for decisions regarding evidence needed to support migration of paper PRO measures to electronic delivery
COSMIN group, 2010 [24]	COSMIN study: COnsensus-based Standards for the selection of health Measurement INstruments	Guidelines established via systematic literature review and iterative Delphi process	Consensus was reached on design requirements and preferred statistical methods for the assessment of internal consistency, reliability, measurement error, content validity, construct validity, criterion validity, responsiveness, and interpretability
Crosby et al. [49]	Defining clinically meaningful change in health-related quality of life	Literature review	Reviews current approaches to defining clinically meaningful change in health-related quality of life and provides guidelines for their use
Dewolf et al. [36]	Translation procedure	Expert opinion	Provides guidance on the methodology for translating EORTC Quality of Life Questionnaires (QLQ)
Erickson et al. [19]	A concept taxonomy and an instrument hierarchy: tools for establishing and evaluating the conceptual framework of a patient-reported outcome (PRO) instrument as applied to product labeling claims	Expert opinion	Proposes a PRO concept taxonomy and instrument hierarchy that may be useful for demonstration of PRO measure claim for drug development, although they have not been tested for such purpose
Frost et al. [50]	What is sufficient evidence for the reliability and validity of patient-reported outcome measures?	Literature review	Article provides specific guidance on necessary psychometric properties of a PRO measure, with special reference to the FDA guidance, using the literature as a guide for specific statistical thresholds
Hays et al. [51]	The concept of clinically meaningful change in health-related quality of life research: How meaningful is it?	Expert opinion	Argues against a single threshold to define the minimally clinical important difference
Johnson et al. [26]	Guidelines for developing questionnaire modules	Expert opinion	Provides detailed description of PRO measure module development per the EORTC methodology related to generation of issues, construction of item list, pre- and field-testing
Kemmler et al. [52]	A new approach to combining clinical relevance and statistical significance for evaluation of quality of life changes in the individual patient	Longitudinal data from a chemotherapy trial	Data from this trial were used to evaluate change for individual participants (vs. groups). Stressed the importance of evaluation on the basis of statistical and clinical significance
Kottner et al. [53]	Guidelines for reporting reliability and agreement studies (GRRAS) were proposed	Literature review and expert consensus	Proposes a set of guidelines for reporting inter-rater agreement, inter-rater reliability in healthcare and medicine
Magasi et al. [33]	Content validity of patient-reported outcome measures: Perspectives from a PROMIS meeting	Expert presentation and discussion	The paper describes findings from a PROMIS meeting focused on content validity. Several recommendations were outlined as a result, including the need for consensus driven guidelines (none were proposed)

For peer review only - http://bmjopen.bmj.com/site/about/guidelines.xhtml

For peer review only

Table 1 continued

Author, year	Guideline	Research design	Description
Norquist et al. [42]	Choice of recall period for patient-reported outcome measures: criteria for consideration	Literature review	Choice of recall period for a PRO measure depends on nature of the disease, stability of symptoms, and trajectory of symptoms over time
Revicki et al. [12]	Recommendations on health-related quality of life research to support labeling and promotional claims in the United States	Review	Outlines the importance of an evidentiary base for making claims with respect to medical labeling or promotional claims
Revicki et al. [7]	Documenting the rationale and psychometric characteristics of patient-reported outcomes for labeling and promotional claims: the PRO Evidence Dossier	Report	Describes the purpose and content of a PRO measure Evidence Dossier, as well as its potential role with respect to regulatory review
Revicki et al. [34]	Recommended methods for determining responsiveness and minimally important differences for patient-reported outcomes	Literature review and expert opinion	Makes concrete recommendations regarding estimation of minimally important differences (MID), which should be based on patient-based and clinical anchors and convergence across multiple approaches and methods
Rothman et al. [30]	Use of existing patient-reported outcome (PRO) instruments and their modification	Expert opinion	Discusses key issues regarding the assessment and documentation of content validity for an existing instrument; discusses potential threats to content validity and methods to ameliorate
Schmidt et al. [54]	Current issues in cross-cultural quality of life instrument development	Literature review	Provides an overview of cross-cultural adaptation of PRO measure and provides broad development guidelines, as well as a call for additional focus on international research
Schunemann et al. [8]	Interpreting the results of patient-reported outcome measures in clinical trials: The clinician's perspective	Report based on examples	The authors provided several examples to describe how to attach meaning to PROM score thresholds and/or score differences
Scientific Advisory Committee of Medical Outcomes Trust [2]	Assessing health status and quality of life instruments: attributes and review criteria	Expert opinion	Describes 8 key attributes of PRO measures, including conceptual and measurement model, reliability, validity, responsiveness, interpretability, respondent and administrative burden, alternate forms, and cultural and language adaptations
Sprangers et al. [55]	Assessing meaningful change in quality of life over time: a users' guide for clinicians	Literature review and expert opinion	Proposes a set of guidelines/questions to help guide clinicians as to how to use PRO data in the treatment decision process
Snyder et al. [5]	Implementing patient-reported outcomes assessment in clinical practice: a review of the options and considerations	Literature review	The ISOQOL group developed a series of options and considerations to help guide the use of PROs in clinical practice, along with strengths and weaknesses of alternate approaches
Turner et al. [56]	Patient-reported outcomes: Instrument development and selection issues	Literature review	Provides a broad summary of concepts and issues to consider in the development and selection of a PRO measure
United States Food and Drug Administration [1]	Guidance for Industry: Patient-reported outcome measures: use in medical product development to support drug labeling claims	Expert opinion	"This guidance describes how the Food and Drug Administration (FDA) reviews and evaluates existing, modified, or newly created *patient-reported outcome instruments* used to support *claims* in approved medical product labeling." It covers conceptual frameworks, content validity, reliability, validity, ability to detect change, modification of PRO, and use of PRO in special populations

For peer review only - http://bmjopen.bmj.com/site/about/guidelines.xhtml

Table 1 continued

Author, year	Guideline	Research design	Description
Wild et al. [29]	Principles of good practice for the translation and cultural adaptation process for patient-reported outcomes measures	Literature review and expert opinion/ consensus	The ISPOR Task Force produced a critique of the strengths and weaknesses of various methods for translation and cultural adaptation of PROMS
Wild et al. [31]	Multinational trials-recommendations on the translations required, approaches to using the same language in different countries, and the approaches to support pooling the data	Expert opinion and literature review	Provides decision tools to decide on translation required for PRO measure; approach to use when same language is spoken in more than one country; and methods to gather evidence to support pooling of data across different language versions
Wyrwich et al. [38]	Methods for interpreting change over time in patient-reported outcome measures	Literature review	This article reviews the evolution of the methods and the terminology used to describe and aid in the communication of meaningful PRO change score thresholds

(86 % of these from the United States) and 33 % from Europe.

The participants reported being skilled in qualitative and quantitative methods and felt comfortable providing guidance for recommendations for PRO measurement standards. Approximately 81 % of the sample reported they had moderate to extensive training in quantitative methods and 53 % reported they had moderate to extensive training in qualitative methods. Overall, 89 % reported they felt competent or very competent providing guidance. As a sensitivity analysis, we examined the endorsement of recommendations excluding the 11 % who felt only somewhat or a little competent, but this resulted in no changes for our final recommendations. On average, the sample had 15 years of PRO measurement and research experience in the field.

Minimum standards for selecting a PRO measure for use in PCOR

Table 3 provides definitions of the properties of a PRO measure, and Table 4 provides an overview of the results from the ISOQOL survey on draft recommendations for minimal standards. Table 5 provides final recommendations based on these results and the feedback from ISOQOL members. A review of the findings from our literature review and survey is provided below.

Conceptual and measurement model

ISOQOL members were very supportive of the minimum standards described in Table 4 (#1) with 90 % of respondents endorsing the statement that a PRO measure should have documentation that defines the PRO construct and describes the intended application of the measure in the intended population. Also, 61 % of respondents agreed the documentation should describe how the measured concept(s) are operationalized in the measurement model.

Reliability of a PRO measure

A majority of ISOQOL respondents agreed that as a minimum standard a multi-item PRO measure should be assessed for internal consistency reliability, and a single-item PRO measure should be assessed by test–retest reliability (see Table 4, #2). However, they did not support as a minimum standard that a multi-item PRO measure should be required to have evidence of test–retest reliability. They noted practical concerns regarding test–retest reliability; primarily that some populations studied in PCOR are not stable and that their HRQOL can fluctuate. This phenomenon would reduce estimates of test–retest reliability, making the PRO measure look unreliable when it may be accurately detecting changes over time. In addition, memory effects will positively influence the test–retest reliability when the two survey points are scheduled close to each other.

Respondents endorsed the minimum level of reliability of 0.70 for group-level comparisons, which is commonly accepted in the field [2, 40, 41]. The standard error of measurement at this reliability level is approximately 0.55 of a standard deviation. However, there were concerns that establishing an absolute cut-off would be too prescriptive (e.g., a PRO measure with an estimated reliability coefficient of 0.69 would be deemed unreliable). Some respondents (36 %) supported the statement that "no minimum level of reliability should be stated; however, the reliability should be appropriately justified for the context of the proposed PRO measurement application."

For peer review only - http://bmjopen.bmj.com/site/about/guidelines.xhtml

Table 2 Participant-reported sample characteristics

Sample characteristic	% ($n = 120$)
Degrees[a]	
MD	18 %
PhD/Other Doctoral Degree (e.g., ScD)	64 %
RN/NP	5 %
Physical/Occupational Therapist	7 %
MA, MSc, MPH, or other Master's	43 %
Role[a]	
Academic Researcher	68 %
Clinician	21 %
Industry Representative	8 %
Industry Consultant/CRO Employee	23 %
Federal Government Employee	6 %
Patient Advocate	2 %
Other	8 %
Geographic location	
North America	48 %
United States	(86 %)
Europe	33 %
South America	5 %
Asia	10 %
Africa	1 %
Australia	3 %
Quantitative training in PRO measure design and evaluation	
Extensive training	37 %
Moderate amount of training	44 %
A little training	16 %
Not any training	3 %
Qualitative training in PRO measure design and evaluation	
Extensive training	18 %
Moderate amount of training	35 %
A little training	40 %
Not any training	7 %
Competency	
Very competent	50 %
Competent	39 %
Somewhat competent	8 %
A little competent	3 %
Average number of years in health-related quality (HRQOL) or patient-reported outcomes (PROs) field	
Mean years in HRQOL or PRO field	15 years; (range 1–40 years)

[a] More than one response was allowed for this characteristic

Validity of a PRO measure

The most common types of validity that were considered for minimum standards were content validity, construct validity, and responsiveness. Responsiveness is often regarded as an aspect of validity [4, 37]; however, it is often discussed separately given its importance to PRO measurement in longitudinal studies [4]. Criterion-related validity was not considered since there is generally no "gold standard" to which to compare a PRO measure. In the survey of ISOQOL members, only 7 and 10 % felt criterion-related validity was critical to have for a PRO measure in a cross-sectional or longitudinal study, respectively. It should be noted that the APA standards manual [32] suggests that validity is a unitary concept including all aspects of validity. However, the field of

For peer review only - http://bmjopen.bmj.com/site/about/guidelines.xhtml

Table 3 Definition of PRO measure properties

Conceptual and measurement model—The conceptual model provides a description and framework for the targeted construct(s) to be included in a PRO measure. The measurement model maps the individual items in the PRO measure to the construct

Reliability—The degree to which a PRO measure is free from measurement error [2, 4, 40, 41]

Internal consistency reliability—The degree of the interrelatedness among the items in a multi-item PRO measure [2, 4]

Test–retest reliability—A measure of the reproducibility of the scale, that is, the ability to provide consistent scores over time in a stable population [2]

Validity—The degree to which a PRO instrument measures the PRO concept it purports to measure [2, 4, 41]

Content validity—The extent to which the PRO measure includes the most relevant and important aspects of a concept in the context of a given measurement application [50]

Construct validity—The degree to which scores on the PRO measure relate to other measures (e.g., patient-reported or clinical indicators) in a manner that is consistent with theoretically derived a priori hypotheses concerning the concepts that are being measured [40]

Criterion validity—The degree to which the scores of a PRO measure are an adequate reflection of a "gold standard." [4]

Responsiveness—The extent to which a PRO measure can detect changes in the construct being measured over time [2, 37]

Interpretability of scores—The degree to which one can assign easily understood meaning to a PRO measure's scores [2, 4]

Minimal important difference (*MID*)—The smallest difference in score in the outcome of interest that informed patients or informed proxies perceive as important, either beneficial or harmful, and that would lead the patient or clinician to consider a change in the management [44, 57, 58]

Burden—The time, effort, and other demands placed on those to whom the instrument is administered (respondent burden) or on those who administer the instrument (investigator or administrative burden) [2]

outcomes research still distinguishes the above terms, probably because different methodologies are needed to address different forms of validity.

Content validity was rated as one of the most critical forms of validity to be assessed for a PRO measure with 58 and 61 % of ISOQOL members indicating a PRO measure must have evidence for content validity before using it in a cross-sectional or longitudinal study, respectively (data not shown in Table 4) [1]. Although the recommendations for minimum standards for content validity were endorsed by ISOQOL members (see Table 4, #3a), there was disagreement about the recall period, which is the period of time of reference (e.g., currently, past 24 h, past 7 days, past 4 weeks) for patients to describe their experiences with the measured PRO. Most (52 %) believed that a justification for the recall period was desirable but not required as a minimum standard for a PRO measure. In the final recommendation, we recommend that the reference period must be considered carefully in order for research participants to provide valid responses. However, we do not recommend a single recall period as it varies depending on the PRO domain being measured, the research context, and the population being studied [42].

Another aspect of content validity has to do with the provenance of items. One statement that was considered as a minimum standard but not supported by ISOQOL members was for the "documentation of sources from which items were derived, modified, and prioritized during the PRO measure development process." Because a majority of respondents felt this standard was important (46 % voted "required as minimum standard" and 46 % voted "desirable but not required"), we recommend this documentation be considered as a "best practice" but not a minimum standard for PRO measures.

Construct validity was also judged a critical component of validity. A majority of respondents (55 %) judged documentation of empirical findings supporting a priori hypotheses regarding expected associations among similar and dissimilar measures to be a minimal standard for a PRO measure (see Table 4, #3b). Another part of our original recommendation considered documented evidence for "known groups" validity, requiring empirical findings that support predefined hypotheses of the expected differences in scores between "known" groups. We considered this to be an important part of the evaluation of construct validity as it demonstrates the ability of a PRO measure to distinguish between one group and another where there is past empirical evidence of differences between the groups. However, the majority of ISOQOL members (57 %) rated it as a desirable but not required standard. Therefore, we considered this as a standard for "best practice" rather than a minimum standard.

Responsiveness, also referred to as longitudinal validity, is an aspect of construct validity [23, 37, 43]. A majority of ISOQOL respondents supported minimum standards of obtaining empirical evidence of changes in scores consistent with predefined hypotheses prior to using the PRO measure in longitudinal research (see Table 4, #3c). However, 65 % of respondents reported that they would use a PRO measure in a longitudinal study even if there was no prior study to support the responsiveness of the scale, but did have scientific evidence in a cross-sectional study of the reliability, content validity, and construct validity of the PRO measure.

For peer review only - http://bmjopen.bmj.com/site/about/guidelines.xhtml

Table 4 ISOQOL survey results on draft recommendations

	Draft recommendation for minimal standards	Survey results (n = 120)
1	*Conceptual and measurement model*	
	A PRO measure should have documentation defining and describing the concept(s) included and the intended population(s) for use	Required as a minimum standard—90 %<br>Desirable but not required as a minimum standard—9 %<br>Not required—0 %<br>Not sure—1 %<br>No opinion—0 %
	In addition, there should be documentation of how the concept(s) are organized into a measurement model, including evidence for the dimensionality of the measure, how items relate to each measured concept, and the relationship among concepts included in the PRO measure	Required as a minimum standard—61 %<br>Desirable but not required—35 %<br>Not required—3 %<br>Not sure—1 %<br>No opinion—0 %
2	*Reliability*	
	The reliability of a PRO measure should ideally be at or above 0.70 for group-level comparisons	Yes, it should be at or above 0.70—54 %<br>No, it should be at or above _fill in blank_—8 % (responses ranged from 0.50 to 0.80)<br>No minimum level of reliability should be appropriately justified for the context of the proposed application—36 %<br>No opinion—2 %
	Reliability for a multi-item unidimensional scale should include an assessment of internal consistency	Required as a minimum standard—79 %<br>Desirable but not required—14 %<br>Not required—2 %<br>Not sure—3 %<br>No opinion—2 %
	Reliability for a multi-item unidimensional scale should include an assessment of test–retest reliability	Required as a minimum standard—43 %<br>Desirable but not required—51 %<br>Not required—3 %<br>Not sure—3 %<br>No opinion—0 %
	Reliability for a single-item measure should be assessed by test–retest reliability	Required as a minimum standard—60 %<br>Desirable but not required—34 %<br>Not required—2 %<br>Not sure—3 %<br>No opinion—1 %
3	*Validity*	
3a	*Content validity*	
	A PRO measure should have evidence supporting its content validity, including evidence that patients and/or experts consider the content of the PRO measure relevant and comprehensive for the concept, population, and aim of the measurement application	Required as a minimum standard—78 %<br>Desirable but not required—19 %<br>Not required—2 %<br>Not sure—0 %<br>No opinion—1 %
	Documentation of qualitative and/or quantitative methods used to solicit and confirm attributes (i.e., concepts measured by the items) of the PRO relevant to the measurement application	Required as a minimum standard—53 %<br>Desirable but not required—44 %<br>Not required—2 %<br>Not sure—1 %<br>No opinion—0 %

For peer review only

For peer review only - http://bmjopen.bmj.com/site/about/guidelines.xhtml

Table 4 continued

	Draft recommendation for minimal standards	Survey results (n = 120)
	Documentation of the characteristics of participants included in the evaluation (e.g., race/ethnicity, culture, age, socio-economic status, literacy)	Required as a minimum standard—52 %<br>Desirable but not required—47 %<br>Not required—0 %<br>Not sure—0 %<br>No opinion—1 %
	Documentation of sources from which items were derived, modified, and prioritized during the PRO measure development process	Required as a minimum standard—46 %<br>Desirable but not required—46 %<br>Not required—7 %<br>Not sure—0 %<br>No opinion—1 %
	Justification for the recall period for the measurement application	Required as a minimum standard—41 %<br>Desirable but not required—52 %<br>Not required—5 %<br>Not sure—1 %<br>No opinion—1 %
3b	*Construct validity*	
	A PRO measure should have evidence supporting its construct validity, including documentation of empirical findings that support predefined hypotheses on the expected associations among measures similar or dissimilar to the measured PRO	Required as a minimum standard—55 %<br>Desirable but not required—44 %<br>Not required—1 %<br>Not sure—0 %<br>No opinion—0 %
	A PRO measure should have evidence supporting its construct validity, including documentation of empirical findings that support predefined hypotheses of the expected differences in scores between "known" groups	Required as a minimum standard—41 %<br>Desirable but not required—57 %<br>Not required—2 %<br>Not sure—0 %<br>No opinion—0 %
3c	*Responsiveness*	
	A PRO measure for use in longitudinal research study should have evidence of responsiveness, including empirical evidence of changes in scores consistent with predefined hypotheses regarding changes in the target population for the research application	Required as a minimum standard—57 %<br>Desirable but not required—42 %<br>Not required—1 %<br>Not sure—0 %<br>No opinion—0 %
	If a PRO measure has cross-sectional data that provide sufficient evidence in regard to the reliability (internal consistency), content validity, and construct validity but has no data yet on responsiveness over time (i.e., ability of a PRO measure to detect changes in the construct being measured over time), would you accept use of the PRO measure to provide valid data over time in a longitudinal study if no other PRO measure was available?	Yes—65 %<br>No, I would require evidence of responsiveness before accepting it—32 %<br>No opinion—0 %<br>Comments (fill in blank response)—22 %
4	*Interpretability of Scores*	
	A PRO measure should have documentation to support interpretation of scores, including what low and high scores represent for the measured concept	Required as a minimum standard—64 %<br>Desirable but not required—35 %<br>Not required—1 %<br>Not sure—0 %<br>No opinion—0 %
	A PRO measure should have documentation to support interpretation of scores, including representative mean(s) and standard deviation(s) in the reference population	Required as a minimum standard—39 %<br>Desirable but not required—57 %<br>Not required—4 %<br>Not sure—0 %<br>No opinion—0 %

For peer review only - http://bmjopen.bmj.com/site/about/guidelines.xhtml

Table 4 continued

	Draft recommendation for minimal standards	Survey results (n = 120)
	A PRO measure should have documentation to support interpretation of scores, including guidance on the minimally important difference in scores between groups and/or over time that can be considered meaningful from the patient and/or clinical perspective	Required as a minimum standard—23 %<br>Desirable but not required—72 %<br>Not required—5 %<br>Not sure—0 %<br>No opinion—0 %
5	*Translation of a PRO measure*	
	A PRO measure translated to one or more languages should have evidence of the equivalence of measurement properties for translated versions, allowing comparison or combination of data across language forms	Required as a minimum standard—47 %<br>Desirable but not required—49 %<br>Not required—4 %<br>Not sure—0 %<br>No opinion—0 %
	Documentation of background and experience of the persons involved in the translation	Required as a minimum standard—43 %<br>Desirable but not required—49 %<br>Not required—8 %<br>Not sure—0 %<br>No opinion—0 %
	Documentation of methods used to translate and evaluate the PRO measure in each language	Required as a minimum standard—81 %<br>Desirable but not required—16 %<br>Not required—3 %<br>Not sure—0 %<br>No opinion—0 %
	Documentation of extent of harmonization across different language versions	Required as a minimum standard—38 %<br>Desirable but not required—53 %<br>Not required—7 %<br>Not sure—2 %<br>No opinion—0 %
6	*Patient and investigator Burden*	
	The reading level of the PRO measure for research involving adult respondents from the general population should be at a minimum of...	4th grade education level—7 %<br>6th grade education level—23 %<br>8th grade education level—6 %<br>Other grade level ____—8 %<br>There should be no minimum requirement of the literacy level of the PRO measure; however, it should be appropriately justified for the context of it proposed application—43 %<br>Not sure—9 %<br>No opinion—4 %

For peer review only

Interpretability of scores

For a PRO measure to be well accepted for the use in PCOR, it must provide scores that are easily interpreted by different stakeholders including patients, clinicians, researchers, and policy makers [38]. The literature review revealed several ways to enhance interpretability of scores that may be considered for standard setting. End-users must be able to know what a high or low score represents. In addition, knowing what comprises a meaningful difference or change in the score from one group to another (or one time to another) would enhance understanding of the outcome being measured. Another way to enhance the interpretability of PRO measure scores would involve comparing scores from a study to known scores in a population (e.g., the general US population or a specific disease population). The availability of such benchmarks would enhance understanding of how the study group scored as compared to some reference or normative group.

A majority of respondents endorsed as a minimum standard that a PRO measure should have documentation to support the interpretation of scores including description of what low and high scores represent (see Table 4, #4). However, more useful metrics such as norm or reference

For peer review only - http://bmjopen.bmj.com/site/about/guidelines.xhtml

Table 5 Final recommendations for minimum standards for patient-reported outcome (PRO) measures used in patient-centered outcomes research or comparative effectiveness research

1	*Conceptual and measurement model*—A PRO measure should have documentation defining and describing the concept(s) included and the intended population(s) for use. In addition, there should be documentation of how the concept(s) are organized into a measurement model, including evidence for the dimensionality of the measure, how items relate to each measured concept, and the relationship among concepts included in the PRO measure
2	*Reliability*—The reliability of a PRO measure should preferably be at or above 0.70 for group-level comparisons, but may be lower if appropriately justified. Reliability can be estimated using a variety of methods including internal consistency reliability, test–retest reliability, or item response theory. Each method should be justified
3	*Validity*
3a	*Content validity*—A PRO measure should have evidence supporting its content validity, including evidence that patients and experts consider the content of the PRO measure relevant and comprehensive for the concept, population, and aim of the measurement application. This includes documentation of as follows: (1) qualitative and/or quantitative methods used to solicit and confirm attributes (i.e., concepts measured by the items) of the PRO relevant to the measurement application; (2) the characteristics of participants included in the evaluation (e.g., race/ethnicity, culture, age, gender, socio-economic status, literacy level) with an emphasis on similarities or differences with respect to the target population; and (3) justification for the recall period for the measurement application
3b	*Construct validity*—A PRO measure should have evidence supporting its construct validity, including documentation of empirical findings that support predefined hypotheses on the expected associations among measures similar or dissimilar to the measured PRO
3c	*Responsiveness*—A PRO measure for use in longitudinal research study should have evidence of responsiveness, including empirical evidence of changes in scores consistent with predefined hypotheses regarding changes in the measured PRO in the target population for the research application
4	*Interpretability of scores*—A PRO measure should have documentation to support interpretation of scores, including what low and high scores represent for the measured concept
5	*Translation of the PRO measure*—A PRO measure translated to one or more languages should have documentation of the methods used to translate and evaluate the PRO measure in each language. Studies should at least include evidence from qualitative methods (e.g., cognitive testing) to evaluate the translations
6	*Patient and investigator Burden*—A PRO measure must not be overly burdensome for patients or investigators. The length of the PRO measure should be considered in the context of other PRO measures included in the assessment, the frequency of PRO data collection, and the characteristics of the study population. The literacy demand of the items in the PRO measure should usually be at a 6th grade education level or lower (i.e., 12 year old or lower); however, it should be appropriately justified for the context of the proposed application

scores or minimally important difference (MID) estimates were not considered required, but were considered highly desirable [34, 44, 45].

Translation of a PRO measure

PCOR and CER are often carried out in multi-national or multi-cultural settings that require the PRO measure to be translated into different languages. To be able to compare or combine HRQOL results across those groups, it is critical that the measured HRQOL concept and the wording of the questionnaire used to measure it is interpreted in the same way across translations [29, 46].

Of the original draft recommendations reviewed in the survey (see Table 4, #5), ISOQOL members supported as a minimum standard the statement, "Documentation of methods used to translate and evaluate the PRO measure in each language." In response to follow-up questions (not summarized in Table 4), 41 % of respondents considered it necessary, while 40 % felt it was expected but not required, to employ qualitative methods (e.g., cognitive interviews) for reviewing the quality of translations before using a translated PRO measure. Only 24 % of respondents thought that quantitative methods should be required for reviewing the quality of the translations (e.g., differential item functioning testing) before using the PRO measure, and 42 % of respondents indicated that it was expected (but not absolutely necessary) to include quantitative evaluation before they would use a translated PRO measure. Based on these findings, the ISOQOL SATF recommended that qualitative evidence be included as a minimum standard for translated PRO measures (Table 5).

Patient and investigator Burden

The committee agreed that burden on patients and investigators must be considered when selecting PRO measures for a PCOR study. A PRO measure must not be overly burdensome for patients as they are often ill and should not be subjected to overly long questionnaires or too frequent data collection that disrupts their lives. Ninety-two percent of the survey respondents concurred, endorsing "respondent burden" as an important or very important consideration for selecting PRO measures for PCOR.

Similarly, 90 % of respondents endorsed literacy as an important or very important consideration in selecting PRO measures in PCOR. Data collected from PRO measures are

For peer review only - http://bmjopen.bmj.com/site/about/guidelines.xhtml

only valid if the participants in a study can understand what is being asked of them and can provide a response that accurately reflects their experiences or perspectives. It is critical that developers of PRO measures ensure the questions, and response options are clear and easy to understand. Qualitative testing of the PRO measure (e.g., cognitive interviewing) should include individuals with low literacy to evaluate the questions [47]. Twenty-three percent of respondents indicated that a PRO measure should be written at 6th grade education level (ages 11–12 years), while 43 % indicated that the literacy level should be appropriately justified for the given research application.

Discussion

Based on a literature review of existing guidelines and a survey of experts in PRO measurement and research, we, on behalf of the ISOQOL, put forth minimum standards for PRO measures to be used in patient-centered outcomes research and comparative effectiveness research. These recommendations include the documentation of the characteristics of the conceptual and measurement model, evidence for reliability, validity, and interpretability of scores, quality translations, and acceptable patient and investigator burden (summarized in Table 5). The extent to which a PRO measure adheres to the standards described in this report reflects the quality of the PRO measurement.

Good documentation of the evidence that a PRO measure meets and exceeds these measurement properties will result in greater acceptance of the PRO measure for use in PCOR and CER. This documentation could include a focused methodologically rigorous study of the measurement properties of the PRO measure or analysis of HRQOL data collected from the PRO measure within a PCOR or CER study. Such documentation should be made available in peer-reviewed literature as well as on publically accessible websites. To the extent that the evidence was obtained from populations similar to the target population in the study, the investigator(s) will have greater confidence in the PRO measure to capture patients' experiences and perspectives.

There are a number of considerations when applying these minimum standards in PCOR and CER. The populations participating in PCOR and CER will likely be more heterogeneous than those that are typically included in phase II or III clinical trials. This population heterogeneity should be reflected in the samples included in the evaluation of the measurement properties for the PRO measure. For example, both qualitative and quantitative studies may require quota sampling based on race/ethnicity, gender, or age groups that reflect the prevalence of the condition in the study target population.

Researchers must consider carefully the strength of evidence supporting the measurement properties of the PRO measure. There is no threshold for which an instrument is valid or not valid for all populations or applications. In addition, no single study can confirm all the measurement properties for all research contexts. Like all scientific disciplines, measurement science relies on the iterative accumulation of a body of evidence (maturation model), replicated in different settings. Thus, it is the weight of the evidence (i.e., the number and quality of the studies and consistency of findings) that informs the evaluation of the appropriateness of a PRO measure. Older PRO measures will sometimes have the benefit of having more evidence than newer measures, and this will be reflected in the standards.

A possible limitation of this study is the potential for the biases of individual members of the SATF to influence the survey content. The transparency of the process used, and the wide variety of expertise and perspectives among the members, mitigated against substantive bias being introduced. In addition, the response rate to the survey was modest, again indicating the potential for bias. We point out, however, that the demographic data collected on the survey indicated that the respondents were experienced ISOQOL members with a variety of professional perspectives, the vast majority of whom self-identified as being competent in providing ratings and responses for the survey items.

These minimum standards were created by ISOQOL to reflect when a PRO measure may be considered appropriate or inappropriate for a specific PCOR study; thus, the intent was to have a minimum standard by which PRO measures could be judged acceptable. These standards do not reflect "ideal standards" or "best practices," which will have more stringent criteria [2, 3, 40]. For example, established minimally important differences for a PRO measure will enhance the interpretability of scores to inform decision making. As another example, establishing measurement equivalence of the PRO across different modes of assessment (e.g., paper forms, computers, handheld devices, phone) may facilitate broader patient participation in PCOR. ISOQOL's recommendations for "best practices" for PRO measures in PCOR and CER will be a next step in the organization's strategic initiative to advance the science of HRQOL measurement.

The findings from this study were reviewed by the PCORI Methodology Committee as part of that Committee's review of relevant standards and guidelines pertinent to patient-centered outcomes research. The ISOQOL recommendations presented here focus on more specific information about PRO measurement properties than those found in the PCORI Methodology Committee standards [15].

For peer review only - http://bmjopen.bmj.com/site/about/guidelines.xhtml

The identification and selection of PRO measures meeting and exceeding these current ISOQOL recommended minimum standards will increase the likelihood that the evidence generated in PCOR and CER reliably and validly represents the patients' perspective on health-related outcomes. This PRO evidence, based on instruments with sound measurement properties, can then be used to inform clinical and health policy decision making about the benefits and risks associated with different health interventions or to monitor population health.

Acknowledgments This study was funded by the Patient-Centered Outcomes Research Institute (PCORI-SOL-RMWG-001: PIs: Zeeshan Butt, PhD, Northwestern University; Bryce Reeve, PhD, University of North Carolina at Chapel Hill). The views expressed in this article are those of the authors and do not necessarily reflect those of PCORI.

References

1. US Food and Drug Administration. (2009). Patient-reported outcome measures: Use in medical product development to support labeling claims. Guidance for industry. http://www.fda.gov/downloads/Drugs/GuidanceComplianceRegulatoryInformation/Guidances/UCM071975.pdf. Accessed November 26, 2011.
2. Scientific Advisory Committee of the Medical Outcomes Trust. (2002). Assessing health status and quality-of-life instruments: Attributes and review criteria. *Quality of Life Research, 11*(3), 193–205.
3. Mokkink, L. B., Terwee, C. B., Knol, D. L., Stratford, P. W., Alonso, J., Patrick, D. L., et al. (2006). Protocol of the COSMIN study: COnsensus-based standards for the selection of health measurement INstruments. *BMC Medical Research Methodology, 6*, 2. doi:10.1186/1471-2288-6-2.
4. Mokkink, L. B., Terwee, C. B., Patrick, D. L., Alonso, J., Stratford, P. W., Knol, D. L., et al. (2010). The COSMIN study reached international consensus on taxonomy, terminology, and definitions of measurement properties for health-related patient-reported outcomes. *Journal of Clinical Epidemiology, 63*(7), 737–745. doi:10.1016/j.jclinepi.2010.02.006.
5. Snyder, C. F., Aaronson, N. K., Choucair, A. K., Elliott, T. E., Greenhalgh, J., Halyard, M. Y., et al. (2011). Implementing patient-reported outcomes assessment in clinical practice: A review of the options and considerations. *Quality of Life Research,*. doi:10.1007/s11136-011-0054-x.
6. Basch, E. M., Reeve, B. B., Mitchell, S. A., Clauser, S. B., Minasian, L., Sit, L., et al. (2011). Electronic toxicity monitoring and patient-reported outcomes. *Cancer Journal, 17*(4), 231–234. doi:10.1097/PPO.0b013e31822c28b3.
7. Revicki, D. A., Gnanasakthy, A., & Weinfurt, K. (2007). Documenting the rationale and psychometric characteristics of patient reported outcomes for labeling and promotional claims: The PRO Evidence Dossier. *Quality of Life Research, 16*(4), 717–723. doi:10.1007/s11136-006-9153-5.
8. Schunemann, H. J., Akl, E. A., & Guyatt, G. H. (2006). Interpreting the results of patient reported outcome measures in clinical trials: The clinician's perspective. *Health and Quality of Life Outcomes, 4*, 62. doi:10.1186/1477-7525-4-62.
9. Deyo, R. A., & Patrick, D. L. (1989). Barriers to the use of health status measures in clinical investigation, patient care, and policy research. *Medical Care, 27*(3 Suppl), S254–S268.
10. International Society for Quality of Life Research. http://www.isoqol.org/. Accessed July 30, 2012.
11. Guyatt, G., & Schunemann, H. (2007). How can quality of life researchers make their work more useful to health workers and their patients? *Quality of Life Research, 16*(7), 1097–1105. doi:10.1007/s11136-007-9223-3.
12. Revicki, D. A., Osoba, D., Fairclough, D., Barofsky, I., Berzon, R., Leidy, N. K., et al. (2000). Recommendations on health-related quality of life research to support labeling and promotional claims in the United States. *Quality of Life Research, 9*(8), 887–900.
13. Lipscomb, J., Donaldson, M. S., Arora, N. K., Brown, M. L., Clauser, S. B., Potosky, A. L., et al. (2004). Cancer outcomes research. *Journal of the National Cancer Institute Monographs* (33), 178–197. doi:10.1093/jncimonographs/lgh039.
14. US Patient-Centered Outcomes Research Institute http://www.pcori.org. Accessed 26 November, 2011.
15. Methodology Committee of the Patient-Centered Outcomes Research, I. (2012). Methodological standards and patient-centeredness in comparative effectiveness research: The PCORI perspective. *Journal of the American Medical Association, 307*(15), 1636–1640. doi:10.1001/jama.2012.466.
16. Terwee, C. B., Jansma, E. P., Riphagen, I. I., & de Vet, H. C. (2009). Development of a methodological PubMed search filter for finding studies on measurement properties of measurement instruments. *Quality of Life Research, 18*(8), 1115–1123. doi:10.1007/s11136-009-9528-5.
17. Qualtrics Labs Inc. Why choose qualtrics survey software? https://www.qualtrics.com/why-survey-software. Accessed November 26, 2011.
18. US Food and Drug Administration. (2010). Qualification process for drug development tools. Draft Guidance for Industry. http://www.fda.gov/downloads/Drugs/GuidanceComplianceRegulatoryInformation/Guidances/UCM230597.pdf. Accessed November 26, 2011.
19. Erickson, P., Willke, R., & Burke, L. (2009). A concept taxonomy and an instrument hierarchy: Tools for establishing and evaluating the conceptual framework of a patient-reported outcome (PRO) instrument as applied to product labeling claims. *Value in Health, 12*(8), 1158–1167. doi:10.1111/j.1524-4733.2009.00609.x.
20. Patrick, D. L., Burke, L. B., Powers, J. H., Scott, J. A., Rock, E. P., Dawisha, S., et al. (2007). Patient-reported outcomes to support medical product labeling claims: FDA perspective. *Value in Health, 10*(Suppl 2), S125–S137. doi:10.1111/j.1524-4733.2007.00275.x.
21. Angst, F. (2011). The new COSMIN guidelines confront traditional concepts of responsiveness. *BMC Medical Research Methodology, 11*, 152; author reply 152. doi:10.1186/1471-2288-11-152.
22. Mokkink, L. B., Terwee, C. B., Gibbons, E., Stratford, P. W., Alonso, J., Patrick, D. L., et al. (2010). Inter-rater agreement and reliability of the COSMIN (COnsensus-based standards for the selection of health status measurement instruments) checklist. *BMC Medical Research Methodology, 10*, 82. doi:10.1186/1471-2288-10-82.
23. Mokkink, L. B., Terwee, C. B., Knol, D. L., Stratford, P. W., Alonso, J., Patrick, D. L., et al. (2010). The COSMIN checklist for evaluating the methodological quality of studies on measurement properties: A clarification of its content. *BMC Medical Research Methodology, 10*, 22. doi:10.1186/1471-2288-10-22.
24. Mokkink, L. B., Terwee, C. B., Patrick, D. L., Alonso, J., Stratford, P. W., Knol, D. L., et al. (2010). The COSMIN checklist for assessing the methodological quality of studies on measurement properties of health status measurement instruments: An international Delphi study. *Quality of Life Research, 19*(4), 539–549. doi:10.1007/s11136-010-9606-8.
25. Terwee, C. B., Mokkink, L. B., Knol, D. L., Ostelo, R. W., Bouter, L. M., & de Vet, H. C. (2012). Rating the methodological

For peer review only - http://bmjopen.bmj.com/site/about/guidelines.xhtml

quality in systematic reviews of studies on measurement properties: A scoring system for the COSMIN checklist. *Quality of Life Research, 21*(4), 651–657. doi:10.1007/s11136-011-9960-1.

26. Johnson, C., Aaronson, N., Blazeby, J. M., Bottomley, A., Fayers, P., Koller, M., et al. (2011). EORTC Quality of life group: Guidelines for developing questionnaire modules. http://groups.eortc.be/qol/sites/default/files/archives/guidelines_for_developing_questionnaire-_final.pdf. Accessed November 26, 2011.
27. Cella, D. (1997). *Manual of the functional assessment of chronic illness therapy (FACIT) measurement system*. Evanston, IL: Northwestern University.
28. Coons, S. J., Gwaltney, C. J., Hays, R. D., Lundy, J. J., Sloan, J. A., Revicki, D. A., et al. (2009). Recommendations on evidence needed to support measurement equivalence between electronic and paper-based patient-reported outcome (PRO) measures: ISPOR ePRO good research practices task force report. *Value in Health, 12*(4), 419–429. doi:10.1111/j.1524-4733.2008.00470.x.
29. Wild, D., Grove, A., Martin, M., Eremenco, S., McElroy, S., Verjee-Lorenz, A., et al. (2005). Principles of good practice for the translation and cultural adaptation process for patient-reported outcomes (PRO) measures: Report of the ISPOR task force for translation and cultural adaptation. *Value in Health, 8*(2), 94–104. doi:10.1111/j.1524-4733.2005.04054.x.
30. Rothman, M., Burke, L., Erickson, P., Leidy, N. K., Patrick, D. L., & Petrie, C. D. (2009). Use of existing patient-reported outcome (PRO) instruments and their modification: The ISPOR good research practices for evaluating and documenting content validity for the use of existing instruments and their modification PRO task force report. *Value in Health, 12*(8), 1075–1083. doi:10.1111/j.1524-4733.2009.00603.x.
31. Wild, D., Eremenco, S., Mear, I., Martin, M., Houchin, C., Gawlicki, M., et al. (2009). Multinational trials-recommendations on the translations required, approaches to using the same language in different countries, and the approaches to support pooling the data: the ISPOR patient-reported outcomes translation and linguistic validation good research practices task force report. *Value in Health, 12*(4), 430–440. doi:10.1111/j.1524-4733.2008.00471.x.
32. Joint Committee on Standards for Educational and Psychological Testing of the American Educational Research Association, American Psychological Association, & National Council on Measurement in Education (1998). Standards for educational and psychological testing Washington DC: American Psychological Association.
33. Magasi, S., Ryan, G., Revicki, D., Lenderking, W., Hays, R. D., Brod, M., et al. (2011). Content validity of patient-reported outcome measures: Perspectives from a PROMIS meeting. *Quality of Life Research,*. doi:10.1007/s11136-011-9990-8.
34. Revicki, D., Hays, R. D., Cella, D., & Sloan, J. (2008). Recommended methods for determining responsiveness and minimally important differences for patient-reported outcomes. *Journal of Clinical Epidemiology, 61*(2), 102–109. doi:10.1016/j.jclinepi.2007.03.012.
35. Valderas, J. M., Ferrer, M., Mendivil, J., Garin, O., Rajmil, L., Herdman, M., et al. (2008). Development of EMPRO: A tool for the standardized assessment of patient-reported outcome measures. *Value in Health, 11*(4), 700–708. doi:10.1111/j.1524-4733.2007.00309.x.
36. Dewolf, L., Koller, M., Velikova, G., Johnson, C., Scott, N., & Bottomley, A. (2009). EORTC quality of life group: Translation procedure. http://groups.eortc.be/qol/sites/default/files/archives/translation_manual_2009.pdf. Accessed November 26, 2011.
37. Hays, R. D., & Hadorn, D. (1992). Responsiveness to change: An aspect of validity, not a separate dimension. *Quality of Life Research, 1*(1), 73–75.
38. Wyrwich, K. W., Norquist, J. M., Lenderking, W. R., Acaster, S., & the Industry Advisory Committee of International Society for Quality of Life, R. (2012). Methods for interpreting change over time in patient-reported outcome measures. *Quality of Life Research, 2012 Apr 17*. [Epub ahead of print.]. doi:10.1007/s11136-012-0175-x.
39. Ahmed, S., Berzon, R. A., Revicki, D., Lenderking, W., Moinpour, C. M., Basch, E., et al. (2012). The use of patient-reported outcomes (PRO) within comparative effectivenss research: Implications for clinical practice and healthcare policy. *Medical Care, 50*(12), 1060–1070.
40. Terwee, C. B., Bot, S. D., de Boer, M. R., van der Windt, D. A., Knol, D. L., Dekker, J., et al. (2007). Quality criteria were proposed for measurement properties of health status questionnaires. *Journal of Clinical Epidemiology, 60*(1), 34–42. doi:10.1016/j.jclinepi.2006.03.012.
41. Nunnally, J. C., & Berstein, I. H. (1994). *Psychometric theory* (3rd ed.). New York: McGraw-Hill.
42. Norquist, J. M., Girman, C., Fehnel, S., Demuro-Mercon, C., & Santanello, N. (2011). Choice of recall period for patient-reported outcome (PRO) measures: Criteria for consideration. *Quality of Life Research,*. doi:10.1007/s11136-011-0003-8.
43. Revicki, D. A., Cella, D., Hays, R. D., Sloan, J. A., Lenderking, W. R., & Aaronson, N. K. (2006). Responsiveness and minimal important differences for patient reported outcomes. *Health and Quality of Life Outcomes, 4*, 70. doi:10.1186/1477-7525-4-70.
44. Brozek, J. L., Guyatt, G. H., & Schunemann, H. J. (2006). How a well-grounded minimal important difference can enhance transparency of labelling claims and improve interpretation of a patient reported outcome measure. *Health and Quality of Life Outcomes, 4*, 69. doi:10.1186/1477-7525-4-69.
45. Norman, G. R., Sridhar, F. G., Guyatt, G. H., & Walter, S. D. (2001). Relation of distribution- and anchor-based approaches in interpretation of changes in health-related quality of life. *Medical Care, 39*(10), 1039–1047.
46. Koller, M., Kantzer, V., Mear, I., Zarzar, K., Martin, M., Greimel, E., et al. (2012). The process of reconciliation: Evaluation of guidelines for translating quality-of-life questionnaires. *Expert Review of Pharmacoeconomics & Outcomes Research, 12*(2), 189–197. doi:10.1586/erp.11.102.
47. Jordan, J. E., Osborne, R. H., & Buchbinder, R. (2011). Critical appraisal of health literacy indices revealed variable underlying constructs, narrow content and psychometric weaknesses. *Journal of Clinical Epidemiology, 64*(4), 366–379. doi:10.1016/j.jclinepi.2010.04.005.
48. Acquadro, C., Conway, K., Hareendran, A., & Aaronson, N. (2008). Literature review of methods to translate health-related quality of life questionnaires for use in multinational clinical trials. *Value in Health, 11*(3), 509–521. doi:10.1111/j.1524-4733.2007.00292.x.
49. Crosby, R. D., Kolotkin, R. L., & Williams, G. R. (2003). Defining clinically meaningful change in health-related quality of life. *Journal of Clinical Epidemiology, 56*(5), 395–407.
50. Frost, M. H., Reeve, B. B., Liepa, A. M., Stauffer, J. W., Hays, R. D., & Mayo, F. D. A. P.-R. O. C. M. G. (2007). What is sufficient evidence for the reliability and validity of patient-reported outcome measures? *Value in Health, 10*(Suppl 2), S94–S105. doi:10.1111/j.1524-4733.2007.00272.x.
51. Hays, R. D., & Woolley, J. M. (2000). The concept of clinically meaningful difference in health-related quality-of-life research. How meaningful is it? *PharmacoEconomics, 18*(5), 419–423.
52. Kemmler, G., Zabernigg, A., Gattringer, K., Rumpold, G., Giesinger, J., Sperner-Unterweger, B., et al. (2010). A new approach to combining clinical relevance and statistical significance for evaluation of quality of life changes in the individual patient. *Journal of Clinical Epidemiology, 63*(2), 171–179. doi:10.1016/j.jclinepi.2009.03.016.
53. Kottner, J., Audige, L., Brorson, S., Donner, A., Gajewski, B. J., Hrobjartsson, A., et al. (2011). Guidelines for reporting reliability

For peer review only - http://bmjopen.bmj.com/site/about/guidelines.xhtml

and agreement studies (GRRAS) were proposed. *Journal of Clinical Epidemiology, 64*(1), 96–106. doi:10.1016/j.jclinepi.2010.03.002.
54. Schmidt, S., & Bullinger, M. (2003). Current issues in cross-cultural quality of life instrument development. *Archives of Physical Medicine and Rehabilitation, 84*(4 Suppl 2), S29–S34. doi:10.1053/apmr.2003.50244.
55. Sprangers, M. A., Moinpour, C. M., Moynihan, T. J., Patrick, D. L., Revicki, D. A., & Clinical Significance Consensus Meeting Group. (2002). Assessing meaningful change in quality of life over time: A users' guide for clinicians. *Mayo Clinic Proceedings, 77*(6), 561–571. doi:10.4065/77.6.561.
56. Turner, R. R., Quittner, A. L., Parasuraman, B. M., Kallich, J. D., Cleeland, C. S., & Mayo, F. D. A. P.-R. O. C. M. G. (2007). Patient-reported outcomes: Instrument development and selection issues. *Value in Health, 10*(Suppl 2), S86–S93. doi:10.1111/j.1524-4733.2007.00271.x.
57. Schunemann, H. J., & Guyatt, G. H. (2005). Commentary–goodbye M(C)ID! Hello MID, where do you come from? *Health Services Research, 40*(2), 593–597. doi:10.1111/j.1475-6773.2005.00374.x.
58. Schunemann, H. J., Puhan, M., Goldstein, R., Jaeschke, R., & Guyatt, G. H. (2005). Measurement properties and interpretability of the Chronic respiratory disease questionnaire (CRQ). *Copd, 2*(1), 81–89.

For peer review only

For peer review only - http://bmjopen.bmj.com/site/about/guidelines.xhtml
